# Comparative analyses identify genomic features potentially involved in the evolution of birds-of-paradise

**DOI:** 10.1093/gigascience/giz003

**Published:** 2019-01-24

**Authors:** Stefan Prost, Ellie E Armstrong, Johan Nylander, Gregg W C Thomas, Alexander Suh, Bent Petersen, Love Dalen, Brett W Benz, Mozes P K Blom, Eleftheria Palkopoulou, Per G P Ericson, Martin Irestedt

**Affiliations:** 1 Department of Biodiversity and Genetics, Swedish Museum of Natural History, Frescativaegen 40, 114 18 Stockholm, Sweden; 2 Department of Integrative Biology, University of California, 3040 Valley Life Science Building, Berkeley, CA 94720-3140, USA; 3 Department of Biology, Stanford University, 371 Serra Mall, Stanford, CA 94305–5020, USA; 4 Department of Biology and School of Informatics, Computing, and Engineering, Indiana University, 1001 E. Third Street, Bloomington, IN 47405, USA; 5 Department of Evolutionary Biology (EBC), Uppsala University, Norbyvaegen 14-18, 75236 Uppsala, Sweden; 6 Natural History Museum of Denmark, University of Copenhagen, Oster Voldgade 5-7, 1353 Copenhagen, Denmark; 7 Centre of Excellence for Omics-Driven Computational Biodiscovery, Faculty of Applied Sciences, Asian Institute of Medicine, Science and Technology,Jalan Bedong-Semeling, 08100 Bedong, Kedah, Malaysia; 8 Department of Ornithology, American Museum of Natural History, Central Park West, New York, NY 10024, USA

**Keywords:** birds-of-paradise, comparative genomics, gene gain-loss, positive selection, genome structure

## Abstract

The diverse array of phenotypes and courtship displays exhibited by birds-of-paradise have long fascinated scientists and nonscientists alike. Remarkably, almost nothing is known about the genomics of this iconic radiation. There are 41 species in 16 genera currently recognized within the birds-of-paradise family (Paradisaeidae), most of which are endemic to the island of New Guinea. In this study, we sequenced genomes of representatives from all five major clades within this family to characterize genomic changes that may have played a role in the evolution of the group's extensive phenotypic diversity. We found genes important for coloration, morphology, and feather and eye development to be under positive selection. In birds-of-paradise with complex lekking systems and strong sexual dimorphism, the core birds-of-paradise, we found Gene Ontology categories for “startle response” and “olfactory receptor activity” to be enriched among the gene families expanding significantly faster compared to the other birds in our study. Furthermore, we found novel families of retrovirus-like retrotransposons active in all three *de novo* genomes since the early diversification of the birds-of-paradise group, which might have played a role in the evolution of this fascinating group of birds.

## Background

*“Every ornithologist and birdwatcher has his favourite group of birds. Frankly, my own are the birds of paradise and bowerbirds. If they do not rank as high in world-wide popularity as they deserve it is only because so little is known about them.”*

*Ernst Mayr (in Gilliard 1969* [1])

The spectacular morphological and behavioral diversity found in birds-of-paradise (Paradisaeidae) form one of the most remarkable examples in the animal kingdom of traits that are thought to have evolved via forces of sexual selection and female choice. The family is comprised of 41 recognized species divided into 16 genera [2], all of which are confined to the Australo-Papuan realm and the Moluccas islands (Indonesia). The birds-of-paradise have adapted to a wide variety of habitats ranging from tropical lowlands to high-altitude mountain forests [3] and, in the process, acquired a diverse set of morphological traits. Some species are sexually monomorphic and crow-like in appearance with simple mating systems, whereas others have complex courtship behaviors and display strong sexual dimorphism, with males exhibiting elaborate feather ornaments that serve as secondary sexual traits [3]. As such, strong sexual and natural selection have likely acted in concert to produce the exquisite phenotypic diversity among members the Paradisaeidae.

While having attracted substantial attention from systematists for centuries, the evolutionary processes and genomic mechanisms that have shaped these phenotypes remain largely unknown. In the past, the evolutionary history of birds-of-paradise has been studied with morphological data [1], molecular distances [4, 5], and a single mitochondrial gene [6], but the conclusions have been largely incongruent. The most comprehensive phylogenetic study at present includes all 41 species and is based on DNA-sequence data from both mitochondrial (cytochrome B) and nuclear genes (ornithine decarboxylase introns ODC6 and ODC7) [7]. This study suggested that the birds-of-paradise started to diverge during late Oligocene or early Miocene and could be divided into five main clades. The sexually monomorphic genera *Manucodia, Phonygammus*, and *Lycocorax* form a monophyletic clade (Clade A; Fig. 1 in Irestedt et al. [7]), which was suggested to be sister to the other four clades that include species, many of which show strong sexual dimorphism (here, referred to as “core birds-of-paradise”). Among the latter four clades, the genera *Pteridophora* and *Parotia* were suggested to form the earliest diverging clade (Clade B; Fig. 1 in Irestedt et al. [7]), followed by a clade consisting of the genera *Seleucidis, Drepanornis, Semioptera, Ptiloris*, and *Lophorina* (Clade C; Fig. 1 in Irestedt et al. [7]). The last two sister clades are comprised of *Epimachus, Paradigalla*, and *Astrapia* (Clade D; Fig. 1 in Irestedt et al. [7]), and *Diphyllodes, Cicinnurus*, and *Paradisaea* (Clade E; Fig. 1 in Irestedt et al. [7]), respectively. Overall, the phylogenetic hypothesis presented in Irestedt et al. [7] receives strong branch support (posterior probabilities), but several nodes are still weakly supported and there is incongruence among gene trees. Recently, Irestedt and colleagues [8] and Scholes and Laman [9] argued for the Superb birds-of-paradise to be split into several species, based on genetics, morphology, and courtship behavior. Thus, while preliminary genetic analyses have outlined the major phylogenetic divisions, the interspecific relationships of birds-of-paradise remain largely unresolved.

**Figure 1:**
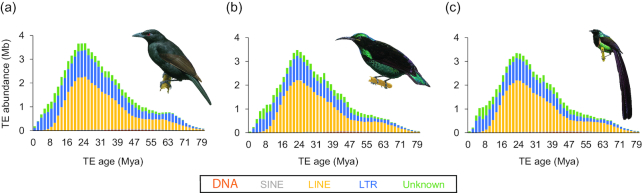
Repeat landscapes of *Lycocorax pyrrhopterus, Ptiloris paradisaeus*, and *Astrapia rothschildi*. Total amounts of transposable element (TE)-derived bp are plotted against relative age, approximated by per-copy Kimura 2-parameter distance to the TE consensus sequence and scaled using a four-fold degenerate mutation rate of Passeriformes of 3.175 substitutions/site/million years. TE families were grouped as “DNA transposons” (red), “SINEs” (short interspersed nuclear element; gray), “LINEs” (long interspersed nuclear element; yellow), “LTRs” (long terminal repeats; blue), and “Unknown” (green).

Birds-of-paradise are most widely known for their extravagant feather types, coloration, and mating behaviors [3]. In addition, they also exhibit an array of bill shapes (often specialized to their respective foraging behavior), body morphologies, and sizes [3]. Ornament feather types include “wire-type” tail feathers (e.g., 12-wired bird-of-paradise [*Seleucidis melanoleuca*]), erectile head plumes (e.g., king of Saxony bird-of-paradise [*Pteridophora alberti*]), significantly elongated tail feathers (e.g., ribbon-tailed Astrapia [*Astrapia mayeri*]) or finely filamental flank plumes (e.g., lesser bird-of-paradise [*Paradisaea minor*]; see Frith and Beehler [3]). Feathers and coloration are crucial components of their mating displays. Polygynous birds-of-paradise show aggregated leks high in tree tops, less aggregated leks on lower levels or the forest floor (often exploded leks), and even solitary mating displays [3].

Coloration in birds-of-paradise involves both pigment-based and structural mechanisms. Coloration via pigmentation is achieved by pigment absorption of diffusely scattered light in a specific wavelength range. Pigments such as carotenoids are frequently associated with red and yellow hues in birds, whereas light absorption by various classes of melanin pigments give rise to black plumage features common in many birds-of-paradise species [10]. On the other hand, structural coloration is caused by constructive interference and light reflection from quasiordered spongy structures of the feather barbs and melanosomes in feather barbules [11, 12]. The plumages of male birds-of-paradise feature both coloration types to various degrees, and some species such as the Lawe's parotia (*Parotia lawesii*) use angular-dependent spectral color shifts of their structural feathers in their elaborate display rituals to attract females [13, 14] Dale and colleagues recently showed that sexual selection on male ornamentation in birds has antagonistic effects, where male ornamentation is increasing, while females show a strong reduction in ornamentation [15]. This is very apparent in polygynous core birds-of-paradise, where females between species and sometimes even between genera look highly similar.

The array of extravagant phenotypes found in birds-of-paradise makes them an interesting model to study evolution. However, fresh tissue samples from birds-of-paradise are extremely limited, and currently only about 50% of all species are represented in biobanks. Fortunately, the current revolution in sequencing technologies and laboratory methods enables us to sequence whole-genome data from non-model organisms and it also allows us to harvest genome information from specimens in museum collections (by using sequencing library preparation methods specifically designed for degraded DNA in combination with high-throughput sequencing) [16]. Only recently have these technological advances enabled researchers to investigate genome-wide signals of evolution using comparative and population genomic approaches in birds [17–20].

In the current study, we made use of these technological advances to generate *de novo* genomes for three birds-of-paradise species from fresh samples and re-sequenced the genomes of two other species from museum samples. Using these genomes, we were able to contrast the trajectory of genome evolution across passerines and simultaneously evaluate which genomic features have evolved during the radiation of birds-of-paradise. We identified a set of candidate genes and genomic features that might have contributed to the extraordinary diversity in phenotypic traits found in birds-of-paradise.

## Results

### Assembly and gene annotation

We *de novo* assembled the genomes of *Lycocorax pyrrhopterus, Ptiloris paradiseus*, and *Astrapia rothschildi* using paired-end and mate pair Illumina sequence data and performed reference-based mapping for *Pteridophora alberti* and *Paradisaea rubra*. Scaffold N50 ranged from 4.2 Mb (*L. pyrrhopterus*) to 7.7 Mb (*A. rothschildi*), and the number of scaffolds ranged from 2,062 (*P. paradiseus*) to 3,216 (*L. pyrrhopterus*; Table 1). All assemblies showed a genome assembly size around 1 Gb (1.03 to 1.07 Gb, see Table 1). Benchmarking Universal Single-Copy Orthologs 2 (BUSCO2) [21] scores for complete genes (using the aves_odb9 database) found in the respective assemblies ranged from 93.8% to 95.1%, indicating a high completeness (Supplementary Table S1). Next, we annotated the genomes using homology to proteins of closely related species as well as *de novo* gene prediction. Gene numbers ranged from 16,260 (*A. rothschildi*) to 17,269 (*P. paradiseus;* see Supplementary Table S2).

**Table 1:** *De novo* assembly statistics

	Number of scaffolds	Scaffold N50, Mb	Assembly length, Gb
*Astrapia rothschildi*	2,081	7.7	1.03
*Lycocorax pyrrhopterus*	3,216	4.2	1.07
*Ptiloris paradiseus*	2,062	4.27	1.04

### Repeat evolution in birds-of-paradise

Our *de novo* repeat annotation analyses (Supplementary Table S3) suggest that the genomes of birds-of-paradise contain repeat densities (∼7%) and compositions (mostly chicken repeat 1 [CR1] long interspersed nuclear elements [LINEs], followed by retroviral long terminal repeats [LTRs]) well within the usual range of avian genomes [22]. However, we identified 16 novel LTR families (solo LTRs; Supplementary Table S4) with no sequence similarity to each other or to LTR families known from in-depth annotations of chicken (*Gallus gallus*), zebra finch (*Taeniopygia guttata*), and collared flycatcher (*Ficedula albicollis*) [23, 24]. Interestingly, we found that activity of CR1 LINEs ceased recently in the three birds-of-paradise and was replaced by activity of retroviral LTRs (Fig. 1). The inferred timing of the transposable element (TE) activity or accumulation peak (Fig. 1) roughly corresponds to or slightly predates the radiation of birds-of-paradise (inferred in Irestedt et al. [7]). We also found that the genome assembly of *Lycocorax pyrrhopterus* exhibits slightly higher repeat densities than those of the two other birds-of-paradise (Supplementary Table S3) and slightly more recent TE activity (Fig. 1). A possible explanation for this is that this is the only female bird-of-paradise assembly, thus containing the female-specific W chromosome which is highly repetitive [22].

### Genome synteny to the collared flycatcher

**Figure 2:**
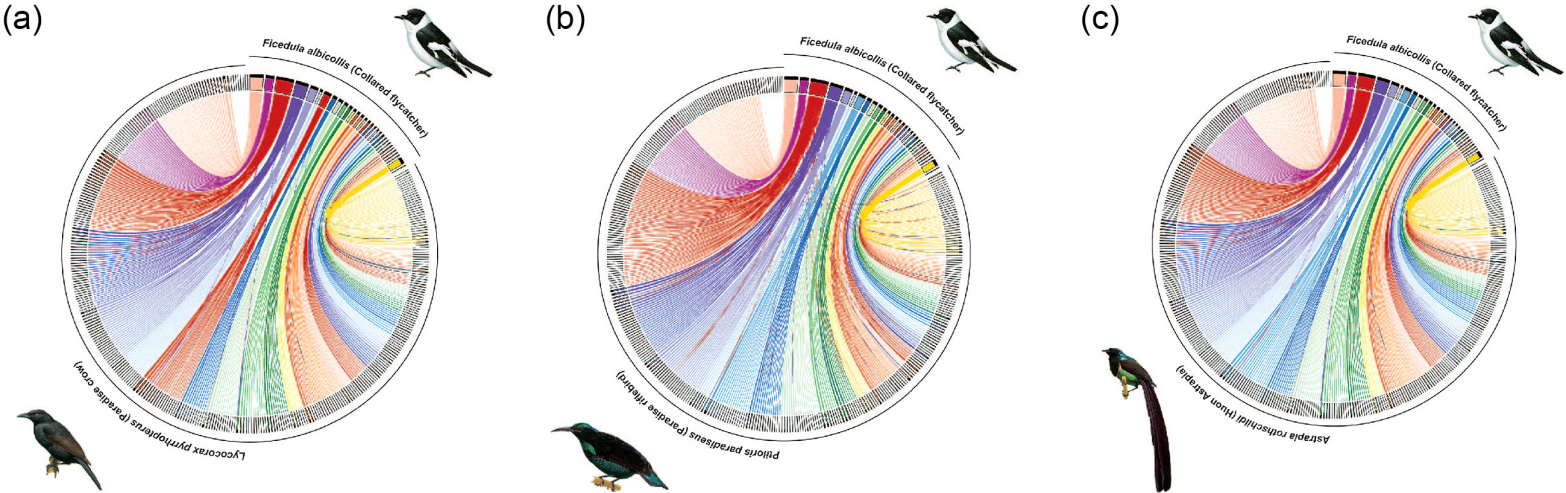
Chromosomal synteny plot between the collared flycatcher and **(a)** the paradise crow, **(b)** paradise riflebird, and **(c)** the Huon astrapia. The plot shows scaffolds larger than 50 kb and links (alignments) larger than 2 kb.

We found strong synteny of the three *de novo* assembled birds-of-paradise genomes (*Lycocorax pyrrhopterus, Ptiloris paradiseus, Astrapia rothschildi*) to that of the collared flycatcher (Fig. 2 and Supplementary Figs S1–S3). Only few cases were found where individual scaffolds of the birds-of-paradise genomes mapped to multiple chromosomes in the collared flycatcher genome.

### Phylogeny

**Figure 3:**
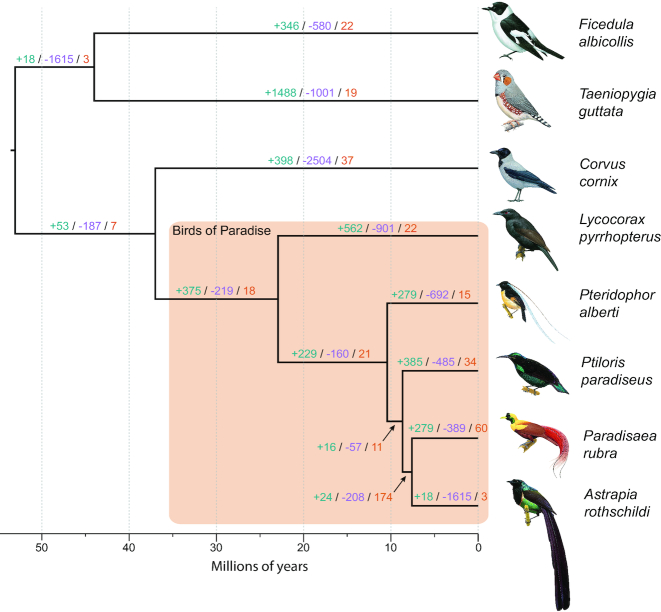
The birds-of-paradise phylogeny. The phylogenetic tree is based on Maximum Likelyhood and coalescent-based statistical binning of 4,656 genes and scaled using the divergence times between crow and the birds-of-paradise, and zebra finch and flycatcher (obtained from Timetree.org) as calibration points. Branches are labeled as: # gene family expansions/# gene family contractions/# rapidly evolving gene families.

We found 4,656 one-to-one orthologous genes to be present in all eight sampled bird genomes (five birds-of-paradise and three outgroup songbirds). A phylogeny inferred using these orthologs shows a topology with high bootstrap scores (Fig. 3 and Supplementary Fig. S4). However, the sole use of bootstrapping or Bayesian posterior probabilities in analyses of large-scale datasets has come into question in recent years [25]. Studies based on genome-wide data have shown that phylogenetic trees with full bootstrap or Bayesian posterior probability support can exhibit different topologies (e.g., Jarvis et al. [26] and Prum et al. [27]; discussed in Suh [25]). Thus, next we performed a concordance analysis by comparing gene trees for the 4,656 single-copy orthologs to the inferred species topology. We found strong concordance for the older splits in our phylogeny (see Supplementary Fig. S4). However, the splits between *Ptiloris* and its sister clade, which contains *Astrapia* and *Paradisaea*, and the split between *Astrapia* and *Paradisaea* itself showed much lower concordance values, 0.31 and 0.26, respectively. Only ∼10% of the gene trees exactly matched the topology of the inferred species tree, and we found an average Robinson-Foulds distance of 3.92 for all gene trees compared to the species tree (Supplementary Table S5). A Robinson-Foulds distance of 0 would indicate that the two tree topologies (species to gene tree) are identical. The highest supported species tree topology (Fig. 3 and Supplementary Fig. S4) is concordant to the birds-of-paradise species tree constructed in Irestedt et al. [7]. Overall, we found that the birds-of-paradise form a monophyletic clade, with the crow (*Corvus cornix*) being the most closely related sister taxon, in most gene trees (74%). Within the birds-of-paradise clade, we further distinguish a core birds-of-paradise clade, which consist of four of the five species in our sample (excluding only the paradise crow, *Lycocorax pyrrhopterus*).

### Positive selection in the birds-of-paradise

We carried out positive selection analyses using all previously ascertained orthologous genes (8,134 genes present in at least seven out of the eight species) on the branch leading to the birds-of-paradise. First, we investigated saturation by calculating pairwise dN/dS ratios. The inferred values did not show any signs of saturation (Supplementary Table S6). To infer positive selection on the branch of the birds-of-paradise, we used the BUSTED model in HyPhy (similar to branch-sites model; [28]). We found 213 genes to be under selection (*P* value < 0.05; gene symbol annotation for 212 of the 213 genes can be found in Supplementary Table S7). Multiple-testing correction was carried out using FDR in R (function *p.adjust()* from the stats package).

### Gene gain and loss

We identified 9,012 gene families across all eight species. Using CAFE [29], we inferred 98 rapidly evolving families within the birds-of-paradise clade. Supplementary Table S8 summarizes the gene family changes for all eight species (also see Fig. 3). Zebra finch had the highest average expansion rate across all families at 0.0916, while the hooded crow had the lowest average expansion rate at –0.1724, meaning that they have the most gene family contractions. Gene gain/loss rates can be found in Supplementary Table S9. Next, we tested for enrichment of Gene Ontology (GO) terms in the set of families rapidly evolving in the birds-of-paradise clade. Gene families were assigned GO terms based off the Ensembl GO predictions for flycatcher and zebra finch. In all, we were able to annotate 6,350 gene families with at least one GO term. Using a Fisher exact test on the set of 98 rapidly evolving families in the birds-of-paradise, we found 25 enriched GO terms in 20 families (FDR 0.05; Supplementary Table S10). All the gene gain and loss results can be found online [30].

## Discussion

Renowned for their extravagant plumage and elaborate courtship displays, the birds-of-paradise are among the most prominent examples of how sexual selection can give rise to extreme phenotypic diversity. Despite extensive work on systematics and a long-standing interest in the evolution of their different mating behaviors, the genomic changes that underlie this phenotypic radiation have received little attention. Here, we have assembled representative genomes for the five main birds-of-paradise clades and characterized differences in genome evolution within the family and relative to other avian groups. We reconstructed the main structure of the family phylogeny, inferred the TE landscape, and identified a list of genes under selection and gene families significantly expanded or contracted potentially involved in many phenotypic traits for which birds-of-paradise are renowned. Below, we discuss these different genomic features and how they might have contributed to the evolution of birds-of-paradise.

### Genome synteny and phylogeny

We found genome synteny (here in comparison to the collared flycatcher [31]) to be highly conserved for all three *de novo* assembled genomes (Fig. 2 and Supplementary Figs S1–S3). Only a few cases were recorded where regions of scaffolds of the birds-of-paradise genomes aligned to different chromosomes of the collared flycatcher. These could be artifacts of the genome assembly process or be caused by translocations. Passerine birds show variable numbers of chromosomes (72–84 [22]). However, they do not vary as much as other groups’, such as Charadriiformes (shorebirds, 40–100 [22]). In general, studies have shown a high degree of genome synteny even between Galloanseres (galliform and anseriform birds) and Neoaves (most other birds including passerines; approximately 80–90 million years ago (Mya) divergence; reviewed in Ellegren [32] and Poelstra et al. [17]). However, genomes with higher continuity, generated with long-read technologies or using long-range scaffolding methods (such as Hi-C [33]), or a combination thereof, will be needed to get a more detailed view of rearrangements in birds-of-paradise genomes (see Peona et al. [34]).

Our analyses reconstructed a phylogenetic tree topology congruent with the one presented in Irestedt et al. [7] (Fig. 3 and Supplementary Fig. S4). However, while bootstrapping found strong support for the topology of the species tree, congruence analysis found high discordance for the two most recent branches (*Ptiloris* and its sister clade [*Astrapia* and *Paradisaea*] and the split between *Astrapia* and *Paradisaea*). Furthermore, we found the highest supported tree topology to be based on only 10% of all gene trees (Supplementary Table S5). This could be caused by incomplete lineage sorting (ILS), which refers to the persistence of ancestral polymorphisms across multiple speciation events [35]. Jarvis et al. [26] and Suh et al. [36] showed that ILS is a common phenomenon on short branches in the avian Tree of Life. Another possibility could be hybridization, a phenomenon frequently recorded in birds-of-paradise [3]. Overall, most gene tree topologies (74%) support the monophyly for the birds-of-paradise and the core birds-of-paradise clades.

### Repeats and their possible role in the evolution of birds-of-paradise

Bursts of TE activity are often lineage or species specific, which highlights their potential to affect speciation [31]. This is further supported by the fact that TE activity bursts often correlate with the speciation timing of the respective species or species group [31]. Similarly, we found a burst of LINE activity within all three *de novo* assembled genomes (*Lycocorax pyrrhopterus, Ptiloris paradiseus, Astrapia rothschildi*) dating back to about 24 Mya (Fig. 1). The timing roughly corresponds to or slightly predates the emergence and radiation of birds-of-paradise (see Fig. 3). The notion that we found 16 novel families of retroviral LTRs suggests multiple recent germline invasions of the birds-of-paradise lineage by retroviruses. The recent cessation of activity of CR1 LINEs and instead recent activity of retroviral LTRs (Fig. 1) is in line with similar trends in collared flycatcher and hooded crow [22, 24]. This suggests that recent activity of retroviral LTRs might be a general genomic feature of songbirds, however, with different families of retroviruses being present and active in each songbird lineage. Thus, an intriguing hypothesis that warrants further investigation is that the diversification of birds-of-paradise (and probably songbirds in general) was influenced by lineage specificity of their TE repertoires through recurrent retroviral germline invasions and smaller activity bursts.

### Coloration and feather and skeletal development in birds-of-paradise

Given the strong sexual dimorphism and array of morphological phenotypes found in birds-of-paradise and their important role in mating success, we would expect genes important for coloration, morphology, and feather structure to be under selection in the birds-of-paradise. Indeed, we found several genes potentially involved in these phenotypic traits to show signatures of positive selection. However, most of them were non-significant after the multiple-testing correction (see Supplementary Table S7). Given that these corrections are often very conservative, these genes might still warrant more detailed follow-up investigations. One such gene is ADAMTS20, which is crucial for melanocyte development. ADAMTS20 has been shown to cause white belt formation in the lumbar region of mice [37]. Nonsense or missense mutations in this gene disrupt the function of KIT, a protein that regulates pigment cell development [37]. In mammals and birds, pigment patterns are largely influenced by melanocytes. Thus, this gene could be a strong candidate for differential coloration in the birds-of-paradise. Another gene under positive selection with a potential role in coloration is ATP7B. It is a copper-transporting P-type ATPase and thought to translate into a melanosomal protein (see Bennett and Lamoreux [38] for a review). Copper is crucial for melanin synthesis because tyrosinase contains copper and thus ATP7B might play a crucial role in pigment formation. Candidate genes for positive selection, which are known to have functions in feather development (in chicken) and morphology (in chicken and mammals) include FGR1, SPECC1L, BMPR1A, GAB2, PAPSS2, DCST2, ALDH3A2, MYF5, and APOBEC2. For example, FGFR1 (fibroblast growth factor receptor 1) is implicated in feather development [39]. In humans it has further been shown to be involved in several diseases associated with craniofacial defects (OMIM; [40]). ALDH3A2 (aldehyde dehydrogenase 3 family member A2), a membrane-associated protein, and SPECC1L are implicated in craniofacial disorders (e.g., Van Der Woude syndrome) in humans [41]. APOBEC2 seems to play a role in muscle development (skeletal and heart muscle) in chickens [42].

### APOBECs and their potential role in the immune system

Intriguingly, APOBECs have also been shown to have important functions in the immune systems of vertebrates, where they act as restriction factors in the defense against a range of retroviruses and retrotransposons [43, 44]. Functioning as cytosine deaminases, they act against endogenous retroviruses, especially LTR retrotransposons by interfering with the reverse transcription and by hypermutating retrotransposon DNA. A recent study on 123 vertebrates showed that birds have the strongest hypermutation signals, especially oscine passerines [45] (such as zebra finch and medium ground finch). That study also demonstrated that edited retrotransposons may preferentially be retained in active regions of the genome, such as exons and promoters, because hypermutation decreases their potential for mobility. Thus, it seems very likely that retrotransposon editing via APOBECs has an important role in the innate immunity of vertebrates as well as in genome evolution. In line with this hypothesis, we found a burst in recent activity of retroviral LTRs in the genomes of birds-of-paradise, a signal also found in other passerines [22, 24]. This could also explain why we found APOBEC2 to be under positive selection.

### Sensory system in the birds-of-paradise

#### Visual system

Genes that showed positive selection signals and are known to have known roles in eye function and development include CABP4, NR2E3, IMPG1, AKAP13, MGARP, GNB1, ATP6AP2, and MYOC, of which the latter three remained significant after multiple-testing correction. Interestingly, there are no obvious explanations for selection on vision in birds-of-paradise. Evidence for co-evolution between coloration and vision in birds is weak (see, e.g., Lind et al. [46], Price [47], but see Mundy et al. [48] and Bloch [49]). A phenotype that could be associated with selection on vision is the diverse array of mating displays in some core birds-of-paradise. Many species, such as Lawes's parotia (*Parotia lawesii*), modify color by changing the angle of the light reflection [13, 14], which requires the visual system to be able to process the fine nuances of these color changes. However, the fact that (color) vision serves many purposes (including, e.g., foraging) makes it very difficult to establish co-evolution between coloration and color vision [46]. We can thus only speculate at this point about the potential role of coloration or mating displays in the selection of vision genes found in birds-of-paradise.

#### Olfactory system

Another often overlooked sensory system in birds is odor perception. Olfactory receptors are important in odor perception and detection of chemical cues. In many animal taxa, including birds, it has been shown that olfaction is crucial to identify species [50], relatedness [51], individuals [52], as well as for mate choice [53] and in foraging [54]. In concordance with previous studies, we found this gene family to expand rapidly in the zebra finch [55]. Even more so, the zebra finch showed the strongest expansion (+17 genes) in our dataset. Furthermore, we found a rapid expansion on the branch leading to the core birds-of-paradise (+5 genes) and further in *Astrapia* (+6 genes). Interestingly, olfactory receptor genes show rapid contractions in the paradise crow (-6 genes), the hooded crow (–9 genes), and the collared flycatcher (–5 genes). This is in line with a study that suggested poor olfactory development in a different corvid species, the Japanese jungle crow (*Corvus macrorhynchos*) [56]. Olfactory could serve many functions in birds-of-paradise such as species recognition (to avoid extensive hybridization), individual recognition, mating, or foraging (given their extensive diet breadth).

#### Startle response and adult locomotory behavior

Startle response is an important behavioral trait. It is the ability to react quickly to the presence of a stimulus. We found a gene family associated with startle response and adult locomotory behavior to be evolving significantly faster than under a neutral model on the branch leading the core birds-of-paradise (+5 genes). It is even further expanded in *Paradisaea* (+3 genes). This gene family is contracted in the two outgroups, the zebra finch (–4 genes) and the collared flycatcher (–2 genes), as well as the monochromatic, non-lekking paradise crow (–1 genes). We found no expansion or contraction in the hooded crow genome. For core birds-of-paradise that show extravagant lekking behavior, fast response to stimuli or increased locomotory behavior could have several advantages. For example, being highly visible during leks means that lekking birds need to be able to look out for predators and react to them quickly. Indeed, Frith and Beehler [3] mention that lekking birds-of-paradise appear to be constantly on the lookout for predators. Interestingly, species of the genus *Paradisaea* have aggregated leks high in emergent trees and thus may be more visible to the numerous birds of prey that inhabit the region [3]. It could further be important for interaction between males or between the sexes during leks.

## Conclusions

We found indications of a conserved synteny between birds-of-paradise and other passerine birds, such as the collared flycatcher. Similar to other passerine genomes, we also found signatures of recent activity of novel retroviral LTRs in the genomes. Furthermore, several genes with known function in coloration, feather, and skeletal development showed signatures of positive selection in birds-of-paradise. This is in accordance with our prediction that phenotypic evolution in birds-of-paradise should have left genomic signatures. While these genes all are obvious candidates for evolution of birds-of-paradise's phenotypic and behavioral diversity, we also found positively selected genes that are not as straightforward to explain. These include genes involved in development and function of the visual system. Gene gain/loss analyses further revealed significant expansions in gene families associated with “startle response/locomotory behavior” and olfactory function.

Although the efforts to document the phenotypic and behavioral diversity in this model system of sexual selection continues to generate intense interest in birds-of-paradise, we still have limited understanding of the processes that have shaped their evolution. Here, we provide a first glimpse into genomic features underlying the diverse array of species found in birds-of-paradise. However, our analyses concentrate on comparative genomics, and we acknowledge that analyses based on multiple individuals per species will likely result in more reliable inferences. Furthermore, some of the most interesting traits in birds-of-paradise, such as lekking behavior, are likely complex traits and will require analyses based on many individuals (such as Quantitative Trait Loci mapping). In general, more in-depth analyses will be needed to establish a causal relationship between signatures of selection in the birds-of-paradise genome and the unique diversity of phenotypic traits or to investigate changes in genome structure with higher resolution. Fortunately, technologies keep advancing, and along with decreasing costs for sequencing, we will soon be able to gain more information about this fascinating but genetically understudied family of birds.

## Data Description and Analyses

### Sampling and DNA extraction

For the three *de novo* genome assemblies, *Lycocorax pyrrhopterus* (ZMUC149607; collected 2013, Obi Island, Indonesia), *Ptiloris paradiseus* (ANWC43271; collected 1990, New South Wales, Australia), and *Astrapia rothschildi* (KU Birds 93602; collected 2001, Morobe Province, Papua New Guinea), DNA was extracted from fresh tissue samples using the Qiagen QIAamp DNA Mini Kit according to the manufacturer's instructions. The *de novo* libraries with different insert sizes (see below) were prepared by Science for Life Laboratory, Stockholm. For the two re-sequenced genomes, *Pteridophora alberti* (NRM571458; collected 1951, Eastern Range, New Guinea) and *Paradisaea rubra* (NRM700233; collected 1949, Batanta Island, New Guinea), we sampled footpads and extracted DNA using the Qiagen QIAamp DNA Micro Kit according to the manufacturer's instructions. We applied precautions for working with museum samples described in Irestedt et al. [57]. Sequencing libraries for these two samples were prepared using the protocol published by Meyer and Kircher [58]. This method was specifically developed to generate sequencing libraries for low input DNA, showing DNA damage typical for museum or ancient samples.

### Genome sequencing, assembly, and quality assessment

We prepared two paired-end (overlapping and 450 bp average insert size) and two mate pair libraries (3 kb and 8 kb average insert size) for each of the three *de novo* assemblies (*Ptiloris paradiseus, Astrapia rothschildi*, and *Lycocorax pyrrhopterus*). All libraries, for the *de novo* and the reference-based mapping approaches were sequenced on a HiSeq2500 v4 at SciLifeLab Stockholm, Sweden. We generated two lanes of sequencing for each *de novo* assembly and pooled the two reference-based samples on one lane. We first assessed the read qualities for all species using the program FastQC (FastQC, RRID:SCR_014583) [59]. For the three species, *Ptiloris paradiseus, Astrapia rothschildi*, and *Lycocorax pyrrhopterus* we then used the *preqc* [60] function of the SGA [61] assembler to (1) estimate the predicted genome size, (2) find the predicted correlation between *k*-mer sizes and N50 contig length, and (3) assess different error statistics implemented in *preqc*. For *Ptiloris paradiseus, Astrapia rothschildi*, and *Lycocorax pyrrhopterus*, reads were assembled using Allpaths-LG [62]. To improve the assemblies, especially in repeat regions, GapCloser (GapCloser, RRID:SCR_015026; part of the SOAPdenovo package [63]) was used to fill in gaps in the assembly. Assemblies were then compared using CEGMA (CEGMA, RRID:SCR_015055) [64] and BUSCO2 (BUSCO (RRID:SCR_015008)) [21]. We added BUSCO2 scores for better comparisons at a later stage of the project. For the reference-based mapping, we mapped all reads back to the *Ptiloris paradiseus* assembly using BWA (BWA, RRID:SCR_010910) [65] (mem option), the resulting sam file was then processed using samtools [66]. To do so, we first converted the sam file generated by BWA to the bam format, then sorted and indexed the file. Next, we removed duplicates using Picard (Picard, RRID:SCR_006525) [67] and realigned reads around indels using GATK (GATK, RRID:SCR_001876) [68]. The consensus sequence for each of the two genomes was then called using ANGSD [69] (using the option -doFasta 3).

### Repeat annotation

We predicted lineage-specific repetitive elements *de novo* in each of the three birds-of-paradise genome assemblies using RepeatModeler (RepeatModeler, RRID:SCR_015027) v. 1.0.8 [70]. RepeatModeler constructs consensus sequences of repeats via the three complementary programs RECON [71], RepeatScout (RepeatScout, RRID:SCR_014653) [72], and Tandem Repeats Finder [73]. Next, we merged the resultant libraries with existing avian repeat consensus sequences from Repbase [74] (mostly from chicken and zebra finch) and recent in-depth repeat annotations of collared flycatcher [24, 75] and hooded crow [76]. Redundancies among the three birds-of-paradise libraries and between these and existing avian repeats were removed using the ReannTE_mergeFasta.pl script [77]. For *Lycocorax pyrrhopterus* repeats, we manually inspected the RepeatModeler library of consensus sequences for reasons reviewed in Platt et al. [78] and because *Lycocorax pyrrhopterus* was the most repeat-rich genome among the three birds-of-paradise. Manual curation was performed using standard procedures [36, 79], namely, screening of each repeat candidate against the *Lycocorax pyrrhopterus* assembly using blastn [80], extracting the 20 best hits including 2-kb flanks, and alignment of these per-candidate Basic Local Alignment Search Tool (BLAST) hits to the respective consensus sequence using MAFFT (MAFFT, RRID:SCR_011811) v. 6 [81]. Each alignment was inspected by eye, and curated majority-rule consensus sequences were generated manually considering repeat boundaries and target site duplication motifs. This led to the identification of 33 LTR retrotransposon consensus sequences (including 16 novel LTR families named as “lycPyrLTR*”) and three unclassified repeat consensus sequences (Supplementary Table S4). We then used this manually curated repeat library of *Lycocorax pyrrhopterus* to update the aforementioned merged library of avian and birds-of-paradise repeat consensus sequences. Subsequently, all three birds-of-paradise genome assemblies were annotated via RepeatMasker (RepeatMasker, RRID:SCR_012954) v. 4.0.6 and “-e ncbi” [82] using this specific library (Supplementary Table S3). Landscapes of relative TE activity (i.e., the amount of TE-derived bp plotted against Kimura 2-parameter distance to respective TE consensus) were generated using the calcDivergenceFromAlign.pl and createRepeatLandscape.pl scripts of the RepeatMasker package. To enhance plot readability, TE families were grouped into the subclasses “DNA transposon,” “SINE,” “LINE,” “LTR,” and “Unknown” (Fig. 1). We scaled the Kimura substitution level with the four-fold degenerate mutation rate for Passeriformes (mean of 3.175 substitutions/site/million years for passerines sampled in Zhang et al. [19]) to obtain an estimate of the timing of the inferred repeat activity in million years ago.

### Genome synteny

We also inferred genome architecture changes (synteny) between our three *de novo* assembled genomes and the chromosome-level assembly of the collared flycatcher [31]. To do so, we first performed pairwise alignments using Satsuma [83] and then plotted the synteny using Circos plots [84]. More precisely, we first performed asynchronous “battleship”-like local alignments using *SatsumaSynteny* to allow for time-efficient pairwise alignments of the entire genomes. In order to avoid signals from repetitive elements, we used masked assemblies for the alignments. Synteny between genomes was then plotted using Circos and in-house perl scripts.

### Gene annotation

We masked repeats (only transposable elements) in the genome prior to gene annotation. Contrary to the repeat annotation step, we did not mask simple repeats in this approach. Those were later soft-masked as part of the gene annotation pipeline Maker2 [85], to allow for more efficient mapping during gene annotation.

Gene annotation was performed using *ab initio* gene prediction and homology-based gene annotation. To do so, we used the genome annotation pipeline Maker2 [85], which is able to perform all the aforementioned genome annotation strategies. Previously published protein evidence (genome annotations) from Zhang et al. [19] was used for the homology-based gene prediction. To improve the genome annotation, we used CEGMA to train the *ab initio* gene predictor SNAP [86] before running Maker2 [85]. We did not train the *de novo* gene predictor Augustus [87] because no training dataset for birds was available and it was not recommended at the time to use potentially lower-quality annotations for training of Augustus (M. Stanke, personal communication).

### Ortholog gene calling

In the next step, we inferred orthologous genes using PoFF [88]. We included all five birds-of-paradise, as well as the hooded crow (*Corvus cornix)* [17], the zebra finch (*Taeniopygia guttata*) [89], and the collared flycatcher (*Ficedula albicollis*) [31] as outgroups. We ran PoFF using both the transcript files (in fasta format) and the transcript coordinates file (in gff3 format). The gff files were used (flag *–synteny*) to calculate the distances between paralogous genes to accurately distinguish between orthologous and paralogous genes. We then extracted the sequences for all one-to-one orthologs using a custom python script.

Next, we determined the number of genes with missing data in order to maximize the number of genes included in the subsequent analyses. For a gene to be included in our analyses, it had to be present in at least 75% of all species (six out of eight species), which resulted in a set of 8,134 genes. In order to minimize false positives in the subsequent positive selection analysis caused by alignment errors, we used the codon-based alignment algorithm of Prank [90] and further masked sites with possible alignment issues using Aliscore [91]. Aliscore uses Monte Carlo resampling within sliding windows to identify low-quality alignments in amino acid alignments (converted by the program). The identified potential alignment issues were then removed from the nucleotide alignments using ALICUT [92].

### Intron calling

In addition to exons, we also extracted intron information for the birds-of-paradise genomes (see Supplementary Table S2). To do so, we used the extract_intron_gff3_from_gff3.py script [93] to include intron coordinates into the gff file. We then parsed out all intron coordinates and extracted the intron sequences from the genomes using the exttract_seq_from_gff3.pl script [94]. All introns for the same gene were then concatenated using a custom python script.

### Phylogenetic analysis

The individual alignment files (we used exon sets without missing species, which resulted in 4,656 alignments) were then (1) converted to the phylip format individually and (2) 200 randomly selected exon alignments were concatenated and then converted to phylip format using the catfasta2phyml.pl script [95]. We used the individual exon phylip files (all exons combined per gene) for gene tree reconstruction using RaxML [96] (using the GTR + G model). Subsequently, we combined the gene trees into a species tree using a multi-species coalescent model and carried out bootstrapping using Astral [97, 98]. Because Astral does not provide branch lengths needed for calibrating phylogenetic trees (used for the gene gain/loss analysis), we subsampled our data and constructed a Maximum Likelihood (ML) tree based on 200 randomly chosen and concatenated exons using ExaML [99]. We then calibrated the species tree using the obtained branch lengths along with calibration points obtained from timetree.org using r8s [100]. These calibration points are the estimated 44 Mya divergence time between flycatcher and zebra finch and the 37 Mya divergence time between crow and the birds-of-paradise.

Next, we performed a concordance analysis. First, we rooted the gene trees based on the outgroup (collared flycatcher, zebra finch). Then, for each node in the species tree, we counted the number of gene trees that contained that node and divided that by the total number of gene trees. We next counted the number of gene trees that support a given topology (see Supplementary Table S65) and further calculated the Robison-Foulds distance between gene trees using RaxML.

### Inference of positive selection

We inferred genes under positive selection using *dN/dS* ratios of 8,133 orthologs. First, we investigated saturation of synonymous sites in the phylogenetic sampling using pairwise comparisons in CodeML [101]. The pairwise runmode of CodeML estimates *dN* and *dS* ratios using a ML approach between each species pair (Supplementary Table S6). We then investigated positive selection on the branch to the birds-of-paradise using the BUSTED model [28] (branch-site model) implemented in HyPhy [102]. The branch-site test allows for inference of positive selection in specific branches (foreground branches) compared to the rest of the phylogeny (background branches). The significance of the model comparisons was determined using likelihood-ratio tests. We carried out multiple testing corrections using FDR [103] instead of Bonferroni correction, as the branch-site tests result in an excess of non-significant *P* values, which violates the assumption of a uniform distribution in multiple testing correction methods such as the Bonferroni correction. The genes were then assigned gene symbols. To do so, we first extracted all the respective zebra finch or collared flycatcher GeneBank protein accessions. We then converted the accessions into gene symbols using the online conversion tool bioDBnet [104]. GO terms were obtained for the flycatcher assembly and assigned to orthologs that had a corresponding flycatcher transcript ID in Ensembl (7,305 genes out of 8,133). To determine enriched GO categories in positively selected genes, GO terms in genes inferred to have undergone positive selection were then compared to GO terms in all genes (with a GO term) using Fisher exact test with a false discovery rate cutoff of 0.05. We found 262 GO terms enriched in positively selected genes before FDR correction and 47 enriched after.

### Gene gain-loss

In order to identify rapidly evolving gene families in the birds-of-paradise, we used the peptide annotations from all five birds-of-paradise species, along with the three outgroup species in our analysis: hooded crow, zebra finch, and collared flycatcher. The crow genes were obtained from NCBI and the zebra finch and flycatcher genes were acquired from ENSEMBL 86 [105]. To ensure that each gene was counted only once, we used only the longest isoform of each protein in each species. We then performed an all-vs-all BLAST [80] search on these filtered sequences. The resulting e-values from the search were used as the main clustering criterion for the MCL program to group peptides into gene families [106]. This resulted in 13,289 clusters. We then removed all clusters only present in a single species, resulting in 9,012 gene families. Since CAFE requires an ultrametric time tree as input, we used r8s to smooth the phylogenetic tree with calibration points based on the divergence time of crow and the birds-of-paradise at 37 Mya and of flycatcher and zebra finch at 44 Mya [107].

With the gene family data and ultrametric phylogeny (Fig. 3) as input, we estimated gene gain and loss rates (*λ*) with CAFE v3.0 [108]. This version of CAFE is able to estimate the amount of assembly and annotation error (*ε*) present in the input data using a distribution across the observed gene family counts and a pseudo-likelihood search. CAFE is then able to correct for this error and obtain a more accurate estimate of *λ*. We found an *ε* of about 0.01, which implies that 3% of gene families have observed counts that are not equal to their true counts. After correcting for this error rate, we found *λ* = 0.0021. This value for *λ* is considerably higher than those reported for other distantly related groups (Supplementary Table S9). GO terms were assigned to genes within families based on flycatcher and zebra finch gene IDs from Ensembl. We used these GO assignments to determine molecular functions that may be enriched in gene families that are rapidly evolving along the ancestral BOP lineage (Node BOP11 in Supplementary Fig. S1). GO terms in genes in families that are rapidly evolving along the BOP lineage were compared to all other GO terms using a Fisher exact test (FDR cutoff of 0.05). We found 36 genes in 26 families to have enriched GO terms before FDR correction and 25 genes in 20 families after.

## Availability of supporting data

All genomes and supporting data are available via the *GigaScience* repository, GigaDB [109], and raw read data via the NCBI Sequence Read Archive; BioProject: PRJNA506819, BioSample: SAMN10474331-SAMN10474335, SRA Study: SRP173170). All results of the gene gain-loss analyses can be found online [30].

## Additional files

Supplementary Figure S1: Chromosomal synteny plot between the collared flycatcher and the paradise crow. The plot shows scaffolds larger than 50 kb and links (alignments) larger than 2 kb.

Supplementary Figure S2: Chromosomal synteny plot between the collared flycatcher and the paradise riflebird. The plot shows scaffolds larger than 50 kb and links (alignments) larger than 2 kb.

Supplementary Figure S3: Chromosomal synteny plot between the collared flycatcher and the Huon Astrapia. The plot shows scaffolds larger than 50 kb and links (alignments) larger than 2 kb.

Supplementary Figure S4: Phylogenetic species tree. The species tree was reconstructed from individual maximum likelihood-based gene trees using 4656 exons and coalescent-based statistical binning (Astral). Branch lengths are depicted on the branches (calculated via a ML tree constructed using ExaML and 200 randomly selected genes). Nodes are labeled and concordance factor is shown next to the node labels (i.e., [node label]/[concordance factor]). All nodes have 100 bootstrap support.

Supplementary Table S1: BUSCO scores. Scores were calculated using Busco2 and the aves_odb9 data set (4915 genes total).

Supplementary Table S2: Gene annotation.

Supplementary Table S3: RepeatMasker annotation of the three birds-of-paradise genome assemblies using a library of our *de novo* repeat annotations of birds-of-paradise merged with existing avian repeat libraries.

Supplementary Table S4: Characteristics of the manually curated TE consensus sequences from *Lycocorax pyrrhopterus*, including lineage-specific LTR families termed as “lycPyrLTR*”.

Supplementary Table S5: Top 10 gene tree topology counts (423 total topologies in 4450 rooted gene trees). Average Robinson-Foulds distance for all 4656 gene trees is 3.92. Z: zebra finch; F: collared flycatcher; C: hooded crow; L: *Lycocorax*; Pte: *Pteridophora*; Pti: *Ptiloris*; Par: *Paradisaea*; A: *Astrapia*.

Supplementary Table S6: Saturation Analysis. Pairwise dN/dS ratio.

Supplementary Table S7. Genes under positive selection. Gene symbols in bold mark genes significant after multiple-testing correction using FDR (<0.05 cut-off).

Supplementary Table S8: Summary of gene gain and loss events inferred after correcting for annotation and assembly error across all 13 species. The number of rapidly evolving families is shown in parentheses for each type of change.

Supplementary Table S9: Assembly/Annotation error estimation and gene gain/loss rates in a single *λ* model in the 13 mammals included in this study compared to previous studies using fewer species. * Dataset from Han et al. 2013 [1].

Supplementary Table S10: Enriched GO terms in rapidly evolving birds-of-paradise families. The number in parentheses for rapidly evolving lineages indicates the extent of change along that lineage (e.g., *Astrapia* (+6) means that the *Astrapia* lineage gained 6 genes). Lineages within the BOP clade are indicated by bold text. See Figure S1 for internal node labels.

GIGA-D-18-00163_Original_Submission.pdf

GIGA-D-18-00163_Revision_1.pdf

Reviewer_1_Report_Original_Submission -- Scott Taylor 5/31/2018 Reviewed

Reviewer_1_Report_Revision_1 -- Scott Taylor 11/11/2018 Reviewed

Reviewer_2_Report_Original_Submission -- Dominic Wright 6/7/2018 Reviewed

Reviewer_2_Report_Revision_1 -- Dominic Wright 11/22/2018 Reviewed

Reviewer_3_Report_Original_Submission -- Jelmer Poelstra 6/11/2018 Reviewed

Supplemental File

## Competing Interest

The authors declare that there is no competing interest.

## Authors Contribution

S.P., L.D., P.E., and M.I. conceived the project. E.P. and M.I. carried out the laboratory work. S.P., E.E.A., J.N., G.W.C.T. and A.S. B.P. carried out the data analyses. B.W.B. provided samples. S.P., E.E.A., J.N., G.W.C.T, A.S., B.P. , M.P.K.B., B.W.B., E. P. P.E., and M.I. wrote and revised the paper.

## Abbreviations

BLAST: Basic Local Alignment Search Tool; BUSCO: Benchmarking Universal Single-Copy Orthologs; CR1: chicken repeat 1; GO: Gene Ontology; ILS: incomplete lineage sorting; LINE: long interspersed nuclear elements; LTR: long terminal repeat; ML:Maximum Likelihood; Mya: million years ago; TE: transposable element.

## Funding

M.I. and P.E. were supported by the Swedish Research Council (grant 621-2013-5161 to P.E. and grant 621-2014-5113 to M.I.). We also acknowledge support from the Science for Life Laboratory, the National Genomics Infrastructure, Uppmax, and the EvoLab at the University of California Berkeley for providing resources for massive parallel sequencing and computational infrastructure. The funders had no role in study design, data collection and analysis, decision to publish, or preparation of the manuscript.
