# Supplementary material for: Comparative analyses identify genomic features potentially involved in the evolution of birds-of-paradise
Source: Gigascience. 2019 Jan 24;8(5):giz003. doi: 10.1093/gigascience/giz003 (PMC6497032; doi:10.1093/gigascience/giz003)
Supplement: GIGA-D-18-00163_Revision_1.pdf [file giz003_giga-d-18-00163_revision_1.pdf]

GigaScience

Comparative analyses identify genomic features potentially involved in the evolution of birds-of-paradise.

--Manuscript Draft--

**Manuscript Number:**	GIGA-D-18-00163R1
**Full Title:**	Comparative analyses identify genomic features potentially involved in the evolution of birds-of-paradise.
**Article Type:**	Research
**Funding Information:**	
**Abstract:**	The diverse array of phenotypes and courtship displays exhibited by birds-of-paradise have long fascinated scientists and laymen alike. Remarkably, almost nothing is known about the ge-nomics of this iconic radiation. There are 41 species in 16 genera currently recognized within the birds-of-paradise family (Paradisaeidae), most of which are endemic to the island of New Guinea. In this study, we sequenced genomes of representatives from all five major clades with-in this family to characterize genomic changes that may have played a role in the evolution of the group's extensive phenotypic diversity. We found genes important for coloration, morpholo-gy, feather and eye development to be under positive selection. In birds-of-paradise with com-plex lekking systems and strong sexual dimorphism, the core birds-of-paradise, we found GO categories for 'startle response' and 'olfactory receptor activity' to be enriched among the gene families expanding significantly faster compared to the other birds in our study. Furthermore, we found novel families of retrovirus-like retrotransposons active in all three de novo genomes since the early diversification of the birds-of-paradise group, which might have played a role in the evolution of this fascinating group of birds.
**Corresponding Author:**	Stefan Prost<br>Senckenberg<br>Frankfurt, GERMANY
**Corresponding Author Secondary Information:**	
**Corresponding Author's Institution:**	Senckenberg
**Corresponding Author's Secondary Institution:**	
**First Author:**	Stefan Prost
**First Author Secondary Information:**	
**Order of Authors:**	Stefan Prost
	Ellie E Armstrong
	Johan Nylander
	Gregg WC Thomas
	Alexander Suh
	Bent Petersen
	Love Dalen
	Brett W Benz
	Mozes PK Blom
	Eleftheria Palkopoulou
	Per GP Ericson
	Martin Irestedt

Powered by Editorial Manager® and ProduXion Manager® from Aries Systems Corporation

Order of Authors Secondary Information:	
Response to Reviewers:	Reviewer reports:<br><br>Reviewer #1: Prost et al. use whole genome sequence data from five bird-of-paradise species to explore patterns of evolution across the birds-of-paradise compared to other passerines, and identify a list of candidate genes that may have contributed to the diverse phenotypes seen within the radiation.<br><br>This paper is well-written and clear, but quite speculative and would perhaps be nicer as a shorter report with a reduced discussion. Realistically, an exploration of this nature will always feel speculative and like a bit of a gene list, and the authors acknowledge that the resources they are providing in this paper could set up some very interesting future studies. I agree with this. I think that the methods that the authors have used are sound, and that the genes they have identified are interesting new candidate genes to explore with respect to the incredible radiation that is the birds-of-paradise.<br><br>Regarding the discussion of TEs, I agree with the authors that the inclusion of a female in their study likely resulted in this higher repeat density in Lycocorax pyrrhopterus than the other species and I would caution the authors on inferring too much based on this result until they have comparable whole genome data from a male of this species.<br><br>We have toned down the interpretation of the TE results.<br><br>Other comments:<br><br>Page 2, Line 52 - As written "…more or less strong sexual dimorphism" is a little unclear. You're saying that, in general, these clades generally exhibit strong sexual dimorphism, right? I would reword for clarity.<br><br>We re-worded the sentence.<br><br>Page 7 Line 21 -Is there really no way to correct for multiple tests here? Seems to me these could all just be random given the sample size.<br><br>Even though, it is not ideal, as discussed in the paper, we have performed a FDR correction now.<br><br>Page 9 Line 19 - I would say pigment-based rather than pigmentary<br><br>Changed.<br><br>Page 10 Line 48 - 52 - how could positive selection not have played a role in morphological evolution? This seems like a throw away section.<br><br>Changed.<br><br>Page 11 Line 20 - 31 - the papers that have not found co-evolution between coloration and vision have examined opsin genes almost exclusively, not the genes found to be under positive selection in this paper. I agree that you're speculating here, but I think the connection between selection on vision genes and the displays and coloration of the birds-of-paradise is probably real.<br><br>We agree.<br><br>Page 12 Line 4 - I don't know that the authors should be so literal in their interpretation of the startle response results. Leks are more obvious, yes, and so predator evasion is important (What eats an adult bird of paradise?). Startle response is probably also important for interactions with other males in a lek and with visiting females. I would rather this part of the discussion was not just focused on the higher predation likelihood and more holistically discussed startle response in the context of bird-of-paradise lek behavior.<br><br>We now included a short discussion of other interactions.

Page 12 Line 22 - I think the "Other positively selected genes and enriched GO categories" section could be much reduced. Include the function of genes in the list of genes under positive selection rather than including that in the main text here.

We deleted this section, as we removed the GO enrichment from the analysis.

Page 17 Line 39 - excess, not access?

Changed.

Supplementary Table 3 - do intron size and number really not vary between the genomes?

We added that we rounded the values. They do vary, but only marginally. In general, bird genomes are highly conserved.

Reviewer #2: This is an interesting study that looks at the genomics of five different species of bird of paradise. These are extremely interesting, and under-studied, set of species (as the authors note), and the initial sequencing of these birds can certainly help to resolve the phylogeny surrounding them.

The study itself has two main aspects - firstly to resolve the phylogeny of these birds by sequencing three (and resequencing two) species, and secondly to attempt to identify the genes that underlie the range of different phenotypes that are exhibited by this set of species. The phylogeny resolution results and the conclusions drawn from them are sound and not over-interpreted. The major issues with this manuscript concern the search for positive selection and the use of GO terms to extrapolate the genes underlying the various phenotypes.

We removed the GO enrichment for the positive selection analysis, and carried out FDR correction.

Major Issues

1) In the search for positively selected genes the authors identify 178 genes using a nominal p-value significance if I understand their methods correctly, as they state the distribution of p-values gave an excess of non-significant p-values so therefore felt it was not appropriate to use a bonferonni or other multiple testing correction. Given that they test 8133 genes, some form of multiple testing correction is definitely required. Permutation testing seems the most obvious approach as that will be robust against non-normal distributions (see Nielsen, RCD et al, 2005, A scan for positively selected genes in the genomes of humans and chimpanzees. PloS Biology 3:723-733 for one example, but I am sure may others exist).

It was Rasmus Nielsen, first author of the mentioned paper, that told us not to perform any multiple-testing corrections in the first place. After further discussions, we decided to carry out a FDR correction, which should at least be less biased than methods like bonferroni. This is a heavily debated topic and it will be interesting to see if people will come up with better methods to test the reliability of positive selection signals.

2) In terms of using this technique (dN/dS ratios) to identify single genes that are being positively selected, this technique is more robust when all the genes in a genome are considered more to identify overall patterns (see Hartl and Clark 'Principles of Population Genetics'). By only having one representative per species as in this case, it should be mentioned that some of the variation present for that species may be being overlooked, which can bias the estimate accordingly.

We acknowledge that methods based on multiple individuals such as MKT, or QTL methods are more accurate and powerful. However, getting even one sample is a huge endeavor for some/most birds-of-paradise species and getting multiple samples will in many cases be almost impossible. In order to avoid some of the issues associated with using one individuals per species, we only performed positive selection tests on the

branch leading to the birds-of-paradise, as we do have at least five representatives for this branch.

3) The use of genes with some signature of positive selection as a means of determining genes underlying phenotypic traits is almost certainly over-extrapolated here. By taking a laundry list of genes and then picking potentially interesting putative functions and ascribing them to different traits without further proof is over-speculation (e.g. collagen and extracellular matrix genes are identified, but these genes can potentially affect a gamut of different functions, the authors then say that they are possibly affecting feather formation, craniofacial development, etc). As a further example, the authors state structural colours as being typified by these birds, and that some of these genes are potentially related to structural (and other) colouration. However, no actual genes for structural colours have been identified that I am aware of, so it is unlikely that any genes identified by previous means are the exact ones that control structural colour variation. In general the traits the authors state are of interest are complex quantitative traits, so the QTL controlling the inter-specific variation are most likely in non-coding regions, and depending on the linkage disequilibrium in the genomes of these birds, easily missed in any case when only considering coding regions. Therefore without additional analysis this speculation over gene function should be drastically reduced.

For reasons mentioned above, we had to use simple single-individual comparative genomics methods. We toned down the inferences based on the results and added a sentence on the issues related to comparative genomics methods. We further highlighted that some of the most interesting traits are most likely complex traits and will require more in depth analyses. In general, we only discussed genes which had known and tested functions to avoid over interpretation of the results.

4) Similarly, looking at GO enriched terms can also easily be extrapolated. For example, in a study by Pavlidis, P. et al. (A critical assessment of storytelling: GO categories and the importance of validating genomic scans. Mol Biol Evol 29(10):3237-3248, 2012), a set of selective sweeps were generated at random in a genome. In each instance, the genes were then analysed for GO enrichment and plausible functions ascribed in each case. This means that GO terms should be used as a complement to, rather than as the basis for the whole study.

The mentioned paper mostly shows that it is possible to get false positive selection signals from neutral data. However, we do acknowledge that GO enrichment has flaws and that there is no statistical framework to test any results, and so we removed it from the positive selection analysis.

In summary, the novel aspects of the sequencing of these genomes is very interesting, their use to resolve the phylogeny is strong, and they are an important step in being able to bring the full power of modern genomics to bear on these species. However, the use of just single representatives of each species to then attempt to prescribe putative phenotypic functions and relationships between the genes that are identified purely based on dN/dS ratios and then speculations regarding the genes identified is highly speculative and undermines the study as it currently is.

See above.

Reviewer #3: In this manuscript, Prost et al. perform whole-genome sequencing of five species of birds-of-paradise. Three of these are assembled de-novo, while the other two are mapped to one of the de-novo assembles genomes; all five are subsequently annotated. Special attention is paid to the evolution of repeats, while other analyses include phylogenetic relationships among the birds-of-paradise, the detection of positive selection and gene family size evolution. Genes inferred to have evolved under positive selection, as well as those being among rapidly evolving gene families, were next subjected to Gene Ontology tests, and a substantial part of the Discussion relates to specific genes and gene families whose evolution may have been relevant to that of birds-of-paradise.

I think the paper contains appropriate and solid analyses, and only have relatively

	minor comments. I do not find the results of the GO and related analyses particularly arresting and thought the Discussion was at parts quite dull while discussing gene after gene, but I realize the limitations of what one can do in genome papers like this one - I think that the authors overall strike a reasonable balance, and do not make unwarranted statements (except regarding TE evolution - see below). I would consider the overall writing somewhat subpar, as the paper contains relatively many errors and some sloppy, uncareful writing - some instances are pointed out in the specific comments below. Generally, I would recommend a careful rereading of the manuscript by the authors.<br><br>We shortened the discussion significantly and removed the GO enrichment analysis for the positive selection tests. Furthermore, we toned down the interpretation of the TE results.<br><br>Specific comments:<br><br>*I would suggest to make Table S1 into Table 1, i.e. to put it in the main part of the paper, and potentially to even combine it with Table S3 (and put the resulting table in the main part of the paper). The genome assembly and annotation is the backbone of this paper.<br><br>Done.<br><br>* p. 3, l. 30-34: "Fortunately, the current revolution in sequencing technologies and laboratory methods does not only enable us to sequence whole-genome data from non-model organisms, but it also allows us to harvest genome information from specimens in museum collections [10]." - This point, made in the Introduction, is not being returned to elsewhere in the paper, as far as I could see. I would appreciate it if the authors could make clear in Methods, Results and/or Discussion what it is, specifically, in recent technology/methods that facilitated the use of museum specimens in this manuscript, and perhaps also discuss the promise/limitations/future developments in this area.<br><br>We hope, we clarified this enough in the text.<br><br><br>* Discussion section "Repeats and their possible role in the evolution of birds-of-paradise" (page 6-7). I find this section quite speculative and insufficiently carefully worded.<br>- First, "A growing body of literature is emerging" - this sentence only cites a 9 year-old and a 10 year-old paper, which is not very convincing support of that statement.<br><br>We removed this sentence.<br><br>- Second, to see if the paper cited in the next sentence (ref. 30, which is from 2011) would provide an additional and more recent reference supporting the statement in the previous sentence, I had a look at it. It is cited as "Bursts of TE activity are often lineage and species-specific, which highlights their potential role in speciation", but the words "TE", "transposable", or even "repeat" or not to be found in this paper. Please clarify/change accordingly.<br><br>We cited the wrong paper by accident.<br><br>- "The timing [of a burst of TE activity] fits the emergence and radiation of birds-of-paradise". The fact that the emergence (ca. 35 ma) and radiation (I guess somewhere between 10 and 20-25 ma) are mentioned here already indicates that this statement is quite general, and I think a similar statement could have been made if the burst of TE activity fell anywhere between 10 and about ma 40 ago. The question thus is how meaningful this coincidence in timing really is, especially considering that, presumably, the likely statistical inference of such TE bursts may restricted to a certain time window (?).<br><br>We changed this section to address the reviewers comments.

- "It is thus likely that the diversification of birds-of-paradise was influenced by lineage specificity of their TE repertoires through retroviral germline invasions and smaller activity bursts". An in my view completely unwarranted statement, mainly due to the qualifier "likely". Instead, I would suggest a qualifier along the lines of "An intriguing hypothesis that warrants further investigation".

Changed.

* In the discussion of genes and GO categories, please state more clearly in which GO test the genes in question were flagged: positive selection or gene family size evolution.

We removed the GO enrichment for the positive selection analysis.

* p. 6, l. 34-36: "Dale and colleagues recently showed that sexual selection on male ornamentation in birds has antagonistic effects, where male coloration is increasing, while females show a strong reduction in coloration [37]. This is very apparent in polygynous core birds-of-paradise, where females between species and sometimes even between genera look highly similar." Increasing and decreasing "coloration"? "Ornamentation" would be a better choice of word. Also, a reduction in ornamentation/coloration is not the same thing as a reduced tempo of evolution, which is clearly referred to in the second sentence. Thus, expand/clarify.

We changed “coloration” to “ornamentation”. We only discuss phenotypes, not the tempo of their evolution in the second sentence.

* Methods, Gene Annotation section: "We did not train the de novo gene predictor Augustus [109] because no training data set for birds was available." Please expand - none of the gene sets from previously published bird genomes can be used?

We added a sentence, why we did not carry out the training at the time, which was decided after a discussion with Mario Stanke, the author of Augustus.

* Methods, Phylogenetic Analysis section: "We used the individual exon phylip files for gene tree reconstruction" - Why were only single exons used for each gene? I worry whether these were sufficiently informative, especially considering the high discordance among gene trees.

We clarified this. All exons for a gene were concatenated.

Minor comments:
* Italicize genus names throughout the paper.

We changed this.

*p.2, l. 19: "Ernst Mayer" - Remove the "e" in Mayer.

Changed.

* p. 3, l. 4-6: Change discussion of Irestedt et al. 2009 findings to past tense.

Changed.

* p. 3, l. 14: "specialized on" - reformulate.

Changed.

* p. 3, l. 34: "Only recently, have these" - Remove comma.

Changed.

* p. 3, l. 59-60: "All assemblies showed a genome size around 1 Gb": Assembly size, not genome size. Also, give the range of assembly lengths, and refer to Table S1.

	Changed.<br><br>* p. 5, l. 8: "taxa" - change to "taxon".<br><br>Changed.<br><br>* p. 8, l. 43-46: Sentence starting with "Congruently" - rephrase. In the next sentence, delete "the".<br><br>Changed.<br><br>* p. 10, l. 57: "On the contrary to other vertebrates" - Change to "Contrary to ...".<br><br>Changed.<br><br>* p. 11, l. 41: "straight forward" - Change to "straightforward".<br><br>Changed.<br><br>* Methods, Ortholog Gene Calling section: "For a gene to be included in our analyses, it had to be present in at least 75% of all species (7 out of 8 species)" - 75% would be 6 (not 7) out of 8 species…<br><br>Changed.<br><br>* Methods, Intron Calling section: "To do so we used the extract_intron_gff3_from_gff3.py script (https://github.com/irusri/Extract-intron-from-gff3) to include intron coordinates into the gff file. We then parsed out all intron coordinates and extracted the intron sequences from the genomes using the exttract_seq_from_gff3.pl script (https://github.com/irusri/Extract-intron-from-gff3)." - The same script is referenced twice in the actual link, but with a different name. Furthermore, I assume "exttract_seq_from_gff3.pl" has a typo ("exttract").<br><br>Both scripts can be found using the same link. Furthermore, the "exttract" is a typo by the author of the script, so we did not correct it as this is the actual name of the script.<br><br>*Methods, Inference of Positive Selection section: "due to the fact that branch-site tests result in an access of non-significant p-values" - Change "access" to "excess".<br><br>Changed.
**Additional Information:**	
**Question**	**Response**
Are you submitting this manuscript to a special series or article collection?	No
**Experimental design and statistics**<br><br>Full details of the experimental design and statistical methods used should be given in the Methods section, as detailed in our Minimum Standards Reporting Checklist. Information essential to interpreting the data presented should be made available in the figure legends.<br><br>Have you included all the information requested in your manuscript?	Yes

Powered by Editorial Manager® and ProduXion Manager® from Aries Systems Corporation

**Resources**<br><br>A description of all resources used, including antibodies, cell lines, animals and software tools, with enough information to allow them to be uniquely identified, should be included in the Methods section. Authors are strongly encouraged to cite Research Resource Identifiers (RRIDs) for antibodies, model organisms and tools, where possible.<br><br>Have you included the information requested as detailed in our Minimum Standards Reporting Checklist?	Yes
**Availability of data and materials**<br><br>All datasets and code on which the conclusions of the paper rely must be either included in your submission or deposited in publicly available repositories (where available and ethically appropriate), referencing such data using a unique identifier in the references and in the "Availability of Data and Materials" section of your manuscript.<br><br>Have you have met the above requirement as detailed in our Minimum Standards Reporting Checklist?	No
If not, please give reasons for any omissions below.<br><br>as follow-up to "**Availability of data and materials**<br><br>All datasets and code on which the conclusions of the paper rely must be either included in your submission or deposited in publicly available repositories (where available and ethically appropriate), referencing such data using	We will upload the data to the GigaDB database when requested, and given a link to the ftp site. All the raw read data is currently getting uploaded to NCBI SRA.

a unique identifier in the references and in the “Availability of Data and Materials” section of your manuscript.<br><br>Have you have met the above requirement as detailed in our Minimum Standards Reporting Checklist?<br><br>"	

Manuscript
Click here to access/download;Manuscript;BOP_resubmission_changes_corre
Click here to view linked References

Comparative analyses identify genomic features potentially involved in the evolution of birds-of-paradise.

Stefan Prost[1,2,*], Ellie E. Armstrong[3], Johan Nylander[1], Gregg W.C. Thomas[4], Alexander Suh[5], Bent Petersen[6,7], Love Dalen[1], Brett W. Benz[8], Mozes P.K. Blom[1], Eleftheria Palkopoulou[1], Per G. P. Ericson[1], Martin Irestedt[1,*]

[1] Department of Biodiversity Informatics and Genetics, Swedish Museum of Natural History, SE-10405 Stockholm, Sweden
[2] Department of Integrative Biology, University of California, Berkeley, CA 94720-3140, USA
[3] Department of Biology, Stanford University, Stanford, CA 94305-5020, USA
[4] Department of Biology and School of Informatics, Computing, and Engineering, Indiana University, IN 47405, USA
[5] Department of Evolutionary Biology (EBC), Uppsala University, 75236 Uppsala, Sweden
[6] Natural History Museum of Denmark, University of Copenhagen, 1353 Copenhagen, Denmark
[7] Centre of Excellence for Omics-Driven Computational Biodiscovery, Faculty of Applied Sciences, Asian Institute of Medicine, Science and Technology, Kedah, Malaysia.
[8] Department of Ornithology, American Museum of Natural History, New York, NY 10024, USA

* Corresponding authors: Stefan Prost (stefanprost.research@protonmail.com), Martin Irestedt (martin.irestedt@nrm.se)

Abstract

The diverse array of phenotypes and courtship displays exhibited by birds-of-paradise have long fascinated scientists and laymen alike. Remarkably, almost nothing is known about the genomics of this iconic radiation. There are 41 species in 16 genera currently recognized within the birds-of-paradise family (Paradisaeidae), most of which are endemic to the island of New Guinea. In this study, we sequenced genomes of representatives from all five major clades within this family to characterize genomic changes that may have played a role in the evolution of the group's extensive phenotypic diversity. We found genes important for coloration, morphology, feather and eye development to be under positive selection. In birds-of-paradise with complex lekking systems and strong sexual dimorphism, the core birds-of-paradise, we found GO categories for 'startle response' and 'olfactory receptor activity' to be enriched among the gene families expanding significantly faster compared to the other birds in our study. Furthermore, we found novel families of retrovirus-like retrotransposons active in all three *de novo* genomes since the early diversification of the birds-of-paradise group, which might have played a role in the evolution of this fascinating group of birds.

Keywords: *birds-of-paradise, comparative genomics,* gene gain-loss, positive selection, genome structure

Background

'Every ornithologist and birdwatcher has his favourite group of birds. Frankly, my own are the birds of paradise and bowerbirds. If they do not rank as high in world-wide popularity as they deserve it is only because so little is known about them.'

Ernst Mayr (in Gilliard 1969 [1])

The spectacular morphological and behavioral diversity found in birds-of-paradise (Paradisaeidae) form one of the most remarkable examples in the animal kingdom of traits that are thought to have evolved via forces of sexual selection and female choice. The family is comprised of 41 recognized species divided into 16 genera [2], all of which are confined to the Australo-Papuan realm and the Moluccas islands (Indonesia). The birds-of-paradise have adapted to a wide variety of habitats ranging from tropical lowlands to high-altitude mountain forests [3] and in the process acquired a diverse set of morphological traits. Some species are sexually monomorphic and crow-like in appearance with simple mating systems, whereas others have complex courtship behaviors and display strong sexual dimorphism with males exhibiting elaborate feather ornaments that serve as secondary sexual traits [3]. As such, strong sexual and natural selection have likely acted in concert to produce the exquisite phenotypic diversity among members the Paradisaeidae.

While having attracted substantial attention from systematists for centuries, the evolutionary processes and genomic mechanisms that have shaped these phenotypes remain largely unknown. In the past, the evolutionary history of birds-of-paradise has been studied with morphological data [1], molecular distances [4, 5], and a single mitochondrial gene [6], but the conclusions have been largely incongruent. The most comprehensive phylogenetic study at present includes all 41 species and is based on DNA-sequence data from both mitochondrial (cytochrome B) and nuclear genes (ornithine decarboxylase introns ODC6 and ODC7)[7]. This study suggested that the birds-of-paradise started to diverge during late Oligocene or early Miocene and could be divided into five main clades. The sexually monomorphic genera *Manucodia*, *Phonygammus*, and *Lycocorax* form a monophyletic clade (Clade A; Fig. 1 in Irestedt et al. [7]), which was suggested to be sister to the other four clades that include species, many of which show strong sexual dimorphism (here referred to as "core birds-of-paradise"). Among the latter four clades, the genera *Pteridophora* and *Parotia* were suggested to form the earliest diverging clade (Clade B; Fig. 1 in Irestedt et al. [7]), followed by a clade consisting of the genera *Seleucidis*, *Drepanornis*, *Semioptera*, *Ptiloris*, and *Lophorina* (Clade C; Fig. 1 in Irestedt et al. [7]). The last two sister clades are comprised of *Epimachus*, *Paradigalla*, and *Astrapia* (Clade D; Fig.

1 in Irestedt et al. [7]), and *Diphyllodes*, *Cicinnurus*, and *Paradisaea* (Clade E; Fig. 1 in Irestedt et al. [7]), respectively. Overall, the phylogenetic hypothesis presented in Irestedt et al. [7] receives strong branch support (posterior probabilities), but several nodes are still weakly supported and there is incongruence among gene trees. Recently, Irestedt and colleagues [8] and Scholes and Laman [9] argued for the Superb birds-of-paradise to be split into several species, based on genetics, morphology and courtship behavior. Thus, while preliminary genetic analyses have outlined the major phylogenetic divisions, the interspecific relationships of birds-of-paradise remain largely unresolved.

Birds-of-paradise are most widely known for their extravagant feather types, coloration and mating behaviors [3]. In addition, they also exhibit an array of bill shapes (often specialized to their respective foraging behavior), body morphologies, and sizes [3]. Ornament feather types include 'wire-type' tail feathers (e.g. twelve-wired bird-of-paradise (*Seleucidis melanoleuca*)), erectile head plumes (e.g. king of Saxony bird-of-paradise (*Pteridophora alberti*)), significantly elongated tail feathers (e.g. ribbon-tailed Astrapia (*Astrapia mayeri*)) or finely-filamental flank plumes (e.g. lesser bird-of-paradise (*Paradisaea minor*); see Frith and Beehler [3]). Feathers and coloration are crucial components of their mating displays. Polygynous birds-of-paradise show aggregated leks high in tree tops, less aggregated leks on lower levels or the forest floor (often exploded leks), and even solitary mating displays [3].

Coloration in birds-of-paradise involve both pigment-based and structural mechanisms. Coloration via pigmentation is achieved by pigment absorption of diffusely scattered light in a specific wavelength range. Pigments such as carotenoids are frequently associated with red and yellow hues in birds, whereas light absorption by various classes of melanin pigments give rise to black plumage features common in many birds-of-paradise species [10]. On the other hand, structural coloration is caused constructive interference and light reflection from quasiordered spongy structures of the feather barbs and melanosomes in feather barbules [11, 12]. The plumages of male birds-of-paradise feature both coloration types to various degrees, and some species such as the Lawe's parotia (*Parotia lawesii*) use angular-dependent spectral color shifts of their structural feathers in their elaborate display rituals to attract females [13, 14] Dale and colleagues recently showed that sexual selection on male ornamentation in birds has antagonistic effects, where male ornamentation is increasing, while females show a strong reduction in ornamentation [15]. This is very apparent in polygynous core birds-of-paradise, where females between species and sometimes even between genera look highly similar.

The array of extravagant phenotypes found in birds-of-paradise makes them an interesting model to study evolution. However, fresh tissue samples from birds-of-paradise are extremely limited and currently only about 50% of all species are represented in biobanks. Fortunately, the current revolution in sequencing technologies and laboratory methods does not only enable us to sequence whole-genome data from non-model organisms, but it also allows us to harvest genome information from specimens in museum collections (by using sequencing library preparation methods specifically designed for degraded DNA in combination with high-throughput sequencing) [16]. Only recently have these technological advances enabled researchers to

investigate genome-wide signals of evolution using comparative and population genomic approaches in birds [17-20].

In the current study, we made use of these technological advances to generate *de novo* genomes for three birds-of-paradise species from fresh samples and re-sequenced the genomes of two other species from museum samples. Using these genomes, we were able to contrast the trajectory of genome evolution across passerines and simultaneously evaluate which genomic features have evolved during the radiation of birds-of-paradise. We identified a set of candidate genes and genomic features that might have contributed to the extraordinary diversity in phenotypic traits found in birds-of-paradise.

Results

Assembly and gene annotation

We *de novo* assembled the genomes of *Lycocorax pyrrhopterus*, *Ptiloris paradiseus* and *Astrapia rothschildi* using paired-end and mate pair Illumina sequence data, and performed reference-based mapping for *Pteridophora alberti* and *Paradisaea rubra*. Scaffold N50 ranged from 4.2 Mb (L. *pyrrhopterus*) to 7.7 Mb (*A. rothschildi*), and the number of scaffolds from 2,062 (*P. paradiseus*) to 3,216 (*L. pyrrhopterus*; Table 1). All assemblies showed a genome assembly size around 1 Gb (1.03 to 1.07 Gb, see Table 1). BUSCO2 [21] scores for complete genes (using the aves_odb9 database) found in the respective assemblies ranged from 93.8% to 95.1%, indicating a high completeness (Supplementary Table S1). Next, we annotated the genomes using homology to proteins of closely related species as well as *de novo* gene prediction. Gene numbers ranged from 16,260 (*A. rothschildi*) to 17,269 (*P. paradiseus;* see Supplementary Table S2).

Repeat evolution in birds-of-paradise

Our *de novo* repeat annotation analyses (Supplementary Table S3) suggest that the genomes of birds-of-paradise contain repeat densities (~7%) and compositions (mostly chicken repeat 1 (CR1) long interspersed nuclear elements (LINEs), followed by retroviral long terminal repeats (LTRs)) well within the usual range of avian genomes [22]. However, we identified 16 novel LTR families (solo LTRs; Supplementary Table S4) with no sequence similarity to each other or to LTR families known from in-depth annotations of chicken (*Gallus gallus*), zebra finch (*Taeniopygia guttata*), and collared flycatcher (*Ficedula albicollis*) [23, 24]. Interestingly, we find that activity of CR1 LINEs ceased recently in the three birds-of-paradise and was replaced by activity of retroviral LTRs (Fig. 1). The inferred timing of the TE (transposable element) activity or accumulation peak (Fig. 1) roughly corresponds to or slightly predates the radiation of birds-of-paradise (inferred in Irestedt et al. 2009 [7]). We also found that the genome assembly of *Lycocorax pyrrhopterus* exhibits slightly higher repeat densities than those of the two other birds-of-paradise (Supplementary Table S3) and slightly more recent TE activity (Fig. 1). A possible explanation for this is that this is the only female bird-of-paradise assembly, thus containing the female-specific W chromosome which is highly repetitive [22].

Genome synteny to the collared flycatcher

We found strong synteny of the three *de novo* assembled birds-of-paradise genomes (*Lycocorax pyrrhopterus*, *Ptiloris paradiseus, Astrapia rothschildi*) to that of the collared flycatcher (Fig. 2 and Supplementary Figures S1-3). Only few cases were found where individual scaffolds of the birds-of-paradise genomes mapped to multiple chromosomes in the collared flycatcher genome.

Phylogeny

We found 4,656 one-to-one orthologous genes to be present in all eight sampled bird genomes (5 birds-of-paradise and 3 outgroup songbirds). A phylogeny inferred using these orthologs shows a topology with high bootstrap scores (Fig. 3 and Supplementary Figure S4). However, the sole use of bootstrapping or Bayesian posterior probabilities in analyses of large scale data sets has come into question in recent years [25]. Studies based on genome-wide data have shown that phylogenetic trees with full bootstrap or Bayesian posterior probability support can exhibit different topologies (e.g. Jarvis et al. [26] and Prum et al. [27]; discussed in Suh [25]). Thus, next we performed a concordance analysis by comparing gene trees for the 4,656 single-copy orthologs to the inferred species topology. We find strong concordance for the older splits in our phylogeny (see Supplementary Figure S4). However, the splits between *Ptiloris* and its sister clade, which contains *Astrapia* and *Paradisaea*, and the split between *Astrapia* and *Paradisaea* itself showed much lower concordance values, 0.31 and 0.26, respectively. Only ~10% of the gene trees exactly matched the topology of the inferred species tree and we find an average Robinson-Foulds distance of 3.92 for all gene trees compared to the species tree (Supplementary Table S5). A Robinson-Foulds distance of 0 would indicate that the two tree topologies (species to gene tree) are identical. The highest supported species tree topology (Fig. 3 and Supplementary Figure S4) is concordant to the birds-of-paradise species tree constructed in Irestedt et al. 2009 [7]. Overall, we find that the birds-of-paradise form a monophyletic clade, with the crow (*Corvus cornix*) being the most closely related sister taxon, in most gene trees (74%). Within the birds-of-paradise clade, we further distinguish a core birds-of-paradise clade, which consist of 4 of the 5 species in our sample (excluding only the paradise crow, *Lycocorax pyrrhopterus*).

Positive selection in the birds-of-paradise

We carried out positive selection analyses using all previously ascertained orthologous genes (8,134 genes present in at least seven out of the eight species) on the branch leading to the birds-of-paradise. First, we investigated saturation by calculating pairwise dN/dS ratios. The inferred values did not show any signs of saturation (Supplementary Table S6). To infer positive selection on the branch of the birds-of-paradise, we used the BUSTED model in HyPhy (similar to branch-sites model; [28]). We found 213 genes to be under selection (p value < 0.05; gene symbol annotation for 212 of the 213 genes can be found in Supplementary Table S7). Multiple-testing correction was carried out using FDR in R (function *p.adjust()* from the stats package).

Gene gain and loss

We identified 9,012 gene families across all 8 species. Using CAFE [29] we inferred 98 rapidly evolving families within the birds-of-paradise clade. Supplementary Table S8 summarizes the

gene family changes for all 8 species (also see Fig. 3). Zebra finch had the highest average expansion rate across all families at 0.0916, while the hooded crow had the lowest average expansion rate at -0.1724, meaning that they have the most gene family contractions. Gene gain/loss rates can be found in Supplementary Table S9. Next, we tested for enrichment of GO terms in the set of families rapidly evolving in the birds-of-paradise clade. Gene families were assigned GO terms based off the Ensembl GO predictions for flycatcher and zebra finch. In all, we were able to annotate 6,350 gene families with at least one GO term. Using a Fisher's exact test on the set of 98 rapidly evolving families in the birds-of-paradise, we find 25 enriched GO terms in 20 families (FDR 0.05; Supplementary Table S10). All the gene gain and loss results can be found online (https://cafe-portal.gitlab.io/bop/).

Discussion

Renowned for their extravagant plumage and elaborate courtship displays, the birds-of-paradise are among the most prominent examples of how sexual selection can give rise to extreme phenotypic diversity. Despite extensive work on systematics and a long-standing interest in the evolution of their different mating behaviors, the genomic changes that underlie this phenotypic radiation have received little attention. Here, we have assembled representative genomes for the five main birds-of-paradise clades and characterized differences in genome evolution within the family and relative to other avian groups. We reconstructed the main structure of the family phylogeny, inferred the TE (transposable element) landscape and identified a list of genes under selection and gene families significantly expanded or contracted potentially involved in many phenotypic traits for which birds-of-paradise are renowned. Below, we discuss these different genomic features and how they might have contributed to the evolution of birds-of-paradise.

Genome synteny and phylogeny

We found genome synteny (here in comparison to the collared flycatcher [30]) to be highly conserved for all three *de novo* assembled genomes (Fig. 2 and Supplementary Figures S1-3). Only few cases were recorded where regions of scaffolds of the birds-of-paradise genomes aligned to different chromosomes of the collared flycatcher. These could be artifacts of the genome assembly process or be caused by translocations. Passerine birds show variable numbers of chromosomes (72-84 [22]). However, they do not vary as much as other groups', such as Charadriiformes (shorebirds, 40-100 [22]). In general, studies have shown a high degree of genome synteny even between Galloanseres (galliform and anseriform birds) and Neoaves (most other birds including passerines; approx. 80-90 mya divergence; reviewed in Ellegren [31]; Poelstra et al. [17]). However, genomes with higher continuity, generated with long-read technologies or using long-range scaffolding methods (such as Hi-C [32]), or a combination thereof, will be needed to get a more detailed view of rearrangements in birds-of-paradise genomes (see Peona et a. [33]).

Our analyses reconstructed a phylogenetic tree topology congruent with the one presented in Irestedt et al. [7] (Fig. 3 and Supplementary Figure 4). However, while bootstrapping found strong support for the topology of the species tree, congruence analysis found high discordance for the two most recent branches (*Ptiloris* and its sister clade (*Astrapia* and *Paradisaea*) and the split between *Astrapia* and *Paradisaea*). Furthermore, we found the highest supported tree topology to be based on only 10% of all gene trees (Supplementary Table S5). This could be caused by incomplete lineage sorting (ILS), which refers to the persistence of ancestral polymorphisms across multiple speciation events [34]. Jarvis et al. [26] and Suh et al. [35] showed that ILS is a common phenomenon on short branches in the avian Tree of Life. Another possibility could be hybridization, a phenomenon frequently recorded in birds-of-paradise [3]. Overall, most gene tree topologies (74%) support the monophyly for the birds-of-paradise and the core birds-of-paradise clades.

Repeats and their possible role in the evolution of birds-of-paradise

Bursts of TE activity are often lineage- or species-specific, which highlights their potential to affect speciation [30]. This is further supported by the fact that TE activity bursts often correlate with the speciation timing of the respective species or species group [30]. Similarly, we find a burst of LINE activity within all three *de novo* assembled genomes (*Lycocorax pyrrhopterus*, *Ptiloris paradiseus*, *Astrapia rothschildi*) dating back to about 24 mya (Fig. 1). The timing roughly corresponds to or slightly predates the emergence and radiation of birds-of-paradise (see Fig 3). The notion that we found 16 novel families of retroviral LTRs suggests multiple recent germline invasions of the birds-of-paradise lineage by retroviruses. The recent cessation of activity of CR1 LINEs and instead recent activity of retroviral LTRs (Fig. 1) is in line with similar trends in collared flycatcher and hooded crow [22, 24]. This suggests that recent activity of retroviral LTRs might be a general genomic feature of songbirds, however, with different families of retroviruses being present and active in each songbird lineage. Thus, an intriguing hypothesis that warrants further investigation is that the diversification of birds-of-paradise (and probably songbirds in general) was influenced by lineage specificity of their TE repertoires through recurrent retroviral germline invasions and smaller activity bursts.

Coloration, feather and skeletal development in birds-of-paradise

Given the strong sexual dimorphism and array of morphological phenotypes found in birds-of-paradise and their important role in mating success, we would expect genes important for coloration, morphology and feather structure to be under selection in the birds-of-paradise. Indeed, we found several genes potentially involved in these phenotypic traits to show signatures of positive selection. However, most of them were non-significant after the multiple-testing correction (see Supplementary Table S7). Given that these corrections are often very conservative, these genes might still warrant more detailed follow-up investigations. One such gene is ADAMTS20, which is crucial for melanocyte development. ADAMTS20 has been shown to

cause white belt formation in the lumbar region of mice [36]. Nonsense or missense mutations in this gene disrupt the function of KIT, a protein that regulates pigment cell development [36]. In mammals and birds, pigment patterns are largely influenced by melanocytes. Thus, this gene could be a strong candidate for differential coloration in the birds-of-paradise. Another gene under positive selection with a potential role in coloration is ATP7B. It is a copper-transporting P-type ATPase and thought to translate into a melanosomal protein (see Bennett and Lamoreux [37] for a review). Copper is crucial for melanin synthesis because tyrosinase contains copper and thus ATP7B might play a crucial role in pigment formation. Candidate genes for positive selection, which are known to have functions in feather development (in chicken) and morphology (in chicken and mammals) include FGR1, SPECC1L, BMPR1A, GAB2, PAPSS2, DCST2, ALDH3A2, MYF5 and APOBEC2. For example, FGFR1 (Fibroblast growth factor receptor 1) is implicated in feather development [38]. In humans it has further been shown to be involved in several diseases associated with craniofacial defects (OMIM; http://www.ncbi.nlm.nih.gov/omim/). ALDH3A2 (aldehyde dehydrogenase 3 family member A2), a membrane-associated protein and SPECC1L are implicated in craniofacial disorders (e.g. Van Der Woude Syndrome) in humans [39]. APOBEC2 seems to play a role in muscle development (skeletal and heart muscle) in chickens [40].

APOBECs and their potential role in the immune system

Intriguingly, APOBECs have also been shown to have important functions in the immune systems of vertebrates, where they act as restriction factors in the defense against a range of retroviruses and retrotransposons [41, 42]. Functioning as cytosine deaminases they act against endogenous retroviruses (ERVs), especially Long terminal repeat retrotransposons (LTR) by interfering with the reverse transcription and by hypermutating retrotransposon DNA. A recent study on 123 vertebrates showed that birds have the strongest hypermutation signals, especially oscine passerines [43] (such as zebra finch and medium ground finch). This study also demonstrated that edited retrotransposons may preferentially be retained in active regions of the genome, such as exons and promoters because hypermutation decreases their potential for mobility. Thus, it seems very likely that retrotransposon editing via APOBECs has an important role in the innate immunity of vertebrates as well as in genome evolution. In line with this hypothesis, we found a burst in recent activity of retroviral LTRs in the genomes of birds-of-paradise, a signal also found in other passerines [22, 24]. This could also explain why we found APOBEC2 to be under positive selection.

Sensory system in the birds-of-paradise

Visual system

Genes that showed positive selection signals and are known to have known roles in eye function and development include CABP4, NR2E3, IMPG1, AKAP13, MGARP, GNB1, ATP6AP2 and MYOC, of which the latter three remained significant after multiple-testing correction. Interestingly, there are no obvious explanations for selection on vision in birds-of-paradise. Evidence

for co-evolution between coloration and vision in birds is weak (see e.g. Lind et al. [44], Price [45], but see Mundy et al. [46] and Bloch [47]). A phenotype that could be associated with selection on vision is the diverse array of mating displays in some core birds-of-paradise. Many species, such as Lawes's parotia (*Parotia lawesii*) modify color by changing the angle of the light reflection [13, 14], which requires the visual system to be able to process the fine nuances of these color changes. However, the fact that (color) vision serves many purposes (including e.g. foraging, etc.) makes it very difficult to establish co-evolution between coloration and color vision [44]. We can thus only speculate at this point about the potential role of coloration or mating displays in the selection of vision genes found in birds-of-paradise.

Olfactory system

Another often overlooked sensory system in birds is odor perception. Olfactory receptors (ORs) are important in odor perception and detection of chemical cues. In many animal taxa, including birds, it has been shown that olfaction is crucial to identify species [48], relatedness [49], individuals [50], as well as for mate choice [51] and in foraging [52]. In concordance with previous studies, we found this gene family to expand rapidly in the zebra finch [53]. Even more so, the zebra finch showed the strongest expansion (+17 genes) in our data set. Furthermore, we find a rapid expansion on the branch leading to the core birds-of-paradise (+5 genes) and further in *Astrapia* (+6 genes). Interestingly, olfactory receptor genes show rapid contractions in the paradise crow (-6 genes), the hooded crow (-9 genes) and the collared flycatcher (-5 genes). This is in line with a study that suggested poor olfactory development in a different corvid species, the Japanese jungle crow (*Corvus macrorhynchos*) [54]. Olfactory could serve many functions in birds-of-paradise such as species recognition (to avoid extensive hybridization), individual recognition, mating or foraging (given their extensive diet breadth).

Startle response and adult locomotory behavior

Startle response is an important behavioral trait. It is the ability to react quickly to the presence of a stimulus. We find a gene family associated with startle response and adult locomotory behavior to be evolving significantly faster than under a neutral model on the branch leading the core birds-of-paradise (+5 genes). It is even further expanded in *Paradisaea* (+3 genes). This gene family is contracted in the two outgroups, the zebra finch (-4 genes) and the collared flycatcher (-2 genes), as well as the monochromatic, non-lekking paradise crow (-1 genes). We found no expansion or contraction in the hooded crow genome. For core birds-of-paradise that show extravagant lekking behavior, fast response to stimuli or increased locomotory behavior could have several advantages. For example, being highly visible during leks means that lekking birds need to be able to look out for predators and react to them quickly, and indeed Frith & Beehler [3] mention that lekking birds-of-paradise appear to be constantly on the lookout for predators. Interestingly, species of the genus *Paradisaea* have aggregated leks high in emergent trees and thus may be more visible to the numerous birds of prey that inhabit the region [3]. It could further be important for interaction between males or between the sexes during leks.

Conclusions

We found indications of a conserved synteny between birds-of-paradise and other passerine birds, such as the collared flycatcher. Similar to other passerine genomes, we also found signatures of recent activity of novel retroviral LTRs in the genomes. Furthermore, several genes with known function in coloration, feather, and skeletal development showed signatures of positive selection in birds-of-paradise. This is in accordance with our prediction that phenotypic evolution in birds-of-paradise should have left genomic signatures. While these genes all are obvious candidates for evolution of birds-of-paradise's phenotypic and behavioral diversity, we also found positively selected genes that are not as straightforward to explain. These include genes involved in development and function of the visual system. Gene gain/loss analyses further revealed significant expansions in gene families associated with 'startle response/locomotory behavior' and olfactory function.

Although the efforts to document the phenotypic and behavioral diversity in this model system of sexual selection continues to generate intense interest in birds-of-paradise, we still have limited understanding of the processes that have shaped their evolution. Here, we provide a first glimpse into genomic features underlying the diverse array of species found in birds-of-paradise. However, our analyses concentrate on comparative genomics, and we acknowledge that analyses based on multiple individuals per species will likely result in more reliable inferences. Furthermore, some of the most interesting traits in birds-of-paradise, such as lekking behavior, are likely complex traits and will require analyses based on many individuals (such as QTL mapping). In general, more in-depth analyses will be needed to establish a causal relationship between signatures of selection in the birds-of-paradise genome and the unique diversity of phenotypic traits, or to investigate changes in genome structure with higher resolution. Fortunately, technologies keep advancing, and along with decreasing costs for sequencing, we will soon be able to gain more information about this fascinating, but genetically understudied family of birds.

Data description and Analyses

Sampling and DNA extraction

For the three *de novo* genome assemblies, *Lycocorax pyrrhopterus* (ZMUC149607; collected 2013, Obi Island, Indonesia), *Ptiloris paradiseus* (ANWC43271; collected 1990, New South Wales, Australia), and *Astrapia rothschildi* (KU Birds 93602; collected 2001, Morobe Province, Papua New Guinea) DNA was extracted from fresh tissue samples using the Qiagen QIAamp DNA Mini Kit according to the manufacturer's instructions. The *de novo* libraries with different insert sizes (see below) were prepared by Science for Life Laboratory, Stockholm. For the two re-sequenced genomes, *Pteridophora alberti* (NRM571458; collected 1951, Eastern Range, New Guinea) and *Paradisaea rubra* (NRM700233; collected 1949, Batanta Island, New Guinea), we sampled footpads and extracted DNA using the Qiagen QIAamp DNA Micro Kit according to the manufacturer's instructions. We applied precautions for working with museum samples described in Irestedt et al. [55]. Sequencing libraries for these two samples were prepared using the protocol published by Meyer and Kircher [56]. This method was specifically developed to

generate sequencing libraries for low input DNA, showing DNA damage typical for museum or ancient samples.

Genome Sequencing, Assembly, and Quality Assessment

We prepared two paired-end (overlapping and 450 bp average insert size) and two mate pair libraries (3 kb and 8 kb average insert size) for each of the three *de novo* assemblies (*Ptiloris paradiseus, Astrapia rothschildi*, and *Lycocorax pyrrhopterus)*. All libraries, for the *de novo* and the reference-based mapping approaches were sequenced on a HiSeq2500 v4 at SciLifeLab Stockholm, Sweden. We generated two lanes of sequencing for each *de novo* assembly and pooled the two reference-based samples on one lane. We first assessed the read qualities for all species using the program FastQC (FastQC, RRID:SCR_014583) [57]. For the three species, *Ptiloris paradiseus, Astrapia rothschildi*, and *Lycocorax pyrrhopterus* we then used the *preqc* [58] function of the SGA [59] assembler to (1) estimate the predicted genome size, (2) find the predicted correlation between k-mer sizes and N50 contig length and (3) assess different error statistics implemented in *preqc*. For *Ptiloris paradiseus, Astrapia rothschildi*, and *Lycocorax pyrrhopterus* reads were assembled using Allpaths-LG [60]. To improve the assemblies, especially in repeat regions, GapCloser (GapCloser, RRID:SCR_015026; part of the SOAPdenovo package [61]) was used to fill in gaps in the assembly. Assemblies were then compared using CEGMA (CEGMA, RRID:SCR_015055) [62] and BUSCO2 (BUSCO (RRID:SCR_015008)) [21]. We added BUSCO2 scores for better comparisons at a later stage of the project. For the reference-based mapping, we mapped all reads back to the *Ptiloris paradiseus* assembly using BWA (BWA, RRID:SCR_010910) [63] (mem option), the resulting sam file was then processed using samtools [64]. To do so, we first converted the sam file generated by BWA to the bam format, then sorted and indexed the file. Next we removed duplicates using Picard (Picard, RRID:SCR_006525) (http://broadinstitute.github.io/picard/) and realigned reads around indels using GATK (GATK , RRID:SCR_001876) [65]. The consensus sequence for each of the two genomes was then called using ANGSD [66] (using the option -doFasta 3). [Genomes will be deposited in GenBank]

Repeat Annotation

We predicted lineage-specific repetitive elements *de novo* in each of the three birds-of-paradise genome assemblies using RepeatModeler (RepeatModeler, RRID:SCR_015027) v. 1.0.8 [67]. RepeatModeler constructs consensus sequences of repeats via the three complementary programs RECON [68], RepeatScout (RepeatScout, RRID:SCR_014653) [69], and Tandem Repeats Finder [70]. Next, we merged the resultant libraries with existing avian repeat consensus sequences from Repbase [71] (mostly from chicken and zebra finch), and recent in-depth repeat annotations of collared flycatcher [24, 72] and hooded crow [73]. Redundancies among the three birds-of-paradise libraries, and between these and existing avian repeats were removed using the ReannTE_mergeFasta.pl script (https://github.com/4ureliek/ReannTE/). For *Lycocorax pyrrhopterus* repeats, we manually inspected the RepeatModeler library of consensus sequences for reasons reviewed in Platt et al. [74] and because *Lycocorax pyrrhopterus* was the most repeat-rich genome among the three birds-of-paradise. Manual curation was performed using standard procedures [35, 75], namely screening of each repeat candidate against the *Lycocorax*

pyrrhopterus assembly using blastn [76], extracting the 20 best hits including 2-kb flanks, and alignment of these per-candidate BLAST hits to the respective consensus sequence using MAFFT (MAFFT, RRID:SCR_011811) v. 6 [77]. Each alignment was inspected by eye and curated majority-rule consensus sequences were generated manually considering repeat boundaries and target site duplication motifs. This led to the identification of 33 long terminal repeat (LTR) retrotransposon consensus sequences (including 16 novel LTR families named as 'lycPyrLTR*') and three unclassified repeat consensus sequences (Supplementary Table S4). We then used this manually curated repeat library of *Lycocorax pyrrhopterus* to update the aforementioned merged library of avian and birds-of-paradise repeat consensus sequences. Subsequently, all three birds-of-paradise genome assemblies were annotated via RepeatMasker (RepeatMasker, RRID:SCR_012954) v. 4.0.6 and '-e ncbi' [78] using this specific library (Supplementary Table S3). Landscapes of relative TE activity (i.e., the amount of TE-derived bp plotted against Kimura 2-parameter distance to respective TE consensus) were generated using the calcDivergenceFromAlign.pl and createRepeatLandscape.pl scripts of the RepeatMasker package. To enhance plot readability, TE families were grouped into the subclasses "DNA transposon", "SINE", "LINE", "LTR", and "Unknown" (Fig. 1). We scaled the Kimura substitution level with the four-fold degenerate mutation rate for Passeriformes (mean of 3.175 substitutions/site/million years for passerines sampled in Zhang et al. [19]) to obtain an estimate of the timing of the inferred repeat activity in million years (mya).

Genome Synteny

We also inferred genome architecture changes (synteny) between our three *de novo* assembled genomes and the chromosome-level assembly of the collared flycatcher [30]. To do so, we first performed pairwise alignments using Satsuma [79] and then plotted the synteny using Circos plots [80]. More precisely, we first performed asynchronous 'battleship'-like local alignments using *SatsumaSynteny* to allow for time efficient pairwise alignments of the entire genomes. In order to avoid signals from repetitive elements we used masked assemblies for the alignments. Synteny between genomes was then plotted using Circos and in-house perl scripts.

Gene Annotation

We masked repeats (only transposable elements) in the genome prior to gene annotation. Contrary to the repeat annotation step, we did not mask simple repeats in this approach. Those were later soft-masked as part of the gene annotation pipeline Maker2 [81], to allow for more efficient mapping during gene annotation.

Gene annotation was performed using *ab-initio* gene prediction and homology-based gene annotation. To do so we used the genome annotation pipeline Maker2 [81], which is able to perform all the aforementioned genome annotation strategies. Previously published protein evidence (genome annotations) from Zhang et al. [19] were used for the homology-based gene prediction. To improve the genome annotation we used CEGMA to train the *ab-initio* gene predictor SNAP [82] before running Maker2 [81]. We did not train the *de novo* gene predictor Augustus [83] because no training data set for birds was available, and it was not recommended at the time to use potentially lower quality annotations for training of Augustus (M. Stanke, personal communication).

Ortholog Gene Calling

In the next step we inferred orthologous genes using PoFF [84]. We included all five birds-of-paradise, as well as the hooded crow (*Corvus cornix)* [17], the zebra finch (*Taeniopygia guttata*) [85] and the collared flycatcher (*Ficedula albicollis*) [30] as outgroups. We ran PoFF using both the transcript files (in fasta format) and the transcript coordinates file (in gff3 format). The gff files were used (flag –*synteny*) to calculate the distances between paralogous genes to accurately distinguish between orthologous and paralogous genes. We then extracted the sequences for all one-to-one orthologs using a custom python script.

Next, we determined the number of genes with missing data in order to maximize the number of genes included in the subsequent analyses. For a gene to be included in our analyses, it had to be present in at least 75% of all species (6 out of 8 species), which resulted in a set of 8,134 genes. In order to minimize false positives in the subsequent positive selection analysis caused by alignment errors, we used the codon-based alignment algorithm of Prank [86] and further masked sites with possible alignment issues using Aliscore [87]. Aliscore uses Monte Carlo resampling within sliding windows to identify low-quality alignments in amino acid alignments (converted by the program). The identified potential alignment issues were then removed from the nucleotide alignments using ALICUT [88].

Intron Calling

In addition to exons, we also extracted intron information for the birds-of-paradise genomes (see Supplementary Table S2). To do so we used the extract_intron_gff3_from_gff3.py script (https://github.com/irusri/Extract-intron-from-gff3) to include intron coordinates into the gff file. We then parsed out all intron coordinates and extracted the intron sequences from the genomes using the exttract_seq_from_gff3.pl script (https://github.com/irusri/Extract-intron-from-gff3). All introns for the same gene were then concatenated using a custom python script.

Phylogenetic analysis

The individual alignment files (we used exon sets without missing species, which resulted in 4,656 alignments) were then (1) converted to the phylip format individually, and (2) 200 randomly selected exon alignments were concatenated and then converted to phylip format using the catfasta2phyml.pl script (https://github.com/nylander/catfasta2phyml). We used the individual exon phylip files (all exons combined per gene) for gene tree reconstruction using RaxML [89] (using the GTR + G model). Subsequently, we combined the gene trees into a species tree using a multi-species coalescent model and carried out bootstrapping using Astral [90, 91]. Astral does not provide branch lengths needed for calibrating phylogenetic trees (used for the gene gain/loss analysis). So, we subsampled our data, and constructed a ML tree based on 200 randomly chosen and concatenated exons using ExaML [92]. We then calibrated the species tree using the obtained branch lengths along with calibration points obtained from timetree.org using r8s [93]. These calibration points are the estimated 44 mya divergence time between flycatcher and zebra finch and the 37 mya divergence time between crow and the birds-of-paradise.

Next, we performed a concordance analysis. First, we rooted the gene trees based on the outgroup (collared flycatcher, zebra finch). Then, for each node in the species tree we counted the number of gene trees that contained that node and divided that by the total number of gene trees. We next counted the number of gene trees that support a given topology (see Supple-

mentary Table S65) and further calculated the Robison-Foulds distance between gene trees using RaxML.

Inference of Positive selection

We inferred genes under positive selection using *dN/dS* ratios of 8,133 orthologs. First, we investigated saturation of synonymous sites in the phylogenetic sampling using pairwise comparisons in CodeML [94]. The pairwise runmode of CodeML estimates *dN* and *dS* ratios using a ML approach between each species pair (Supplementary Table S6). We then investigated positive selection on the branch to the birds-of-paradise using the BUSTED model [28] (branch-site model) implemented in HyPhy [95]. The branch-site test allows for inference of positive selection in specific branches (foreground branches) compared to the rest of the phylogeny (background branches). The significance of the model comparisons was determined using likelihood-ratio tests (LRT). We carried out multiple testing corrections using FDR [96] instead of Bonferroni correction, as the branch-site tests result in an excess of non-significant p-values, which violates the assumption of a uniform distribution in multiple testing correction methods such as the Bonferroni correction. The genes were then assigned gene symbols. To do so, we first extracted all the respective zebra finch or collared flycatcher GeneBank protein accessions. We then converted the accessions into gene symbols using the online conversion tool bioDBnet [97]. GO terms were obtained for the flycatcher assembly and assigned to orthologs that had a corresponding flycatcher transcript ID in Ensembl (7,305 genes out of 8,133). To determine enriched GO categories in positively selected genes, GO terms in genes inferred to have undergone positive selection were then compared to GO terms in all genes (with a GO term) using Fisher's exact test with a false discovery rate cut-off of 0.05. We found 262 GO terms enriched in positively selected genes before FDR correction and 47 enriched after.

Gene Gain-Loss

In order to identify rapidly evolving gene families in the birds-of-paradise we used the peptide annotations from all five birds-of-paradise species, along with the three outgroup species in our analysis: hooded crow*,* zebra finch and collared flycatcher. The crow genes were obtained from NCBI and the zebra finch and flycatcher genes were acquired from ENSEMBL 86 [98]. To ensure that each gene was counted only once, we used only the longest isoform of each protein in each species. We then performed an all-vs-all BLAST [76] search on these filtered sequences. The resulting e-values from the search were used as the main clustering criterion for the MCL program to group peptides into gene families [99]. This resulted in 13,289 clusters. We then removed all clusters only present in a single species, resulting in 9,012 gene families. Since CAFE requires an ultrametric time tree as input, we used r8s to smooth the phylogenetic tree with calibration points based on the divergence time of crow and the birds-of-paradise at 37 mya and of flycatcher and zebra finch at 44 mya [100].

With the gene family data and ultrametric phylogeny (Figure 3) as input, we estimated gene gain and loss rates (λ) with CAFE v3.0 [101]. This version of CAFE is able to estimate the amount of assembly and annotation error (ε) present in the input data using a distribution across the observed gene family counts and a pseudo-likelihood search. CAFE is then able to correct for this error and obtain a more accurate estimate of λ. We find an ε of about 0.01, which implies that 3% of gene families have observed counts that are not equal to their true counts. After correcting for

this error rate, we find $\lambda = 0.0021$. This value for λ is considerably higher than those reported for other distantly related groups (Supplementary Table S9). GO terms were assigned to genes within families based on flycatcher and zebra finch gene IDs from Ensembl. We used these GO assignments to determine molecular functions that may be enriched in gene families that are rapidly evolving along the ancestral BOP lineage (Node BOP11 in Figure S1). GO terms in genes in families that are rapidly evolving along the BOP lineage were compared to all other GO terms using a Fisher's exact test (FDR cut-off of 0.05). We found 36 genes in 26 families to have enriched GO terms before FDR correction and 25 genes in 20 families after.

DATA AVAILABILITY

All genomes and supporting data are available via the *GigaScience* repository, GigaDB [102], and raw read data via NCBI (SRA archive; BioProject: PRJNA506819, BioSample: SAMN10474331-SAMN10474335, SRA Study: SRP173170). All results of the gene gain-loss analyses can be found online [103].

Acknowledgement

We thank Australian National Wildlife Collection, CSIRO Sustainable Ecosystems (Leo Joseph) for tissue sample from *Ptiloris paradiseus*, Natural History Museum of Denmark, University of Copenhagen (Knud Jønsson and Jan Bolding) for tissue samples from *Lycocorax pyrrhopterus*, and Natural History Museum, and Biodiversity Institute, University of Kansas (Robert Moyle) for tissue samples of *Astrapia rothschildi.* We further thank Daniel Osorio for helpful discussion of coloration and color vision in birds, and Nagarjun Vijay for help with the circos plots. MI and PE were supported by the Swedish Research Council (grant number 621-2013-5161 to PE and grant number 621-2014-5113 to MI). We also acknowledge support from Science for Life Laboratory, the National Genomics Infrastructure (NGI), Uppmax and the EvoLab at the University of California Berkeley for providing resources for massive parallel sequencing and computational infrastructure. The funders had no role in study design, data collection and analysis, decision to publish, or preparation of the manuscript.

Figures

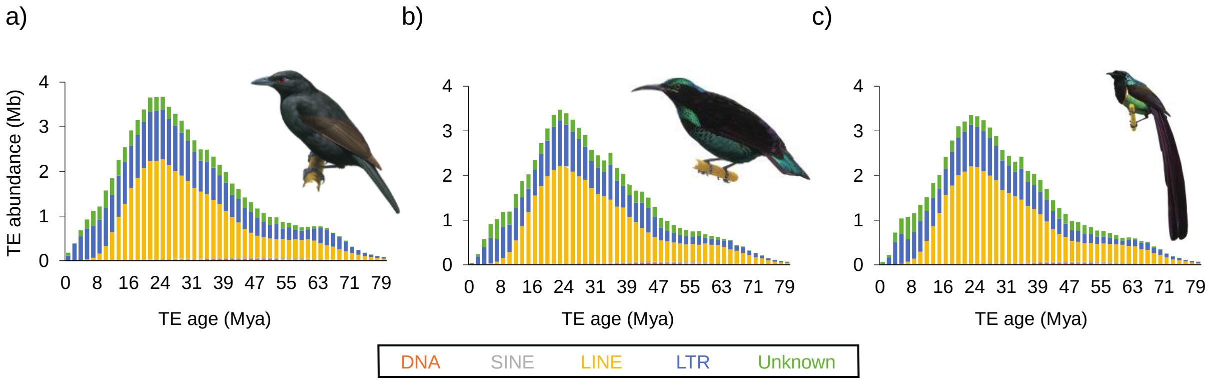

Figure 1: Repeat landscapes of *Lycocorax pyrrhopterus*, *Ptiloris paradisaeus*, and *Astrapia rothschildi*. Total amounts of TE-derived bp are plotted against relative age, approximated by per-copy Kimura 2-parameter distance to the TE consensus sequence and scaled using a four-fold degenerate mutation rate of Passeriformes of 3.175 substitutions/site/million years. TE families were grouped as "DNA transposons" (red), "SINEs" (grey), "LINEs" (yellow), "LTRs" (blue), and "Unknown" (green).

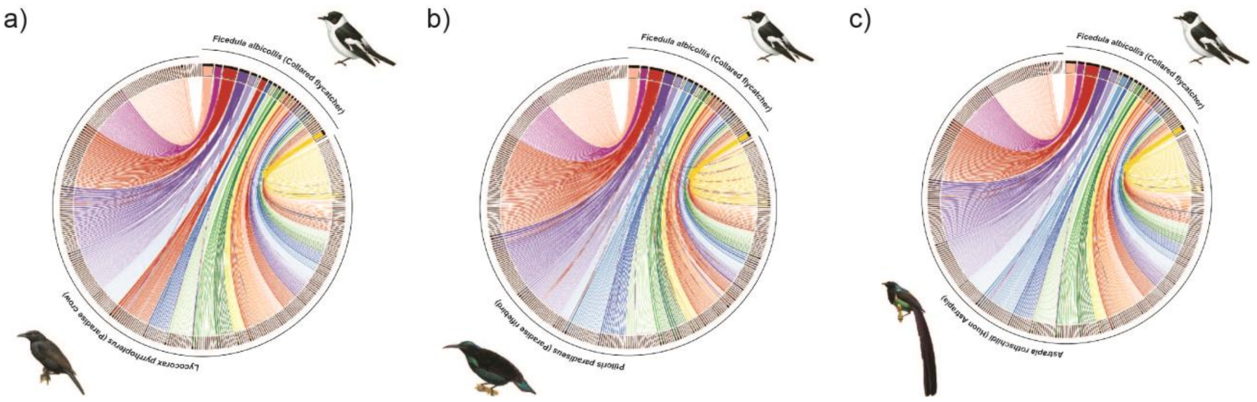

Figure 2: Chromosomal synteny plot between the collared flycatcher and (a) the paradise crow, (b) paradise riflebird and (c) the Huon astrapia. The plot shows scaffolds larger than 50 kb and links (alignments) larger than 2 kb.

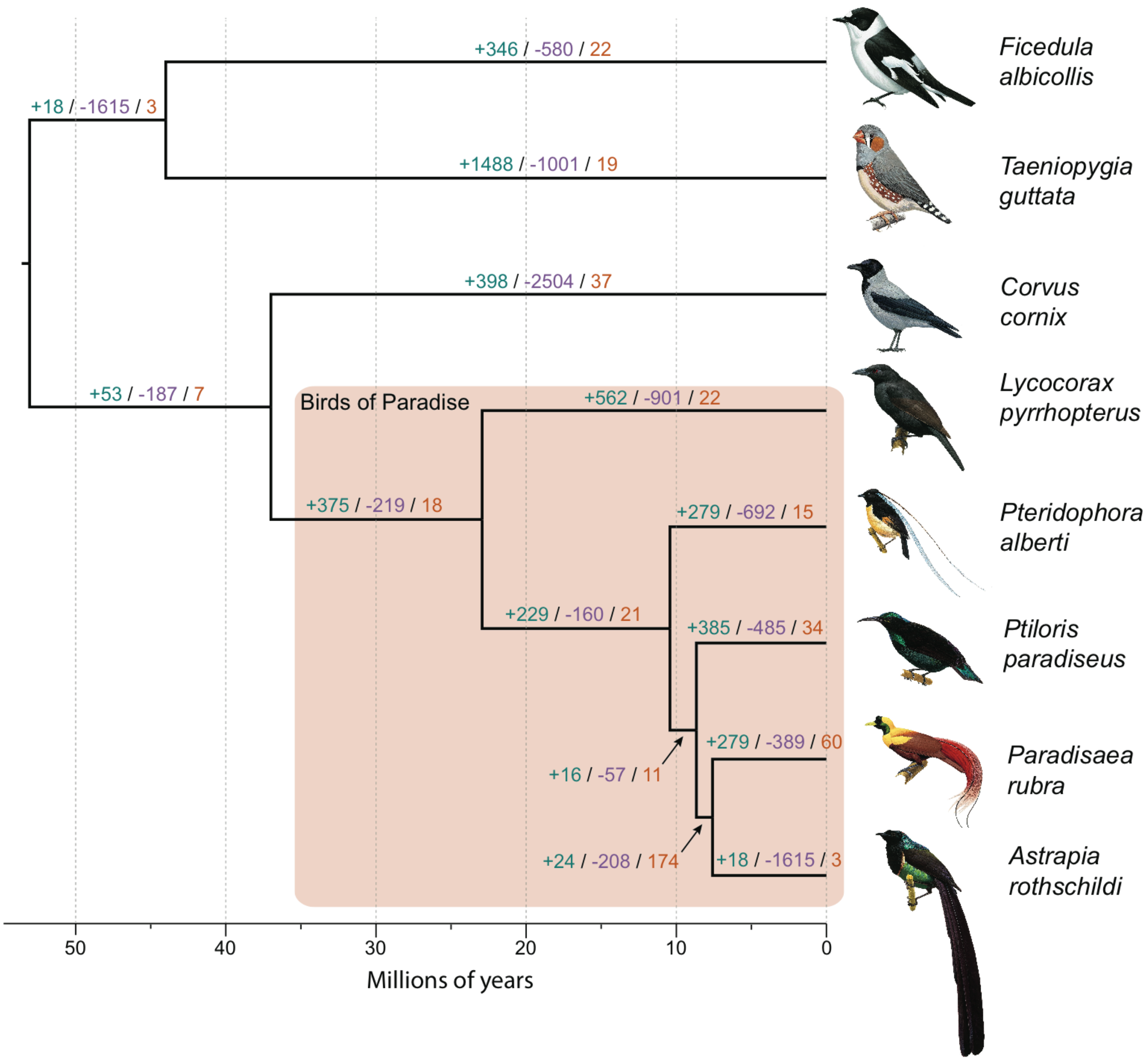

Figure 3: The birds-of-paradise phylogeny. The phylogenetic tree is based on ML and coalescent-based statistical binning of 4,656 genes, and scaled using the divergence times between crow and the birds-of-paradise, and zebra finch and flycatcher (obtained from Timetree.org) as calibration points. Branches are labeled as: # gene family expansions / # gene family contractions / # rapidly evolving gene families.

Tables

Table S1: ***De novo*** **Assembly Statistics.**

	**Number of Scaffolds**	**Scaffold N50**	**Assembly Length**
Astrapia rothschildi	2,081	7.7 Mb	1.03 Gb
Lycocorax pyrrhopterus	3,216	4.2 Mb	1.07 Gb
Ptiloris paradiseus	2,062	4.27 Mb	1.04 Gb

References

1. Gilliard, E.T. (1969). Birds of paradise and bower birds, (American Museum of Natural History).
2. Gill, F., and Donsker, D. (2017). IOC World Bird List (v 7.1). doi: 1014344/IOCML71. doi: 10.14344/IOC. (ML).
3. Frith, C., and Beehler, B. (1998). The Birds of Paradise. (Oxford University Press, Oxford, United Kingdom).
4. Cracraft, J. (1992). The species of the birds-of-paradise (Paradisaeidae): applying the phylogenetic species concept to a complex pattern of diversification. Cladistics *8*, 1-43.
5. Christidis, L., and Schodde, R. (1992). Relationships Among the Birds-of-Paradise (Paradisaeidae) and Bowerbirds (Ptilonorhynchidae)-Protein Evidence. Australian Journal of Zoology *40*, 343-353.
6. Nunn, G.B., and Cracraft, J. (1996). Phylogenetic relationships among the major lineages of the birds-of-paradise (Paradisaeidae) using mitochondrial DNA gene sequences. Molecular Phylogenetics and Evolution *5*, 445-459.
7. Irestedt, M., Jønsson, K.A., Fjeldså, J., Christidis, L., and Ericson, P.G. (2009). An unexpectedly long history of sexual selection in birds-of-paradise. BMC evolutionary biology *9*, 235.
8. Irestedt, M., Batalha-Filho, H., Ericson, P.G., Christidis, L., and Schodde, R. (2017). Phylogeny, biogeography and taxonomic consequences in a bird-of-paradise species complex, Lophorina–Ptiloris (Aves: Paradisaeidae). Zoological Journal of the Linnean Society *181*, 439-470.
9. Scholes, E., and Laman, T.G. (2018). Distinctive courtship phenotype of the Vogelkop Superb Bird-of-Paradise Lophorina niedda Mayr, 1930 confirms new species status. PeerJ *6*, e4621.
10. Fox, H.M., and Ververs, G. (1960). The Nature of Animal Colors., (New York: MacMillan).
11. Fox, D.L. (1976). Animal biochromes and structural colours: physical, chemical, distributional & physiological features of coloured bodies in the animal world, (Univ of California Press).
12. Zi, J., Yu, X., Li, Y., Hu, X., Xu, C., Wang, X., Liu, X., and Fu, R. (2003). Coloration strategies in peacock feathers. Proceedings of the National Academy of Sciences *100*, 12576-12578.
13. Wilts, B.D., Michielsen, K., De Raedt, H., and Stavenga, D.G. (2014). Sparkling feather reflections of a bird-of-paradise explained by finite-difference time-domain modeling. Proceedings of the National Academy of Sciences *111*, 4363-4368.
14. Stavenga, D.G., Leertouwer, H.L., Marshall, N.J., and Osorio, D. (2010). Dramatic colour changes in a bird of paradise caused by uniquely structured breast feather barbules. Proceedings of the Royal Society of London B: Biological Sciences, rspb20102293.
15. Dale, J., Dey, C.J., Delhey, K., Kempenaers, B., and Valcu, M. (2015). The effects of life history and sexual selection on male and female plumage colouration. Nature *527*, 367.
16. Bi, K., Linderoth, T., Vanderpool, D., Good, J.M., Nielsen, R., and Moritz, C. (2013). Unlocking the vault: next-generation museum population genomics. Molecular Ecology *22*, 6018-6032.

17. Poelstra, J.W., Vijay, N., Bossu, C.M., Lantz, H., Ryll, B., Müller, I., Baglione, V., Unneberg, P., Wikelski, M., and Grabherr, M.G. (2014). The genomic landscape underlying phenotypic integrity in the face of gene flow in crows. Science *344*, 1410-1414.
18. Zhan, X., Pan, S., Wang, J., Dixon, A., He, J., Muller, M.G., Ni, P., Hu, L., Liu, Y., and Hou, H. (2013). Peregrine and saker falcon genome sequences provide insights into evolution of a predatory lifestyle. Nature genetics *45*, 563.
19. Zhang, G., Li, C., Li, Q., Li, B., Larkin, D.M., Lee, C., Storz, J.F., Antunes, A., Greenwold, M.J., and Meredith, R.W. (2014). Comparative genomics reveals insights into avian genome evolution and adaptation. Science *346*, 1311-1320.
20. Burga, A., Wang, W., Ben-David, E., Wolf, P.C., Ramey, A.M., Verdugo, C., Lyons, K., Parker, P.G., and Kruglyak, L. (2017). A genetic signature of the evolution of loss of flight in the Galapagos cormorant. Science *356*, eaal3345.
21. Simão, F.A., Waterhouse, R.M., Ioannidis, P., Kriventseva, E.V., and Zdobnov, E.M. (2015). BUSCO: assessing genome assembly and annotation completeness with single-copy orthologs. Bioinformatics *31*, 3210-3212.
22. Kapusta, A., and Suh, A. (2017). Evolution of bird genomes—a transposon's-eye view. Annals of the New York Academy of Sciences *1389*, 164-185.
23. Hillier, L., Miller, W., Birney, E., Warren, W., Hardison, R., Ponting, C., Bork, P., Burt, D., Groenen, M., and Delany, M. (2004). International Chicken Genome Sequencing Consortium: Sequence and comparative analysis of the chicken genome provide unique perspectives on vertebrate evolution. Nature *432*, 69-716.
24. Suh, A., Smeds, L., and Ellegren, H. (2018). Abundant recent activity of retrovirus-like retrotransposons within and among flycatcher species implies a rich source of structural variation in songbird genomes. Molecular Ecology *27*, 99-111.
25. Suh, A. (2016). The phylogenomic forest of bird trees contains a hard polytomy at the root of Neoaves. Zoologica Scripta *45*, 50-62.
26. Jarvis, E.D., Mirarab, S., Aberer, A.J., Li, B., Houde, P., Li, C., Ho, S.Y., Faircloth, B.C., Nabholz, B., and Howard, J.T. (2014). Whole-genome analyses resolve early branches in the tree of life of modern birds. Science *346*, 1320-1331.
27. Prum, R.O., Berv, J.S., Dornburg, A., Field, D.J., Townsend, J.P., Lemmon, E.M., and Lemmon, A.R. (2015). A comprehensive phylogeny of birds (Aves) using targeted next-generation DNA sequencing. Nature *526*, 569.
28. Murrell, B., Weaver, S., Smith, M.D., Wertheim, J.O., Murrell, S., Aylward, A., Eren, K., Pollner, T., Martin, D.P., and Smith, D.M. (2015). Gene-wide identification of episodic selection. Molecular Biology and Evolution *32*, 1365-1371.
29. De Bie, T., Cristianini, N., Demuth, J.P., and Hahn, M.W. (2006). CAFE: a computational tool for the study of gene family evolution. Bioinformatics *22*, 1269-1271.
30. Kawakami, T., Smeds, L., Backström, N., Husby, A., Qvarnström, A., Mugal, C.F., Olason, P., and Ellegren, H. (2014). A high-density linkage map enables a second-generation collared flycatcher genome assembly and reveals the patterns of avian recombination rate variation and chromosomal evolution. Molecular Ecology *23*, 4035-4058.
31. Ellegren, H. (2010). Evolutionary stasis: the stable chromosomes of birds. Trends in Ecology & Evolution *25*, 283-291.
32. Lieberman-Aiden, E., Van Berkum, N.L., Williams, L., Imakaev, M., Ragoczy, T., Telling, A., Amit, I., Lajoie, B.R., Sabo, P.J., and Dorschner, M.O. (2009). Comprehensive

mapping of long-range interactions reveals folding principles of the human genome. Science *326*, 289-293.

33. Peona, V., Weissensteiner, M.H., and Suh, A. (2018). How complete are "complete" genome assemblies?—An avian perspective. Molecular ecology resources.

34. Maddison, W.P., and Knowles, L.L. (2006). Inferring phylogeny despite incomplete lineage sorting. Systematic biology *55*, 21-30.

35. Suh, A., Smeds, L., and Ellegren, H. (2015). The dynamics of incomplete lineage sorting across the ancient adaptive radiation of neoavian birds. PLoS biology *13*, e1002224.

36. Silver, D.L., Hou, L., Somerville, R., Young, M.E., Apte, S.S., and Pavan, W.J. (2008). The secreted metalloprotease ADAMTS20 is required for melanoblast survival. PLoS genetics *4*, e1000003.

37. Bennett, D.C., and Lamoreux, M.L. (2003). The color loci of mice–a genetic century. Pigment Cell & Melanoma Research *16*, 333-344.

38. Noji, S., Koyama, E., Myokai, F., Nohno, T., Ohuchi, H., Nishikawa, K., and Taniguchi, S. (1993). Differential expression of three chick FGF receptor genes, FGFR1, FGFR2 and FGFR3, in limb and feather development. Progress in clinical and biological research *383*, 645-654.

39. Gfrerer, L., Shubinets, V., Hoyos, T., Kong, Y., Nguyen, C., Pietschmann, P., Morton, C.C., Maas, R.L., and Liao, E.C. (2014). Functional analysis of SPECC1L in craniofacial development and oblique facial cleft pathogenesis. Plastic and reconstructive surgery *134*, 748.

40. Li, J., Zhao, X.-L., Gilbert, E.R., Li, D.-Y., Liu, Y.-P., Wang, Y., Zhu, Q., Wang, Y.-G., Chen, Y., and Tian, K. (2014). APOBEC2 mRNA and protein is predominantly expressed in skeletal and cardiac muscles of chickens. Gene *539*, 263-269.

41. Harris, R.S., Bishop, K.N., Sheehy, A.M., Craig, H.M., Petersen-Mahrt, S.K., Watt, I.N., Neuberger, M.S., and Malim, M.H. (2003). DNA deamination mediates innate immunity to retroviral infection. Cell *113*, 803-809.

42. Mangeat, B., Turelli, P., Caron, G., Friedli, M., Perrin, L., and Trono, D. (2003). Broad antiretroviral defence by human APOBEC3G through lethal editing of nascent reverse transcripts. Nature *424*, 99.

43. Knisbacher, B.A., and Levanon, E.Y. (2015). DNA editing of LTR retrotransposons reveals the impact of APOBECs on vertebrate genomes. Molecular Biology and Evolution *33*, 554-567.

44. Lind, O., Henze, M.J., Kelber, A., and Osorio, D. (2017). Coevolution of coloration and colour vision? Phil. Trans. R. Soc. B *372*, 20160338.

45. Price, T.D. (2017). Sensory drive, color, and color vision. The American Naturalist *190*, 157-170.

46. Mundy, N.I., Stapley, J., Bennison, C., Tucker, R., Twyman, H., Kim, K.-W., Burke, T., Birkhead, T.R., Andersson, S., and Slate, J. (2016). Red carotenoid coloration in the zebra finch is controlled by a cytochrome P450 gene cluster. Current Biology *26*, 1435-1440.

47. Bloch, N.I. (2015). Evolution of opsin expression in birds driven by sexual selection and habitat. Proceedings of the Royal Society of London B: Biological Sciences *282*, 20142321.

48. Bowers, J.M., and Alexander, B.K. (1967). Mice: individual recognition by olfactory cues. Science *158*, 1208-1210.

49. Ables, E.M., Kay, L.M., and Mateo, J.M. (2007). Rats assess degree of relatedness from human odors. Physiology & behavior *90*, 726-732.
50. Smith, T.E., Tomlinson, A.J., Mlotkiewicz, J.A., and Abbott, D.H. (2001). Female marmoset monkeys (Callithrix jacchus) can be identified from the chemical composition of their scent marks. Chemical senses *26*, 449-458.
51. Johansson, B.G., and Jones, T.M. (2007). The role of chemical communication in mate choice. Biological Reviews *82*, 265-289.
52. Roper, T.J. (1999). Olfaction in birds. Advances in the Study of Behavior *28*, 247-247.
53. Khan, I., Yang, Z., Maldonado, E., Li, C., Zhang, G., Gilbert, M.T.P., Jarvis, E.D., O'Brien, S.J., Johnson, W.E., and Antunes, A. (2015). Olfactory receptor subgenomes linked with broad ecological adaptations in Sauropsida. Molecular Biology and Evolution *32*, 2832-2843.
54. Yokosuka, M., Hagiwara, A., Saito, T.R., Tsukahara, N., Aoyama, M., Wakabayashi, Y., Sugita, S., and Ichikawa, M. (2009). Histological properties of the nasal cavity and olfactory bulb of the Japanese jungle crow Corvus macrorhynchos. Chemical senses *34*, 581-593.
55. Irestedt, M., Ohlson, J.I., Zuccon, D., Källersjö, M., and Ericson, P.G. (2006). Nuclear DNA from old collections of avian study skins reveals the evolutionary history of the Old World suboscines (Aves, Passeriformes). Zoologica Scripta *35*, 567-580.
56. Meyer, M., and Kircher, M. (2010). Illumina sequencing library preparation for highly multiplexed target capture and sequencing. Cold Spring Harbor Protocols *2010*, pdb. prot5448.
57. Andrews, S. (2010). FASTQC. A quality control tool for high throughput sequence data. URL http://www.bioinformatics.babraham.ac.uk/projects/fastqc.
58. Simpson, J.T. (2013). Exploring Genome Characteristics and Sequence Quality Without a Reference. arXiv preprint arXiv:1307.8026.
59. Simpson, J.T., and Durbin, R. (2012). Efficient de novo assembly of large genomes using compressed data structures. Genome Research *22*, 549-556.
60. Gnerre, S., MacCallum, I., Przybylski, D., Ribeiro, F.J., Burton, J.N., Walker, B.J., Sharpe, T., Hall, G., Shea, T.P., and Sykes, S. (2011). High-quality draft assemblies of mammalian genomes from massively parallel sequence data. Proceedings of the National Academy of Sciences *108*, 1513-1518.
61. Luo, R., Liu, B., Xie, Y., Li, Z., Huang, W., Yuan, J., He, G., Chen, Y., Pan, Q., and Liu, Y. (2012). SOAPdenovo2: an empirically improved memory-efficient short-read de novo assembler. GigaScience *1*, 18.
62. Parra, G., Bradnam, K., and Korf, I. (2007). CEGMA: a pipeline to accurately annotate core genes in eukaryotic genomes. Bioinformatics *23*, 1061-1067.
63. Li, H., and Durbin, R. (2009). Fast and accurate short read alignment with Burrows–Wheeler transform. Bioinformatics *25*, 1754-1760.
64. Li, H., Handsaker, B., Wysoker, A., Fennell, T., Ruan, J., Homer, N., Marth, G., Abecasis, G., and Durbin, R. (2009). The sequence alignment/map format and SAMtools. Bioinformatics *25*, 2078-2079.
65. McKenna, A., Hanna, M., Banks, E., Sivachenko, A., Cibulskis, K., Kernytsky, A., Garimella, K., Altshuler, D., Gabriel, S., and Daly, M. (2010). The Genome Analysis Toolkit: a MapReduce framework for analyzing next-generation DNA sequencing data. Genome Research *20*, 1297-1303.

66. Korneliussen, T.S., Albrechtsen, A., and Nielsen, R. (2014). ANGSD: analysis of next generation sequencing data. BMC bioinformatics *15*, 356.
67. Smit, A., Hubley, R., and Green, P. (2014). RepeatModeler Open-1.0. 2008-2010.
68. Bao, Z., and Eddy, S.R. (2002). Automated de novo identification of repeat sequence families in sequenced genomes. Genome Research *12*, 1269-1276.
69. Price, A.L., Jones, N.C., and Pevzner, P.A. (2005). De novo identification of repeat families in large genomes. Bioinformatics *21*, i351-i358.
70. Benson, G. (1999). Tandem repeats finder: a program to analyze DNA sequences. Nucleic Acids Research *27*, 573.
71. Jurka, J., Kapitonov, V.V., Pavlicek, A., Klonowski, P., Kohany, O., and Walichiewicz, J. (2005). Repbase Update, a database of eukaryotic repetitive elements. Cytogenetic and genome research *110*, 462-467.
72. Smeds, L., Warmuth, V., Bolivar, P., Uebbing, S., Burri, R., Suh, A., Nater, A., Bureš, S., Garamszegi, L.Z., and Hogner, S. (2015). Evolutionary analysis of the female-specific avian W chromosome. Nature communications *6*, 7330.
73. Vijay, N., Bossu, C.M., Poelstra, J.W., Weissensteiner, M.H., Suh, A., Kryukov, A.P., and Wolf, J.B. (2016). Evolution of heterogeneous genome differentiation across multiple contact zones in a crow species complex. Nature communications *7*, 13195.
74. Platt, R.N., Blanco-Berdugo, L., and Ray, D.A. (2016). Accurate transposable element annotation is vital when analyzing new genome assemblies. Genome biology and evolution *8*, 403-410.
75. Lavoie, C.A., Platt, R.N., Novick, P.A., Counterman, B.A., and Ray, D.A. (2013). Transposable element evolution in Heliconius suggests genome diversity within Lepidoptera. Mobile DNA *4*, 21.
76. Altschul, S.F., Madden, T.L., Schäffer, A.A., Zhang, J., Zhang, Z., Miller, W., and Lipman, D.J. (1997). Gapped BLAST and PSI-BLAST: a new generation of protein database search programs. Nucleic Acids Research *25*, 3389-3402.
77. Katoh, K., and Standley, D.M. (2013). MAFFT multiple sequence alignment software version 7: improvements in performance and usability. Molecular Biology and Evolution *30*, 772-780.
78. Smit, A., Hubley, R., and Green, P. 1996–2010. RepeatMasker Open-3.0.
79. Grabherr, M.G., Russell, P., Meyer, M., Mauceli, E., Alföldi, J., Di Palma, F., and Lindblad-Toh, K. (2010). Genome-wide synteny through highly sensitive sequence alignment: Satsuma. Bioinformatics *26*, 1145-1151.
80. Krzywinski, M., Schein, J., Birol, I., Connors, J., Gascoyne, R., Horsman, D., Jones, S.J., and Marra, M.A. (2009). Circos: an information aesthetic for comparative genomics. Genome Research *19*, 1639-1645.
81. Holt, C., and Yandell, M. (2011). MAKER2: an annotation pipeline and genome-database management tool for second-generation genome projects. BMC bioinformatics *12*, 491.
82. Korf, I. (2004). Gene finding in novel genomes. BMC bioinformatics *5*, 59.
83. Stanke, M., and Waack, S. (2003). Gene prediction with a hidden Markov model and a new intron submodel. Bioinformatics *19*, ii215-ii225.
84. Lechner, M., Hernandez-Rosales, M., Doerr, D., Wieseke, N., Thévenin, A., Stoye, J., Hartmann, R.K., Prohaska, S.J., and Stadler, P.F. (2014). Orthology detection combining clustering and synteny for very large datasets. PLoS one *9*, e105015.

85. Warren, W.C., Clayton, D.F., Ellegren, H., Arnold, A.P., Hillier, L.W., Künstner, A., Searle, S., White, S., Vilella, A.J., and Fairley, S. (2010). The genome of a songbird. Nature *464*, 757.
86. Löytynoja, A., and Goldman, N. (2005). An algorithm for progressive multiple alignment of sequences with insertions. Proceedings of the National Academy of Sciences of the United States of America *102*, 10557-10562.
87. Kück, P., Meusemann, K., Dambach, J., Thormann, B., von Reumont, B.M., Wägele, J.W., and Misof, B. (2010). Parametric and non-parametric masking of randomness in sequence alignments can be improved and leads to better resolved trees. Frontiers in zoology *7*, 1.
88. Kück, P. (2009). ALICUT: a Perlscript which cuts ALISCORE identified RSS. Department of Bioinformatics, Zoologisches Forschungsmuseum A. Koenig (ZFMK), Bonn, Germany, version *2*.
89. Stamatakis, A. (2014). RAxML version 8: a tool for phylogenetic analysis and post-analysis of large phylogenies. Bioinformatics, btu033.
90. Mirarab, S., Bayzid, M.S., Boussau, B., and Warnow, T. (2014). Statistical binning enables an accurate coalescent-based estimation of the avian tree. Science *346*, 1250463.
91. Mirarab, S., Reaz, R., Bayzid, M.S., Zimmermann, T., Swenson, M.S., and Warnow, T. (2014). ASTRAL: genome-scale coalescent-based species tree estimation. Bioinformatics *30*, i541-i548.
92. Kozlov, A.M., Aberer, A.J., and Stamatakis, A. (2015). ExaML version 3: a tool for phylogenomic analyses on supercomputers. Bioinformatics *31*, 2577-2579.
93. Sanderson, M.J. (2003). r8s: inferring absolute rates of molecular evolution and divergence times in the absence of a molecular clock. Bioinformatics *19*, 301-302.
94. Yang, Z. (2007). PAML 4: phylogenetic analysis by maximum likelihood. Molecular Biology and Evolution *24*, 1586-1591.
95. Pond, S.L.K., and Muse, S.V. (2005). HyPhy: hypothesis testing using phylogenies. In Statistical methods in molecular evolution. (Springer), pp. 125-181.
96. Benjamini, Y., and Hochberg, Y. (1995). Controlling the false discovery rate: a practical and powerful approach to multiple testing. Journal of the royal statistical society. Series B (Methodological), 289-300.
97. Mudunuri, U., Che, A., Yi, M., and Stephens, R.M. (2009). bioDBnet: the biological database network. Bioinformatics *25*, 555-556.
98. Zerbino, D.R., Achuthan, P., Akanni, W., Amode, M.R., Barrell, D., Bhai, J., Billis, K., Cummins, C., Gall, A., and Girón, C.G. (2017). Ensembl 2018. Nucleic Acids Research *46*, D754-D761.
99. Enright, A.J., Van Dongen, S., and Ouzounis, C.A. (2002). An efficient algorithm for large-scale detection of protein families. Nucleic Acids Research *30*, 1575-1584.
100. Hedges, S.B., Dudley, J., and Kumar, S. (2006). TimeTree: a public knowledge-base of divergence times among organisms. Bioinformatics *22*, 2971-2972.
101. Han, M.V., Thomas, G.W., Lugo-Martinez, J., and Hahn, M.W. (2013). Estimating gene gain and loss rates in the presence of error in genome assembly and annotation using CAFE 3. Molecular Biology and Evolution *30*, 1987-1997.
102. Prost, S., Armstrong, EE., Nylander, J., Thomas, GWC., Suh, A., Petersen, B., Dalen, L., Benz, BW., Blom, MPK., Palkopoulou, E., et al. Supporting data for "Comparative anal-

yses identify genomic features potentially involved in the evolution of birds-of-paradise.". GigaScience Database 2019. http://dx.doi.org/10.5524/100521

103. The Birds-of-Paradise CAFE page. https://cafe-portal.gitlab.io/bop/main.html accessed 10 Jan. 2019.

Supplementary

Supplementary Figures

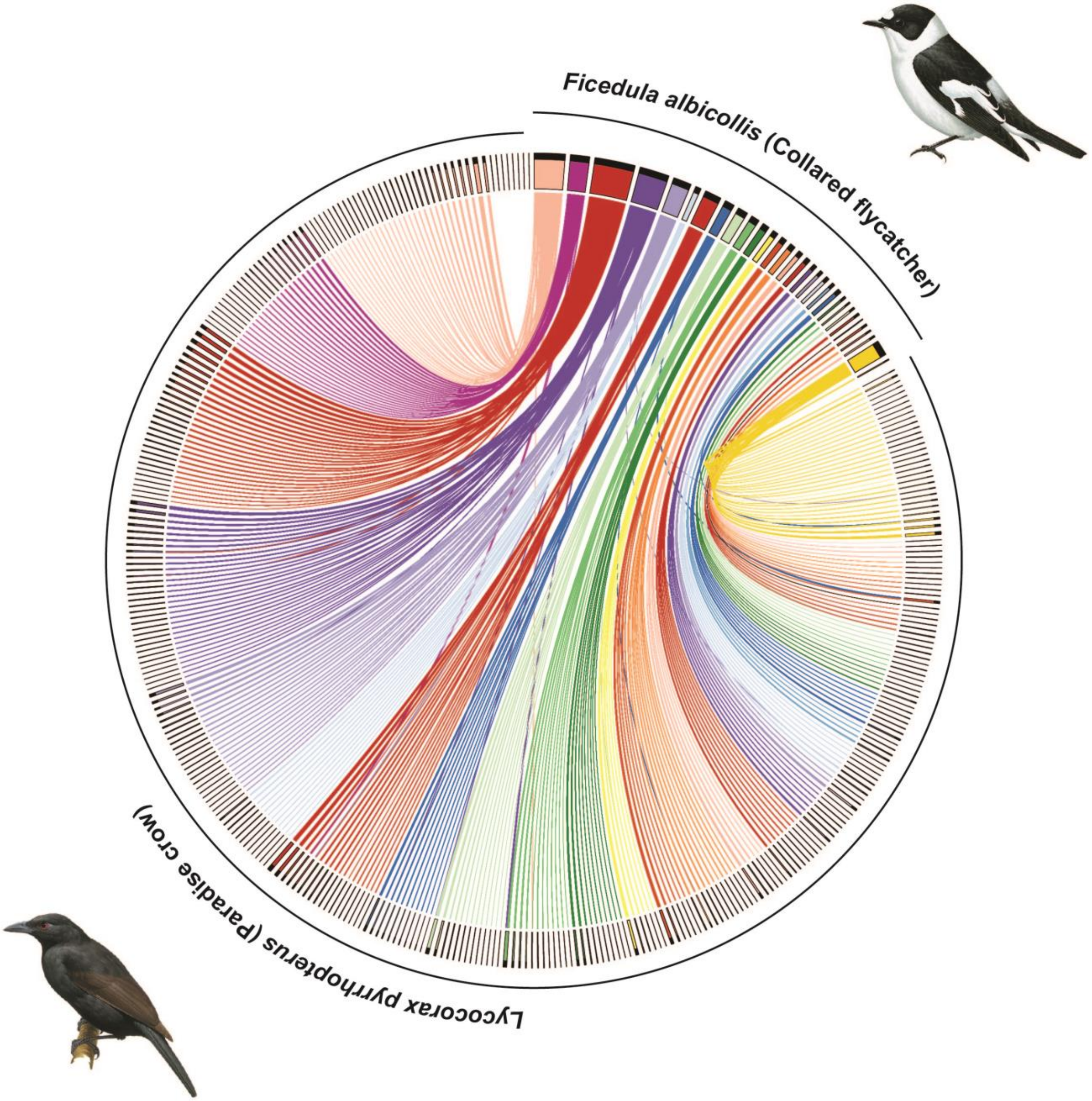

Supplementary Figure S1: Chromosomal synteny plot between the collared flycatcher and the paradise crow. The plot shows scaffolds larger than 50 kb and links (alignments) larger than 2 kb.

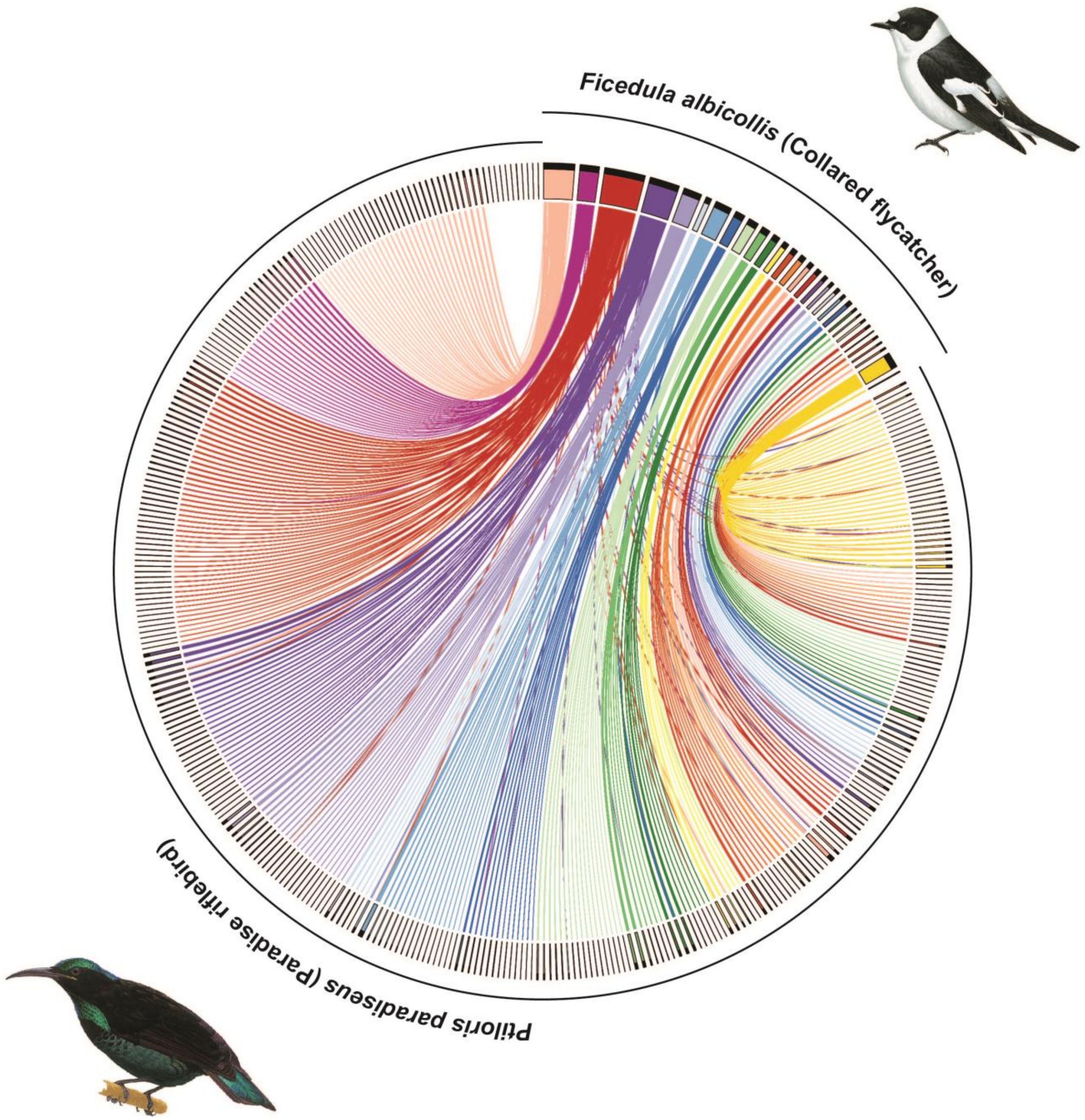

Supplementary Figure S2: Chromosomal synteny plot between the collared flycatcher and the paradise riflebird. The plot shows scaffolds larger than 50 kb and links (alignments) larger than 2 kb.

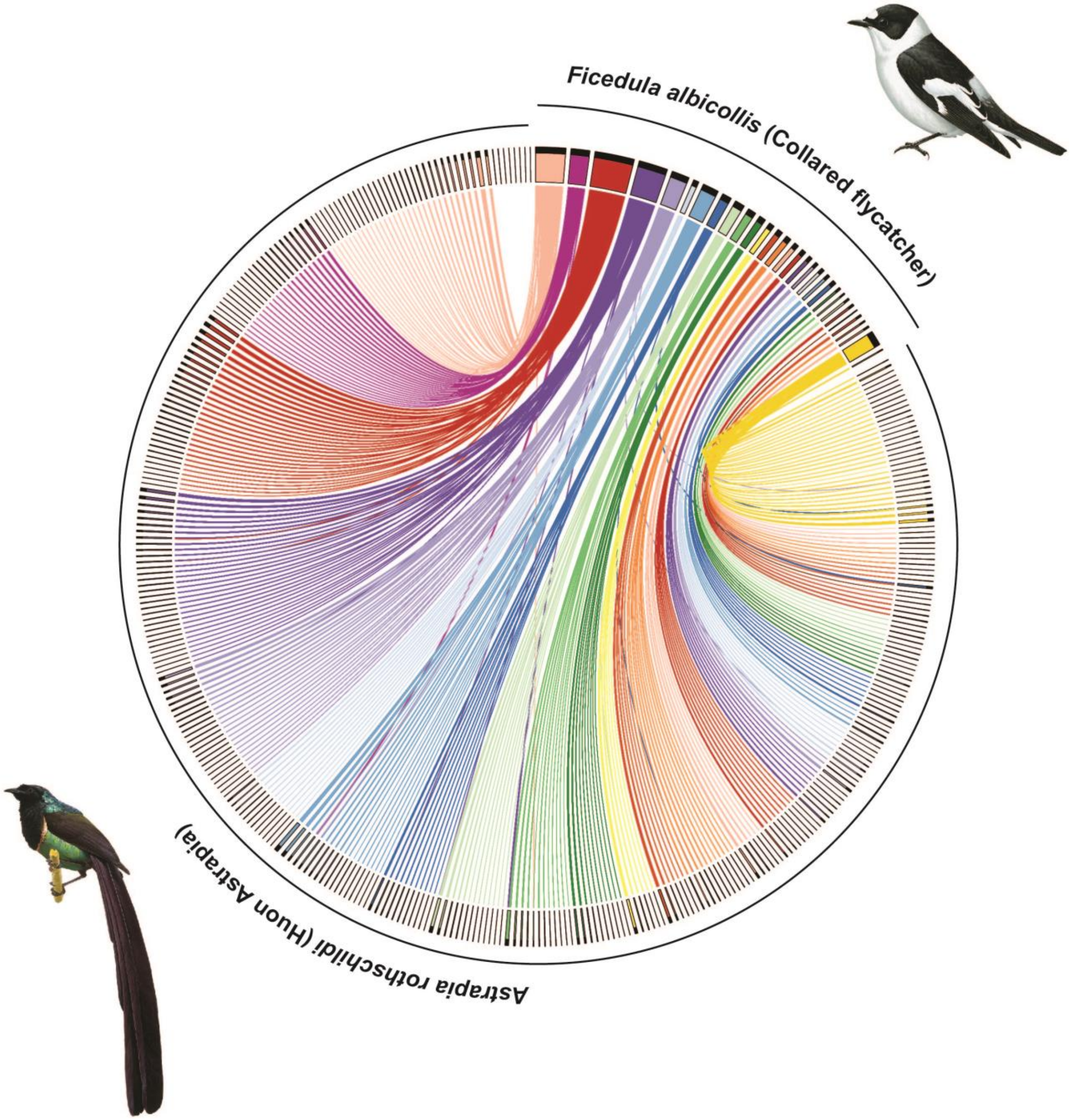

Supplementary Figure S3: Chromosomal synteny plot between the collared flycatcher and the Huon Astrapia. The plot shows scaffolds larger than 50 kb and links (alignments) larger than 2 kb.

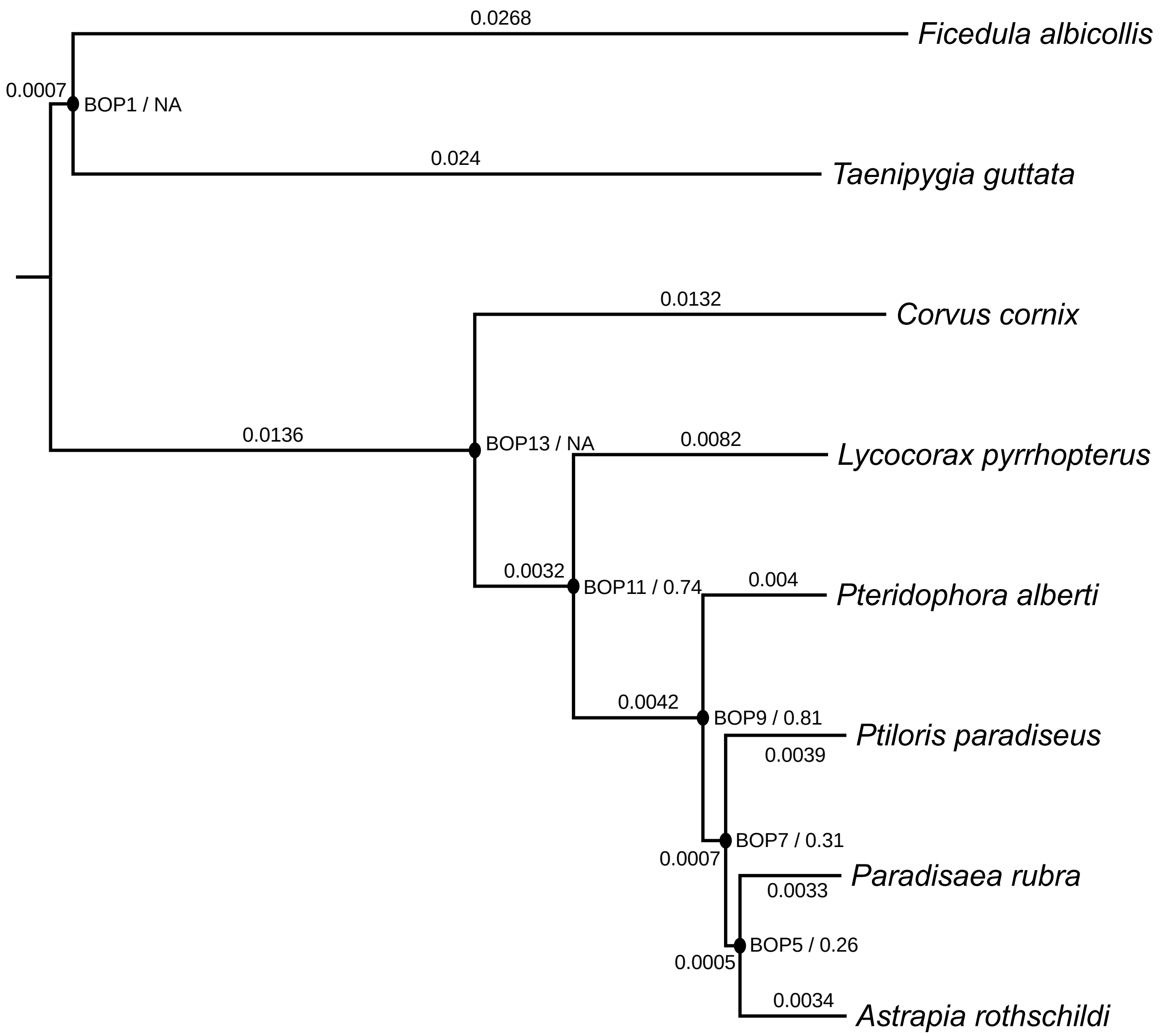

Supplementary Figure S4: Phylogenetic species tree. The species tree was reconstructed from individual maximum likelihood-based gene trees using 4,656 exons and coalescent-based statistical binning (Astral). Branch lengths are depicted on the branches (calculated via a ML tree constructed using ExaML and 200 randomly selected genes). Nodes are labeled and concordance factor is shown next to the node labels (i.e. [node label] / [concordance factor]). All nodes have 100 bootstrap support.

Supplementary Tables

Supplementary Table S1: BUSCO scores. Scores were calculated using Busco2 and the aves_odb9 data set (4,915 genes total).

	**Complete**	**Duplicated**	**Fragmented**	**Missing**
Astrapia rothschildi	4,669 (95.0%)	48 (1.0%)	139 (2.8%)	107 (2.2%)
Lycocorax pyrrhopterus	4,659 (93.8%)	51 (1.0%)	161 (3.3%)	95 (1.9%)
Ptiloris paradiseus	4,675 (95.1%)	44 (0.9%)	135 (2.7%)	105 (2.2%)
Paradisaea rubra	4,662 (94.9%)	47 (1.0%)	150 (3.1%)	103 (2.0%)
Pteridophora alberti	4,661 (94.8%)	39 (0.8%)	155 (3.2%)	99 (2.0%)

Supplementary Table S2: Gene annotation.

	# of Transcripts	Average transcript size (bp)	Average introns size (kb, rounded)	Average # of introns per gene
Astrapia rothschildi	16,260	1,603	2.2	9
Lycocorax pyrrhopterus	17,023	1,572	2.2	9
Ptiloris paradiseus	17,269	1,584	2.2	9
Paradisaea rubra	16,822	1,561	2.2	9
Pteridophora alberti	16,721	1,562	2.2	9

Supplementary Table S3: RepeatMasker annotation of the three birds-of-paradise genome assemblies using a library of our *de novo* repeat annotations of birds-of-paradise merged with existing avian repeat libraries.

	Astrapia rothschildi			*Lycocorax pyrrhopterus*			*Ptiloris paradisaeus*		
**Repeat type**	**Copies**	**Total bp**	**Total %**	**Copies**	**Total bp**	**Total %**	**Copies**	**Total bp**	**Total %**
**SINE**	8,019	966,992	0.09	7,974	955,190	0.09	7,977	961,945	0.09
**LINE**	128,473	38,885,201	3.67	130,706	40,271,136	3.76	129,094	38,994,767	3.68
**LTR**	38,693	20,692,445	1.95	48,395	27,819,221	2.60	39,123	21,311,765	2.01
**DNA**	4,582	790,017	0.07	4,734	845,120	0.08	4,617	790,421	0.07
**Unclassified**	34,049	9,005,494	0.85	37,519	9,167,482	0.86	30,814	8,931,323	0.84
**Total inter- ed repeats**	213,816	70,340,149	6.63	229,328	79,058,149	7.39	211,625	70,990,221	6.69
**Small RNA**	538	46,523	0.00	577	50,738	0.00	546	47,744	0.00
**Satellites**	2,884	623,756	0.06	2,706	572,161	0.05	2,855	646,354	0.06
**Simple re-**	195,600	9,348,199	0.88	193,765	9,101,884	0.85	197,648	9,318,098	0.88
**Low com- y**	43,076	2,388,544	0.23	42,067	2,292,784	0.21	42,546	2,360,570	0.22
**Total tandem ts**	242,098	12,407,022	1.17	239,115	12,017,567	1.11	243,595	12,372,766	1.16
**Total repeats**	455,914	82,747,171	7.80	468,443	91,075,716	8.50	455,220	83,362,987	7.85
**Assembly**		1.06Gb			1.07Gb			1.06Gb	
**Gap ('N') bp**		13,196,877			10,466,138			10,993,394	

Supplementary Table S4: Characteristics of the manually curated TE consensus sequences from *Lycocorax pyrrhopterus*, including lineage-specific LTR families termed as 'lycPyrLTR*'.

Class	Sub-class	Super-	Family	Subfamily	Similarity to known repeats	Consen- atus	Consensus	TSD
Retrotransposon	LTR	ERV1	lycPyrLTR1	lycPyrLTR1	None	Complete	463	4 bp
Retrotransposon	LTR	ERV1	lycPyrLTR2	lycPyrLTR2	None	Complete	535	4 bp
Retrotransposon	LTR	ERV1	lycPyrLTR3	lycPyrLTR3	None	Complete	623	4 bp
Retrotransposon	LTR	ERV1	TguERV3	TguERV3_LTR2b-L_lycPyr	Partially TguERV3_LTR2b (68% similarity)	Complete	601	4 bp
Retrotransposon	LTR	ERV1	TguERV1	TguERV1_LTR1a-L_lycPyr	Partially TguERV1_LTR1a (72% similarity)	Complete	600	4 bp
Retrotransposon	LTR	ERV1	TguLTR11	TguLTR11l-L_lycPyr.inc	Partially TguLTR11l + TguERV2_I + TguERV1_I + TguERV3_I (79% + 65% + 63% + 66% similarity)	ncomplete nd	4535	?
Retrotransposon	LTR	ERV1	TguLTR12	TguLTR12-L_lycPyr.inc	Partially TguLTR12 (84% similarity)	Incom- TSD	625	?
Retrotransposon	LTR	ERV2	lycPyrLTRK1	lycPyrLTRK1a	None	Complete	366	6 bp
Retrotransposon	LTR	ERV2	lycPyrLTRK1	lycPyrLTRK1b	None	Complete	366	6 bp
Retrotransposon	LTR	ERV2	lycPyrLTRK2	lycPyrLTRK2	None	Complete	647	6 bp
Retrotransposon	LTR	ERV2	lycPyrLTRK3	lycPyrLTRK3a	None	Complete	689	6 bp
Retrotransposon	LTR	ERV2	lycPyrLTRK3	lycPyrLTRK3b	None	Complete	744	6 bp
Retrotransposon	LTR	ERV2	lycPyrLTRK4	lycPyrLTRK4	None	Complete	605	6 bp
Retrotransposon	LTR	ERV2	lycPyrLTRK5	lycPyrLTRK5	None	Complete	397	6 bp

Retrotransposon	LTR	ERV2	lycPyrLTRK6	lycPyrLTRK6	None	Complete	666	6 bp
Retrotransposon	LTR	ERV2	lycPyrLTRK7	lycPyrLTRK7	None	Complete	334	6 bp
Retrotransposon	LTR	ERV2	lycPyrLTRK8	lycPyrLTRK8	None	Complete	408	6 bp
Retrotransposon	LTR	ERV2	lycPyrLTRK9	lycPyrLTRK9_LTR	None	Complete	380	6 bp
Retrotransposon	LTR	ERV2	lycPyrLTRK9	lycPyrLTRK9_I.inc	None	[illegible]complete end	537	6 bp
Retrotransposon	LTR	ERV3	lycPyrLTRL1	lycPyrLTRL1	None	Complete	1171	5 bp
Retrotransposon	LTR	ERV3	lycPyrLTRL2	lycPyrLTRL2	None	Complete	1105	5 bp
Retrotransposon	LTR	ERV3	lycPyrLTRL3	lycPyrLTRL3	None	Complete	460	5 bp
Retrotransposon	LTR	ERV3	lycPyrLTRL4	lycPyrLTRL4	None	Complete	807	5 bp
Retrotransposon	LTR	ERV3	lycPyrLTRL5	lycPyrLTRL5	None	Complete	1281	5 bp
Retrotransposon	LTR	ERV3	lycPyrLTRL6	lycPyrLTRL6	None	Complete	670	5 bp
Retrotransposon	LTR	ERV3	lycPyrLTRL7	lycPyrLTRL7.inc	Partially Tgu_rep3 (80% similarity)	Incom-[illegible]ete 3' end	177	?
Retrotransposon	LTR	ERV3	TguERVL2	TguERVL2b-LTR-L_lycPyr	Partially TguERVL2b3_LTR + TguERVL2b1_LTR (85% + 79% similarity)	Complete	579	5 bp
Retrotransposon	LTR	ERV3	TguERVL2	TguERVL2a2-LTR-L_lycPyr	Partially TguERVL2a2-LTR (93% similarity)	Complete	941	5 bp
Retrotransposon	LTR	ERV3	TguLTRL1	TguLTRL1-La_lycPyr	Partially TguL-TRL1a7 (75% similarity)	Complete	647	5 bp

Retrotransposon	LTR	ERV3	TguLTRL1	TguLTRL1-Lb_lycPyr.inc	Partially TguERVL1_I + TguLTRL1a6 + TguLTRL1a7 (88% + 77% + 74% similarity)	Incom- ' end	2729	?
Retrotransposon	LTR	ERV3	TguLTRL1	TguLTRL1-Lc_lycPyr.inc	Partially TguERVL1_I + TguERVL2_I + TguLTRL6b (80% + 78% + 97% similarity)	Incom- ' and 3'	1754	?
Retrotransposon	LTR	ERV3	TguLTRL1	TguLTRL1-Ld_lycPyr.inc	Partially TguL-TRL1a6 + TguL-TRL1a7 + TguL-TRL1_I (77% + 75% + 75% simi-larity)	Incom- ' end	2655	?
Retrotransposon	LTR	ERV3	TguLTRL1	TguLTRL1-Le_lycPyr.inc	Partially TguL-TRL1a7 + TguERVL1_I (75% + 77% similarity)	Incom- ' and 3'	3079	?
Unknown	Un-	Un-	Unknown	lycPyr5-275.3inc	None	Incom- ' end	177	?
Unknown	Un-	Un-	Unknown	lycPyr5-1942.inc	None	Incom- ' and 3'	620	?
Unknown	Un-	Un-	Unknown	lycPyr6-947.inc_sat	None	Incom- ' and 3'	3602	?

Supplementary Table S5: Top 10 gene tree topology counts (423 total topologies in 4,450 rooted gene trees). Average Robinson-Foulds distance for all 4,656 gene trees is 3.92. Z: zebra finch; F: collared flycatcher; C: hooded crow; L: *Lycocorax*; Pte: *Pteridophora*; Pti: *Ptiloris*; Par: *Paradisaea*; A: *Astrapia*.

Topology	Count
((Z,F),(C,(L,(Pte,(Pti,(Par,A))))))	430
((Z,F),(C,(L,(Pte,((Pti,Par),A)))))	357
((Z,F),(C,(L,(Pte,(Par,(Pti,A))))))	279
((Z,F),(C,(L,(Pti,(Pte,(Par,A))))))	224
((Z,F),(C,(L,((Pti,Par),(Pte,A)))))	167
((Z,F),(C,(L,(Pti,((Pte,Par),A)))))	166
((Z,F),(C,(L,(((Pti,Par),Pte),A))))	162
((Z,F),(C,(L,((Pte,Pti),(Par,A)))))	161
((Z,F),(C,(L,((Pti,(Pte,Par)),A))))	159
((Z,F),(C,(L,(Pti,(Par,(Pte,A))))))	156

Supplementary Table S6: Saturation Analysis. Pairwise dN/dS ratio.

**Astrapia**							
**Corvus**	0.036						
**Ficedula**	0.046	0.063					
**Taeniopygia**	0.044	0.059	0.046				
**Lycocorax**	0.014	0.037	0.046	0.044			
**Paradisaea**	0.006	0.034	0.046	0.043	0.014		
**Pteridophora**	0.007	0.036	0.046	0.044	0.014	0.007	
**Ptiloris**	0.006	0.036	0.046	0.044	0.014	0.006	0.007

Supplementary Table S7. Genes under positive selection. Gene symbols in bold mark genes significant after multiple-testing correction using FDR (<0.05 cut-off).

GenBank Accession	Gene Symbol
XM_016302299.1	**RSPH14**
XM_005060676.1	SNX18
XM_005061576.2	RSG1
XM_016299097.1	COL4A1
XM_016304880.1	MCTP1
XM_016303427.1	**FGD6**
XM_005042552.1	NDRG1
XM_005062657.1	MRPL34
XM_005057187.1	BPIFB2
XM_010403165.3	C8orf48
XM_016298377.1	LOC101809528
XM_005057074.2	LOC101821424
XM_010401845.3	**camC**
XM_016301269.1	LOC101807976
XM_016302077.1	TRAFD1
XM_005048141.1	SH2D4B
XM_005044853.1	MGARP
XM_005039384.1	ADAMTS20
XM_005037072.1	TAF10
XM_005054309.2	C14H16orf71
XM_005056583.1	EVI2A
-	guaA
XM_005038551.2	CCDC181
XM_016298245.1	COL4A5
XM_005059334.2	LAD1
XM_016301828.1	LOC101813372
XM_019280702.1	**act-2b**
XM_016296212.1	AGAP3
XM_005051230.1	FETUB
XM_005043410.1	POLH
XM_004175487.1	**CORIN**
XM_019282258.2	**Slc30a10**
XM_005054364.2	**DRC3**
-	**PRP5**

XM_016300815.1	ZWILCH
XM_005043438.1	WDR27
XM_005058894.2	S100A11
XM_005046922.2	C5H11orf74
XM_016306090.1	SLC9A2
XM_005050076.2	LOC101808676
XM_016300582.1	ATP7B
XM_005051988.2	**TCF12**
XM_016303963.1	LOC107604184
XM_005061593.1	LOC101821569
XM_016298934.1	ITPK1
XM_016302041.1	CARHSP1
XM_005049042.2	LOC101807582
XM_016303572.1	IDO2
XM_005052727.1	LOC101813437
XM_005047366.2	GPATCH2L
XM_005057073.2	WISP2
XM_016303591.1	**ADD2**
XM_005055863.2	PMP22
XM_005062304.2	IGSF21
XM_016305344.1	**LOC101812147**
XM_005050061.1	CABP4
XM_016298968.1	LOC101816855
XM_005056673.2	LOC101817428
XM_005056783.1	CBX2
XM_005056335.1	PTDSS1
XM_005042325.2	BARHL1
XM_005055795.1	HABP2
XM_005048773.2	SYTL1
XM_016303687.1	PAQR7
XM_016303706.1	CCDC89
XM_016301660.1	KIF3C
XM_016305278.1	PHLDA3
XM_005059500.2	PHLDA1
XM_016303529.1	**LOC101807907**
XM_016299105.1	GPX2
XM_005044014.1	**TPBG**
XM_005049721.1	LOC101813208
XM_005039251.1	RNASEL
XM_005049817.2	GPR88
XM_005050542.1	NEXMIF
XM_005045976.1	LOC101813871
XM_005061804.2	MOB3C
XM_005050079.2	ITPKB
XM_005043012.2	LOC101811548
XM_005061100.2	SLC12A3
XM_005052526.1	LOC101822159
XM_005060823.1	DOCK8
XM_005060810.2	SLC7A2
XM_016298010.1	G6PC2
XM_016299897.1	ZEB1
XM_005040638.2	CCDC149
XM_005045593.1	ALAD
XM_005055395.2	SPAG16
XM_005048989.2	HAUS1
XM_016299631.1	**NCKAP1**
XM_005060340.1	SLC25A27
XM_005037255.2	**Atp6ap2**
XM_005044316.2	PTPN11
XM_005055265.2	SLC25A10
XM_005056255.2	LOC101816285
XM_016298524.1	TSPAN9
XM_005061480.2	TCAF2

XM_005061498.2	ANXA11
XM_016299390.1	TAF11
XM_005059540.1	NCLN
XM_016304488.1	NR2E3
XM_010400678.3	**MAML2**
XM_005051635.1	P3H4
XM_005059699.2	GAB2
XM_005044075.1	**Lyrm2**
XM_005038313.1	AGL
XM_016300131.1	TERT
XM_005058028.2	**PPP2R2A**
XM_016296086.1	FGFR1
XM_016303624.1	GCN1
XM_005040177.1	**PARVB**
XM_005055228.2	LIMS1
XM_005037507.2	PAFAH1B2
XM_005058597.2	LOC101821368
XM_005060625.1	UBE2Q1
XM_005059102.1	MZT1
XM_016297193.1	B3GLCT
XM_005038138.1	PDS5B
XM_005038022.1	LMO4
XM_005049530.2	**asic4**
XM_016300038.1	RBP2
XM_005050724.1	CYFIP2
XM_010399761.3	**Pdgfrb**
XM_005053530.2	STRA8
XM_005039572.1	TAF4B
XM_016300054.1	**SERBP1**
XM_005041939.2	**COLEC12**
XM_005042040.1	TUBB6
XM_005042005.2	SH3PXD2A
XM_005048677.1	IDE
XM_005048419.2	DNAJB12
XM_005047986.2	**GSTK1**
XM_005042269.2	**WWP1**
XM_016301762.1	**NAGPA**
XM_005044724.2	**PPM1K**
XM_005038401.1	DNAL1
XM_005038091.1	**ZDHHC20**
XM_005062085.1	**ASIC1**
XM_005046991.1	**MYOC**
XM_005049866.1	PLPP6
XM_005050007.1	NHP2
XM_005053617.2	LOC101815973
XM_005042611.1	OSTN
XM_005051099.1	MECOM
XM_005051315.2	CDADC1
XM_016296125.1	**SUGT1**
XM_005037953.1	RPS25
XM_016303810.1	TCIRG1
XM_016299107.1	ACSS3
XM_005039906.1	MYF5
XM_016304331.1	ARAP2
XM_005045389.2	SPECC1L
XM_005045346.2	MAPK1
XM_005054795.1	LOC101809314
XM_005054910.2	IMPG1
XM_005043131.2	TSR3
XM_005049273.2	**Osgepl1**
XM_016297079.1	LOC101822112
XM_005054684.2	SLC22A2
XM_005043198.2	RAB26

XM_005043614.2	SH3BGR
XM_005054428.1	**SDK1**
XM_005054008.1	**RUNX1**
XM_005037142.2	RIPK4
XM_016298043.1	LOC101812359
XM_005037183.2	NACA
XM_005045614.1	CTSD
XM_005061387.2	ERH
XM_005046816.1	LOC101810161
XM_005047384.1	PAPSS2
XM_005040064.1	ATP10B
XM_005048086.1	SPATA5L1
XM_016301575.1	AKAP13
XM_016300850.1	**CBLL1**
XM_005051920.2	**FGF7**
XM_016300780.1	RELN
XM_005039138.2	GNG10
XM_016303057.1	**GNB1**
XM_016304620.1	LOC101817904
XM_005057597.2	TRH
XM_005038109.2	LOC101809883
XM_005048122.2	**BMPR1A_1**
XM_005053334.1	ATIC
XM_005037520.1	KCND3
XM_005048986.2	LOC101810386
XM_005059186.1	LOC107603674
XM_005059571.1	LOC101813292
XM_016298945.1	TAF4
XM_005037873.2	**HTR2A**
XM_002194111.2	PRUNE1
XM_016298921.1	PKP3
XM_005057359.1	ALDH3A2
XM_016300832.1	**TRPM7**
XM_005046501.2	BAIAP2L2
XM_002190982.3	**SENP1**
XM_005056669.2	WDR12
XM_005046073.2	**GJB1**
XM_005039746.2	RFC1
XM_005049157.1	MTBP
XM_016298059.1	SPCS3
XM_016296127.1	RPRML
XM_005045169.1	RASGEF1A
XM_005059547.1	NTRK2
XM_005048151.2	APOBEC2
XM_016305207.1	ZCCHC7
XM_005059489.1	CENPK
XM_016304871.1	DNAH9
XM_016304884.1	DCST2
XM_010405265.2	Cox7a2
XM_016302782.1	DNAH9
XM_005059010.2	DCST2
XM_002192079.3	**PI4KB**

Supplementary Table S8: Summary of gene gain and loss events inferred after correcting for annotation and assembly error across all 13 species. The number of rapidly evolving families is shown in parentheses for each type of change.

	Expansions			Contractions			No Change	Avg. Expansion
	Families	Genes gained	Genes/ expansion	Families	Genes lost	Genes/ contraction		
**Paradisaea**	248 (40)	297	1.2	209 (3)	215	1.03	8555	0.009323
**Astrapia**	314 (40)	398	1.27	455 (31)	543	1.19	8243	-0.016537
**Ficedula**	329 (23)	480	1.46	560 (7)	671	1.2	8123	-0.020977
**Lycocorax**	513 (16)	612	1.19	338 (2)	358	1.06	8161	0.027747
**Taeniopygia**	1463 (17)	2009	1.37	977 (7)	1040	1.06	6572	0.091565
**Ptiloris**	334 (49)	401	1.2	203 (5)	219	1.08	8475	0.020200
**Pteridophora**	241 (13)	274	1.14	297 (6)	309	1.04	8474	-0.002997
**Corvus**	362 (6)	480	1.33	1708 (45)	2050	1.2	6942	-0.172475

Supplementary Table S9: Assembly/Annotation error estimation and gene gain/loss rates in a single λ model in the 13 mammals included in this study compared to previous studies using fewer species.

	λ (No Error Model)	ε (Estimated error)	λ (Error Model = ε)
**8 bird species in this study**	0.00221	0.01025	0.00205
**12 Drosophila species***	0.00121	0.04102	0.00059
**10 mammal species***	0.00238	0.07324	0.00186
**16 fungi species***	0.0008	0.02771	0.00061

* Dataset from Han et al. 2013 [1].

Supplementary Table S10: **Enriched GO terms in rapidly evolving birds-of-paradise families.** The number in parentheses for rapidly evolving lineages indicates the extent of change along that lineage (e.g. *Astrapia* (+6) means that the *Astrapia* lineage gained 6 genes). Lineages within the BOP clade are indicated by bold text. See Figure S1 for internal node labels.

Family ID	GO accession: GO name	Rapidly evolving lineages	Enriched after FDR correction?
1	GO:0001077: transcriptional activator activity, RNA polymerase II core promoter proximal region sequence-specific binding	**Astrapia (+6)**<br>BOP13 (-17)<br>BOP1 (+9)<br>Taeniopygia (+36)<br>**Pteridophora (-4)**<br>Crow (-12)	*
1	GO:0001078: transcriptional repressor activity, RNA polymerase II core promoter proximal region sequence-specific binding	**Astrapia (+6)**<br>BOP13 (-17)<br>BOP1 (+9)<br>Taeniopygia (+36)<br>**Pteridophora (-4)**<br>Crow (-12)	
1	GO:0000978: RNA polymerase II core promoter proximal region sequence-specific DNA binding	**Astrapia (+6)**<br>BOP13 (-17)<br>BOP1 (+9)<br>Taeniopygia (+36)<br>**Pteridophora (-4)**<br>Crow (-12)	*
1	GO:0000977: RNA polymerase II regulatory region sequence-specific DNA binding	**Astrapia (+6)**<br>BOP13 (-17)<br>BOP1 (+9)<br>Taeniopygia (+36)<br>**Pteridophora (-4)**<br>Crow (-12)	*
1	GO:0001223: transcription coactivator binding	**Astrapia (+6)**<br>BOP13 (-17)<br>BOP1 (+9)<br>Taeniopygia (+36)<br>**Pteridophora (-4)**<br>Crow (-12)	
13	GO:0004984: olfactory receptor activity	**Astrapia (+6)**<br>**Lycocorax (-6)**<br>Taeniopygia (+17)<br>**BOP9 (+5)**<br>Crow (-9)	*
26	GO:0005200: structural constituent of cytoskeleton	**BOP11 (-26)**<br>Ficedula (+16)<br>BOP13 (-21)<br>BOP1 (+12)<br>Taeniopygia (+20)<br>**BOP9 (-3)**	*

30	GO:0001948: glycoprotein binding	**Paradisaea (+6)**<br>**Astrapia (-3)**	
30	GO:0005041: low-density lipoprotein receptor activity	**Paradisaea (+6)**<br>**Astrapia (-3)**	
31	GO:0001964: startle response	**Paradisaea (+3)**<br>**BOP9 (+5)**	
31	GO:0008344: adult locomotory behavior	**Paradisaea (+3)**<br>**BOP9 (+5)**	*
36	GO:0005112: Notch binding	**Ptiloris (+3)**	*
39	GO:0005198: structural molecule activity	**BOP9 (-3)**	*
49	GO:0003777: microtubule motor activity	**Astrapia (+3)**<br>Crow (+15)	*
65	GO:0004222: metalloendopeptidase activity	**Astrapia (+4)**<br>Ptiloris (-3)	*
67	GO:0005216: ion channel activity	**BOP11 (+4)**<br>Crow (-4)	*
76	GO:0001657: ureteric bud development	**BOP11 (+3)**<br>Crow (-4)	*
97	GO:0003958: NADPH-hemoprotein reductase activity	**Astrapia (+3)**	*
97	GO:0004517: nitric-oxide synthase activity	**Astrapia (+3)**	*
97	GO:0005272: sodium channel activity	**Astrapia (+3)**	*
102	GO:0007155: cell adhesion	**Ptiloris (-2)**<br>Crow (+6)	*
121	GO:0060348: bone development	**Astrapia (-4)**	
121	GO:0002091: negative regulation of receptor internalization	**Astrapia (-4)**	*
130	GO:0005201: extracellular matrix structural constituent	**Paradisaea (+3)**	
138	GO:0014719: skeletal muscle satellite cell activation	**BOP5 (+1)**	*
170	GO:0006955: immune response	**Astrapia (-2)**<br>Taeniopygia (+8)<br>**Ptiloris (+2)**<br>**BOP5 (-3)**<br>Crow (-4)	*

252	GO:0019992: diacylglycerol binding	**BOP11 (+2)**<br>**Pteridophora (+2)**	*
290	GO:0030234: enzyme regulator activity	**Paradisaea (-3)**	
312	GO:0003956: NAD(P)+-protein-arginine ADP-ribosyltransferase activity	**Paradisaea (+2)**<br>Ficedula (+5)<br>Taeniopygia (-3)<br>**Ptiloris (+2)**<br>**BOP7 (+1)**<br>Crow (-3)	
321	GO:0017112: Rab guanyl-nucleotide exchange factor activity	**Ptiloris (-2)**<br>**Pteridophora (+2)**	*
477	GO:0050699: WW domain binding	**BOP11 (+2)**	
584	GO:0004415: hyalurononglucosaminidase activity	**Lycocorax (+4)**	*
637	GO:0005149: interleukin-1 receptor binding	**Astrapia (+2)**<br>**BOP9 (-2)**	*
836	GO:0017080: sodium channel regulator activity	**Paradisaea (+3)**<br>**BOP9 (-2)**	
867	GO:0002060: purine nucleobase binding	**Paradisaea (+2)**<br>**Ptiloris (+2)**	*
867	GO:0004645: phosphorylase activity	**Paradisaea (+2)**<br>**Ptiloris (+2)**	*

References

1. Han, M.V., Thomas, G.W., Lugo-Martinez, J., and Hahn, M.W. (2013). Estimating gene gain and loss rates in the presence of error in genome assembly and annotation using CAFE 3. Molecular Biology and Evolution *30*, 1987-1997.
