# Supplementary material for: Comparative analyses identify genomic features potentially involved in the evolution of birds-of-paradise
Source: Gigascience. 2019 Jan 24;8(5):giz003. doi: 10.1093/gigascience/giz003 (PMC6497032; doi:10.1093/gigascience/giz003)
Supplement: Supplemental File [file giz003_supplemental_file.docx]

**Supplementary**

**Supplementary Figures**


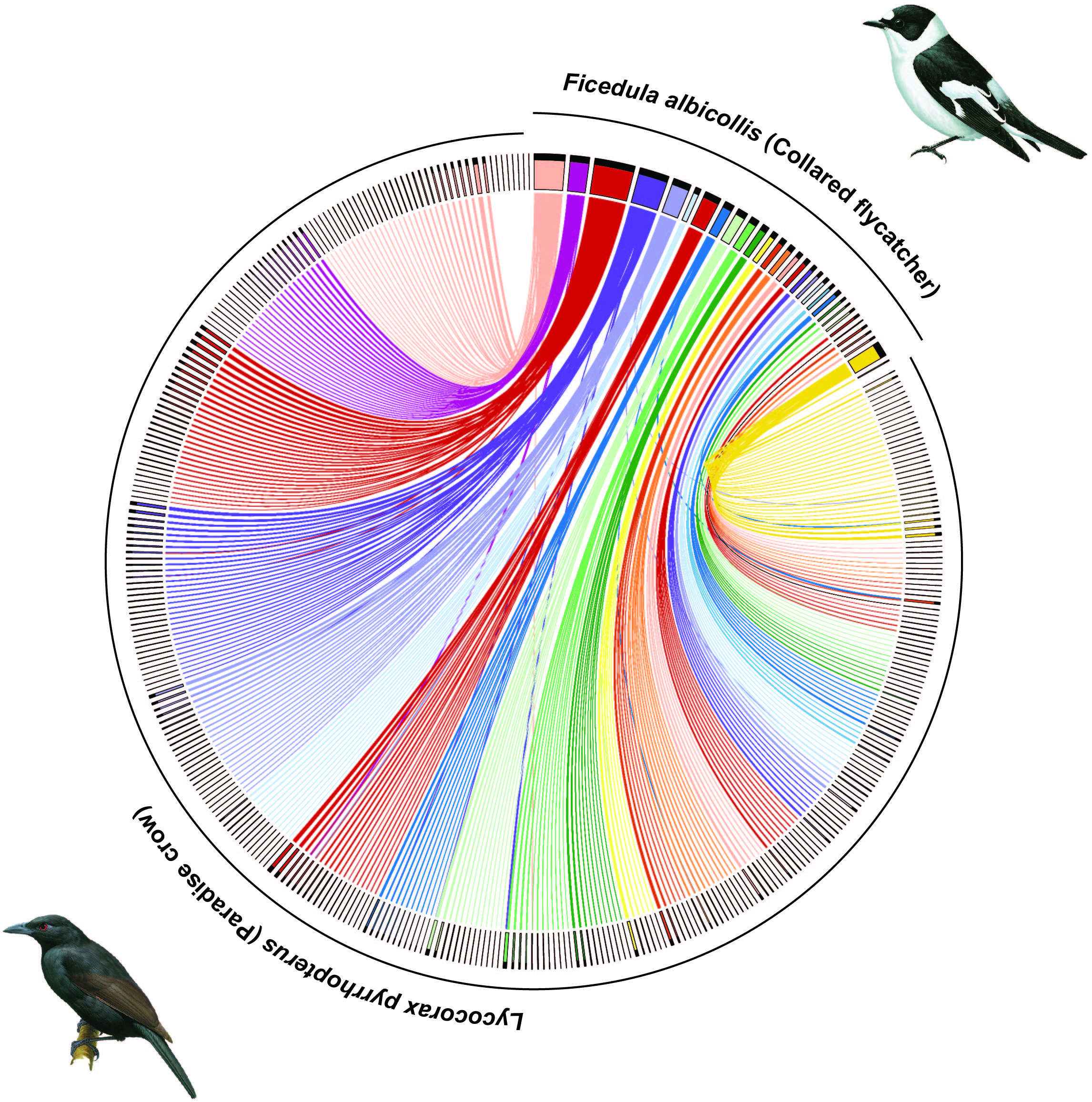


**Supplementary Figure S1: Chromosomal synteny plot between the collared flycatcher and the paradise crow.** The plot shows scaffolds larger than 50 kb and links (alignments) larger than 2 kb.


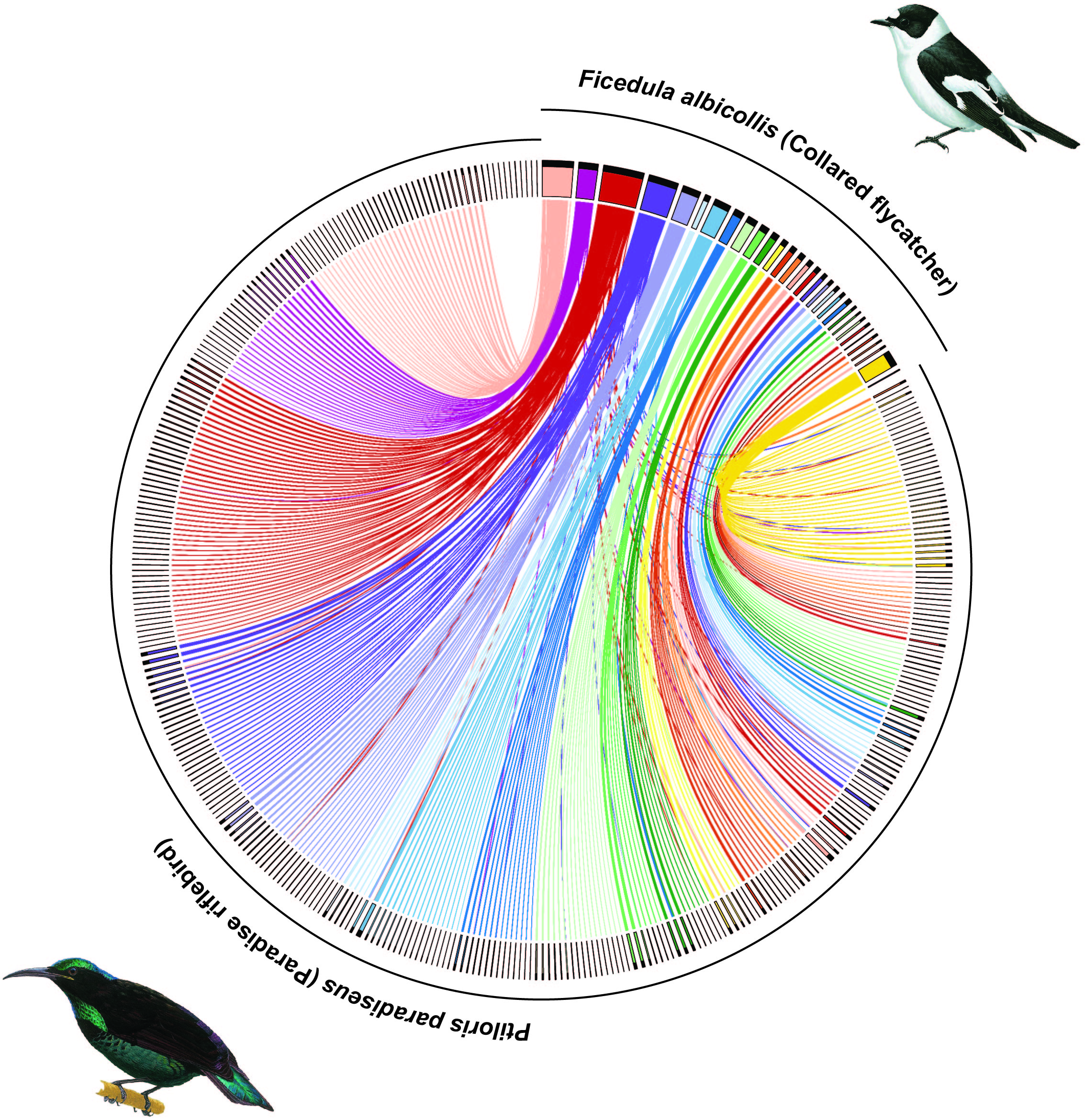


**Supplementary Figure S2: Chromosomal synteny plot between the collared flycatcher and the paradise riflebird.** The plot shows scaffolds larger than 50 kb and links (alignments) larger than 2 kb.


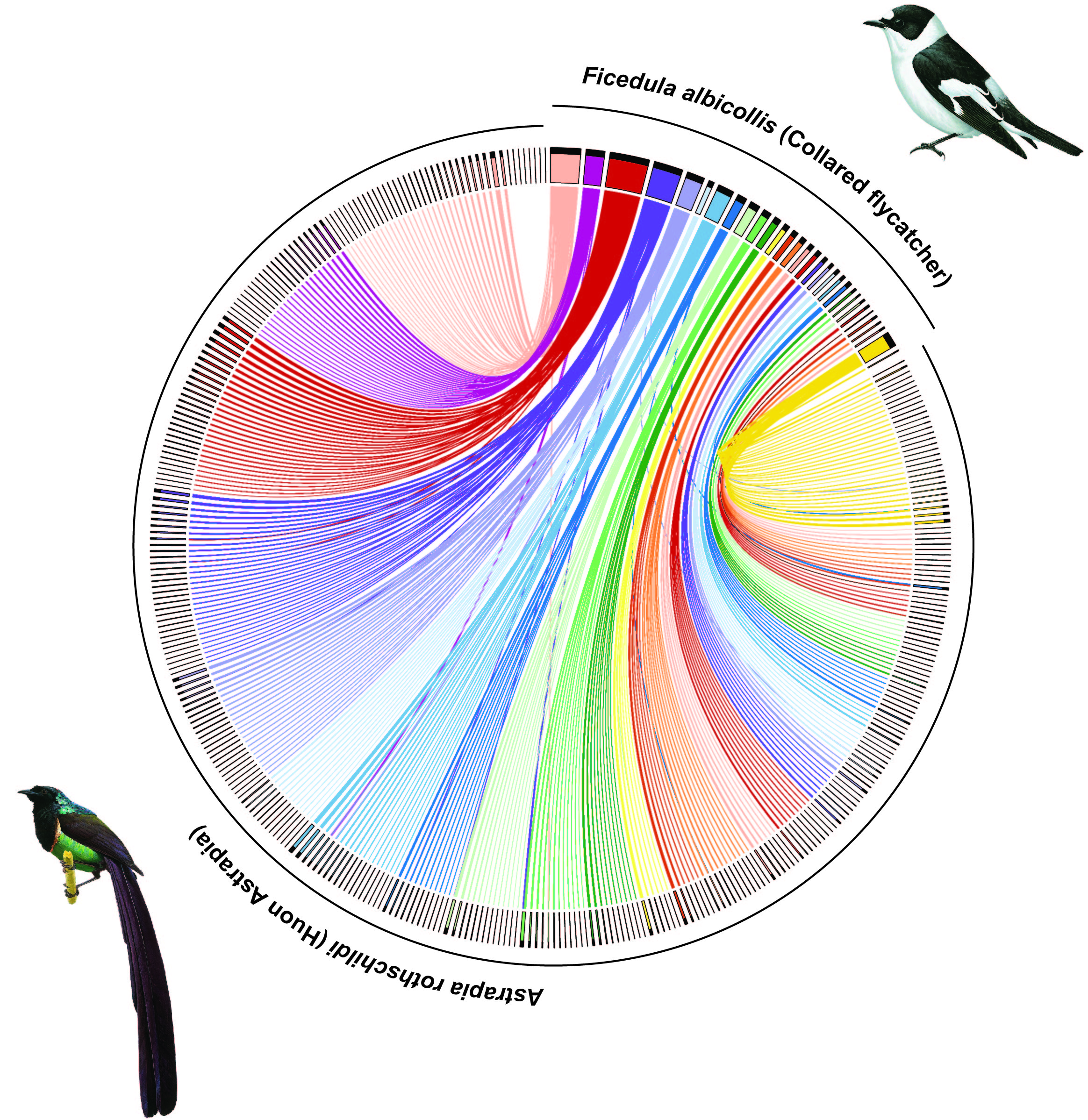


**Supplementary Figure S3: Chromosomal synteny plot between the collared flycatcher and the Huon Astrapia.** The plot shows scaffolds larger than 50 kb and links (alignments) larger than 2 kb.

**Supplementary Figure S4: Phylogenetic species tree.** The species tree was reconstructed from individual maximum likelihood-based gene trees using 4,656 exons and coalescent-based statistical binning (Astral). Branch lengths are depicted on the branches (calculated via a ML tree constructed using ExaML and 200 randomly selected genes). Nodes are labeled and concordance factor is shown next to the node labels (i.e. [node label] / [concordance factor]). All nodes have 100 bootstrap support.

**Supplementary Tables**

**Supplementary Table S1: BUSCO scores.** Scores were calculated using Busco2 and the aves_odb9 data set (4,915 genes total).

|  | **Complete** | **Duplicated** | **Fragmented** | **Missing** |
| --- | --- | --- | --- | --- |
| *Astrapia rothschildi* | 4,669 (95.0%) | 48 (1.0%) | 139 (2.8%) | 107 (2.2%) |
| *Lycocorax pyrrhopterus* | 4,659 (93.8%) | 51 (1.0%) | 161 (3.3%) | 95 (1.9%) |
| *Ptiloris paradiseus* | 4,675 (95.1%) | 44 (0.9%) | 135 (2.7%) | 105 (2.2%) |
| *Paradisaea rubra* | 4,662 (94.9%) | 47 (1.0%) | 150 (3.1%) | 103 (2.0%) |
| *Pteridophora alberti* | 4,661 (94.8%) | 39 (0.8%) | 155 (3.2%) | 99 (2.0%) |

**Supplementary Table S2: Gene annotation.**

|  | **# of Transcripts** | **Average transcript size (bp)** | **Average introns size (kb, rounded)** | **Average # of introns per gene** |
| --- | --- | --- | --- | --- |
| *Astrapia rothschildi* | 16,260 | 1,603 | 2.2 | 9 |
| *Lycocorax pyrrhopterus* | 17,023 | 1,572 | 2.2 | 9 |
| *Ptiloris paradiseus* | 17,269 | 1,584 | 2.2 | 9 |
| *Paradisaea rubra* | 16,822 | 1,561 | 2.2 | 9 |
| *Pteridophora alberti* | 16,721 | 1,562 | 2.2 | 9 |

**Supplementary Table S3: RepeatMasker annotation of the three birds-of-paradise genome assemblies using a library of our *de novo* repeat annotations of birds-of-paradise merged with existing avian repeat libraries.**

|  | ***Astrapia rothschildi*** | | | ***Lycocorax pyrrhopterus*** | | | ***Ptiloris paradisaeus*** | | |
| --- | --- | --- | --- | --- | --- | --- | --- | --- | --- |
| **Repeat type** | **Copies** | **Total bp** | **Total %** | **Copies** | **Total bp** | **Total %** | **Copies** | **Total bp** | **Total %** |
| **SINE** | 8,019 | 966,992 | 0.09 | 7,974 | 955,190 | 0.09 | 7,977 | 961,945 | 0.09 |
| **LINE** | 128,473 | 38,885,201 | 3.67 | 130,706 | 40,271,136 | 3.76 | 129,094 | 38,994,767 | 3.68 |
| **LTR** | 38,693 | 20,692,445 | 1.95 | 48,395 | 27,819,221 | 2.60 | 39,123 | 21,311,765 | 2.01 |
| **DNA** | 4,582 | 790,017 | 0.07 | 4,734 | 845,120 | 0.08 | 4,617 | 790,421 | 0.07 |
| **Unclassified** | 34,049 | 9,005,494 | 0.85 | 37,519 | 9,167,482 | 0.86 | 30,814 | 8,931,323 | 0.84 |
| **Total interspersed repeats** | 213,816 | 70,340,149 | 6.63 | 229,328 | 79,058,149 | 7.39 | 211,625 | 70,990,221 | 6.69 |
| **Small RNA** | 538 | 46,523 | 0.00 | 577 | 50,738 | 0.00 | 546 | 47,744 | 0.00 |
| **Satellites** | 2,884 | 623,756 | 0.06 | 2,706 | 572,161 | 0.05 | 2,855 | 646,354 | 0.06 |
| **Simple repeats** | 195,600 | 9,348,199 | 0.88 | 193,765 | 9,101,884 | 0.85 | 197,648 | 9,318,098 | 0.88 |
| **Low complexity** | 43,076 | 2,388,544 | 0.23 | 42,067 | 2,292,784 | 0.21 | 42,546 | 2,360,570 | 0.22 |
| **Total tandem repeats** | 242,098 | 12,407,022 | 1.17 | 239,115 | 12,017,567 | 1.11 | 243,595 | 12,372,766 | 1.16 |
| **Total repeats** | 455,914 | 82,747,171 | 7.80 | 468,443 | 91,075,716 | 8.50 | 455,220 | 83,362,987 | 7.85 |
| **Assembly** |  | 1.06Gb |  |  | 1.07Gb |  |  | 1.06Gb |  |
| **Gap ('N') bp** |  | 13,196,877 |  |  | 10,466,138 |  |  | 10,993,394 |  |

**Supplementary Table S4: Characteristics of the manually curated TE consensus sequences from *Lycocorax pyrrhopterus*, including lineage-specific LTR families termed as ‘lycPyrLTR*’.**

| **Class** | **Sub-**  **class** | **Superfamily** | **Family** | **Subfamily** | **Similarity to known repeats** | **Consensus status** | **Consensus length** | **TSD** |
| --- | --- | --- | --- | --- | --- | --- | --- | --- |
| Retrotransposon | LTR | ERV1 | lycPyrLTR1 | lycPyrLTR1 | None | Complete | 463 | 4 bp |
| Retrotransposon | LTR | ERV1 | lycPyrLTR2 | lycPyrLTR2 | None | Complete | 535 | 4 bp |
| Retrotransposon | LTR | ERV1 | lycPyrLTR3 | lycPyrLTR3 | None | Complete | 623 | 4 bp |
| Retrotransposon | LTR | ERV1 | TguERV3 | TguERV3_LTR2b-L_lycPyr | Partially TguERV3_LTR2b (68% similarity) | Complete | 601 | 4 bp |
| Retrotransposon | LTR | ERV1 | TguERV1 | TguERV1_LTR1a-L_lycPyr | Partially TguERV1_LTR1a (72% similarity) | Complete | 600 | 4 bp |
| Retrotransposon | LTR | ERV1 | TguLTR11 | TguLTR11l-L_lycPyr.inc | Partially TguLTR11l + TguERV2_I + TguERV1_I + TguERV3_I (79% + 65% + 63% + 66% similarity) | Incomplete 3' end | 4535 | ? |
| Retrotransposon | LTR | ERV1 | TguLTR12 | TguLTR12-L_lycPyr.inc | Partially TguLTR12 (84% similarity) | Incomplete TSD | 625 | ? |
| Retrotransposon | LTR | ERV2 | lycPyrLTRK1 | lycPyrLTRK1a | None | Complete | 366 | 6 bp |
| Retrotransposon | LTR | ERV2 | lycPyrLTRK1 | lycPyrLTRK1b | None | Complete | 366 | 6 bp |
| Retrotransposon | LTR | ERV2 | lycPyrLTRK2 | lycPyrLTRK2 | None | Complete | 647 | 6 bp |
| Retrotransposon | LTR | ERV2 | lycPyrLTRK3 | lycPyrLTRK3a | None | Complete | 689 | 6 bp |
| Retrotransposon | LTR | ERV2 | lycPyrLTRK3 | lycPyrLTRK3b | None | Complete | 744 | 6 bp |
| Retrotransposon | LTR | ERV2 | lycPyrLTRK4 | lycPyrLTRK4 | None | Complete | 605 | 6 bp |
| Retrotransposon | LTR | ERV2 | lycPyrLTRK5 | lycPyrLTRK5 | None | Complete | 397 | 6 bp |
| Retrotransposon | LTR | ERV2 | lycPyrLTRK6 | lycPyrLTRK6 | None | Complete | 666 | 6 bp |
| Retrotransposon | LTR | ERV2 | lycPyrLTRK7 | lycPyrLTRK7 | None | Complete | 334 | 6 bp |
| Retrotransposon | LTR | ERV2 | lycPyrLTRK8 | lycPyrLTRK8 | None | Complete | 408 | 6 bp |
| Retrotransposon | LTR | ERV2 | lycPyrLTRK9 | lycPyrLTRK9_LTR | None | Complete | 380 | 6 bp |
| Retrotransposon | LTR | ERV2 | lycPyrLTRK9 | lycPyrLTRK9_I.inc | None | Incomplete 3' end | 537 | 6 bp |
| Retrotransposon | LTR | ERV3 | lycPyrLTRL1 | lycPyrLTRL1 | None | Complete | 1171 | 5 bp |
| Retrotransposon | LTR | ERV3 | lycPyrLTRL2 | lycPyrLTRL2 | None | Complete | 1105 | 5 bp |
| Retrotransposon | LTR | ERV3 | lycPyrLTRL3 | lycPyrLTRL3 | None | Complete | 460 | 5 bp |
| Retrotransposon | LTR | ERV3 | lycPyrLTRL4 | lycPyrLTRL4 | None | Complete | 807 | 5 bp |
| Retrotransposon | LTR | ERV3 | lycPyrLTRL5 | lycPyrLTRL5 | None | Complete | 1281 | 5 bp |
| Retrotransposon | LTR | ERV3 | lycPyrLTRL6 | lycPyrLTRL6 | None | Complete | 670 | 5 bp |
| Retrotransposon | LTR | ERV3 | lycPyrLTRL7 | lycPyrLTRL7.inc | Partially Tgu_rep3 (80% similarity) | Incomplete 3' end | 177 | ? |
| Retrotransposon | LTR | ERV3 | TguERVL2 | TguERVL2b-LTR-L_lycPyr | Partially TguERVL2b3_LTR + TguERVL2b1_LTR (85% + 79% similarity) | Complete | 579 | 5 bp |
| Retrotransposon | LTR | ERV3 | TguERVL2 | TguERVL2a2-LTR-L_lycPyr | Partially TguERVL2a2-LTR (93% similarity) | Complete | 941 | 5 bp |
| Retrotransposon | LTR | ERV3 | TguLTRL1 | TguLTRL1-La_lycPyr | Partially TguLTRL1a7 (75% similarity) | Complete | 647 | 5 bp |
| Retrotransposon | LTR | ERV3 | TguLTRL1 | TguLTRL1-Lb_lycPyr.inc | Partially TguERVL1_I + TguLTRL1a6 + TguLTRL1a7 (88% + 77% + 74% similarity) | Incomplete 5' end | 2729 | ? |
| Retrotransposon | LTR | ERV3 | TguLTRL1 | TguLTRL1-Lc_lycPyr.inc | Partially TguERVL1_I + TguERVL2_I + TguLTRL6b (80% + 78% + 97% similarity) | Incomplete 5' and 3' ends | 1754 | ? |
| Retrotransposon | LTR | ERV3 | TguLTRL1 | TguLTRL1-Ld_lycPyr.inc | Partially TguLTRL1a6 + TguLTRL1a7 + TguLTRL1_I (77% + 75% + 75% similarity) | Incomplete 3' end | 2655 | ? |
| Retrotransposon | LTR | ERV3 | TguLTRL1 | TguLTRL1-Le_lycPyr.inc | Partially TguLTRL1a7 + TguERVL1_I (75% + 77% similarity) | Incomplete 5' and 3' ends | 3079 | ? |
| Unknown | Unknown | Unknown | Unknown | lycPyr5-275.3inc | None | Incomplete 3' end | 177 | ? |
| Unknown | Unknown | Unknown | Unknown | lycPyr5-1942.inc | None | Incomplete 5' and 3' ends | 620 | ? |
| Unknown | Unknown | Unknown | Unknown | lycPyr6-947.inc_sat | None | Incomplete 5' and 3' ends | 3602 | ? |

**Supplementary Table S5: Top 10 gene tree topology counts (423 total topologies in 4,450 rooted gene trees).** Average Robinson-Foulds distance for all 4,656 gene trees is 3.92. Z: zebra finch; F: collared flycatcher; C: hooded crow; L: *Lycocorax*; Pte: *Pteridophora*; Pti: *Ptiloris*; Par: *Paradisaea*; A: *Astrapia*.

| **Topology** | **Count** |
| --- | --- |
| ((Z,F),(C,(L,(Pte,(Pti,(Par,A)))))) | 430 |
| ((Z,F),(C,(L,(Pte,((Pti,Par),A))))) | 357 |
| ((Z,F),(C,(L,(Pte,(Par,(Pti,A)))))) | 279 |
| ((Z,F),(C,(L,(Pti,(Pte,(Par,A)))))) | 224 |
| ((Z,F),(C,(L,((Pti,Par),(Pte,A))))) | 167 |
| ((Z,F),(C,(L,(Pti,((Pte,Par),A))))) | 166 |
| ((Z,F),(C,(L,(((Pti,Par),Pte),A)))) | 162 |
| ((Z,F),(C,(L,((Pte,Pti),(Par,A))))) | 161 |
| ((Z,F),(C,(L,((Pti,(Pte,Par)),A)))) | 159 |
| ((Z,F),(C,(L,(Pti,(Par,(Pte,A)))))) | 156 |

**Supplementary Table S6: Saturation Analysis. Pairwise dN/dS ratio.**

| **Astrapia** |  |  |  |  |  |  |  |
| --- | --- | --- | --- | --- | --- | --- | --- |
| **Corvus** | 0.036 |  |  |  |  |  |  |
| **Ficedula** | 0.046 | 0.063 |  |  |  |  |  |
| **Taeniopygia** | 0.044 | 0.059 | 0.046 |  |  |  |  |
| **Lycocorax** | 0.014 | 0.037 | 0.046 | 0.044 |  |  |  |
| **Paradisaea** | 0.006 | 0.034 | 0.046 | 0.043 | 0.014 |  |  |
| **Pteridophora** | 0.007 | 0.036 | 0.046 | 0.044 | 0.014 | 0.007 |  |
| **Ptiloris** | 0.006 | 0.036 | 0.046 | 0.044 | 0.014 | 0.006 | 0.007 |

**Supplementary Table S7. Genes under positive selection.** Gene symbols in bold mark genes significant after multiple-testing correction using FDR (<0.05 cut-off).

| **GenBank Accession** | **Gene Symbol** |
| --- | --- |
| XM_016302299.1 | **RSPH14** |
| XM_005060676.1 | SNX18 |
| XM_005061576.2 | RSG1 |
| XM_016299097.1 | COL4A1 |
| XM_016304880.1 | MCTP1 |
| XM_016303427.1 | **FGD6** |
| XM_005042552.1 | NDRG1 |
| XM_005062657.1 | MRPL34 |
| XM_005057187.1 | BPIFB2 |
| XM_010403165.3 | C8orf48 |
| XM_016298377.1 | LOC101809528 |
| XM_005057074.2 | LOC101821424 |
| XM_010401845.3 | **camC** |
| XM_016301269.1 | LOC101807976 |
| XM_016302077.1 | TRAFD1 |
| XM_005048141.1 | SH2D4B |
| XM_005044853.1 | MGARP |
| XM_005039384.1 | ADAMTS20 |
| XM_005037072.1 | TAF10 |
| XM_005054309.2 | C14H16orf71 |
| XM_005056583.1 | EVI2A |
| - | guaA |
| XM_005038551.2 | CCDC181 |
| XM_016298245.1 | COL4A5 |
| XM_005059334.2 | LAD1 |
| XM_016301828.1 | LOC101813372 |
| XM_019280702.1 | **act-2b** |
| XM_016296212.1 | AGAP3 |
| XM_005051230.1 | FETUB |
| XM_005043410.1 | POLH |
| XM_004175487.1 | **CORIN** |
| XM_019282258.2 | **Slc30a10** |
| XM_005054364.2 | **DRC3** |
| - | **PRP5** |
| XM_016300815.1 | ZWILCH |
| XM_005043438.1 | WDR27 |
| XM_005058894.2 | S100A11 |
| XM_005046922.2 | C5H11orf74 |
| XM_016306090.1 | SLC9A2 |
| XM_005050076.2 | LOC101808676 |
| XM_016300582.1 | ATP7B |
| XM_005051988.2 | **TCF12** |
| XM_016303963.1 | LOC107604184 |
| XM_005061593.1 | LOC101821569 |
| XM_016298934.1 | ITPK1 |
| XM_016302041.1 | CARHSP1 |
| XM_005049042.2 | LOC101807582 |
| XM_016303572.1 | IDO2 |
| XM_005052727.1 | LOC101813437 |
| XM_005047366.2 | GPATCH2L |
| XM_005057073.2 | WISP2 |
| XM_016303591.1 | **ADD2** |
| XM_005055863.2 | PMP22 |
| XM_005062304.2 | IGSF21 |
| XM_016305344.1 | **LOC101812147** |
| XM_005050061.1 | CABP4 |
| XM_016298968.1 | LOC101816855 |
| XM_005056673.2 | LOC101817428 |
| XM_005056783.1 | CBX2 |
| XM_005056335.1 | PTDSS1 |
| XM_005042325.2 | BARHL1 |
| XM_005055795.1 | HABP2 |
| XM_005048773.2 | SYTL1 |
| XM_016303687.1 | PAQR7 |
| XM_016303706.1 | CCDC89 |
| XM_016301660.1 | KIF3C |
| XM_016305278.1 | PHLDA3 |
| XM_005059500.2 | PHLDA1 |
| XM_016303529.1 | **LOC101807907** |
| XM_016299105.1 | GPX2 |
| XM_005044014.1 | **TPBG** |
| XM_005049721.1 | LOC101813208 |
| XM_005039251.1 | RNASEL |
| XM_005049817.2 | GPR88 |
| XM_005050542.1 | NEXMIF |
| XM_005045976.1 | LOC101813871 |
| XM_005061804.2 | MOB3C |
| XM_005050079.2 | ITPKB |
| XM_005043012.2 | LOC101811548 |
| XM_005061100.2 | SLC12A3 |
| XM_005052526.1 | LOC101822159 |
| XM_005060823.1 | DOCK8 |
| XM_005060810.2 | SLC7A2 |
| XM_016298010.1 | G6PC2 |
| XM_016299897.1 | ZEB1 |
| XM_005040638.2 | CCDC149 |
| XM_005045593.1 | ALAD |
| XM_005055395.2 | SPAG16 |
| XM_005048989.2 | HAUS1 |
| XM_016299631.1 | **NCKAP1** |
| XM_005060340.1 | SLC25A27 |
| XM_005037255.2 | **Atp6ap2** |
| XM_005044316.2 | PTPN11 |
| XM_005055265.2 | SLC25A10 |
| XM_005056255.2 | LOC101816285 |
| XM_016298524.1 | TSPAN9 |
| XM_005061480.2 | TCAF2 |
| XM_005061498.2 | ANXA11 |
| XM_016299390.1 | TAF11 |
| XM_005059540.1 | NCLN |
| XM_016304488.1 | NR2E3 |
| XM_010400678.3 | **MAML2** |
| XM_005051635.1 | P3H4 |
| XM_005059699.2 | GAB2 |
| XM_005044075.1 | **Lyrm2** |
| XM_005038313.1 | AGL |
| XM_016300131.1 | TERT |
| XM_005058028.2 | **PPP2R2A** |
| XM_016296086.1 | FGFR1 |
| XM_016303624.1 | GCN1 |
| XM_005040177.1 | **PARVB** |
| XM_005055228.2 | LIMS1 |
| XM_005037507.2 | PAFAH1B2 |
| XM_005058597.2 | LOC101821368 |
| XM_005060625.1 | UBE2Q1 |
| XM_005059102.1 | MZT1 |
| XM_016297193.1 | B3GLCT |
| XM_005038138.1 | PDS5B |
| XM_005038022.1 | LMO4 |
| XM_005049530.2 | **asic4** |
| XM_016300038.1 | RBP2 |
| XM_005050724.1 | CYFIP2 |
| XM_010399761.3 | **Pdgfrb** |
| XM_005053530.2 | STRA8 |
| XM_005039572.1 | TAF4B |
| XM_016300054.1 | **SERBP1** |
| XM_005041939.2 | **COLEC12** |
| XM_005042040.1 | TUBB6 |
| XM_005042005.2 | SH3PXD2A |
| XM_005048677.1 | IDE |
| XM_005048419.2 | DNAJB12 |
| XM_005047986.2 | **GSTK1** |
| XM_005042269.2 | **WWP1** |
| XM_016301762.1 | **NAGPA** |
| XM_005044724.2 | **PPM1K** |
| XM_005038401.1 | DNAL1 |
| XM_005038091.1 | **ZDHHC20** |
| XM_005062085.1 | **ASIC1** |
| XM_005046991.1 | **MYOC** |
| XM_005049866.1 | PLPP6 |
| XM_005050007.1 | NHP2 |
| XM_005053617.2 | LOC101815973 |
| XM_005042611.1 | OSTN |
| XM_005051099.1 | MECOM |
| XM_005051315.2 | CDADC1 |
| XM_016296125.1 | **SUGT1** |
| XM_005037953.1 | RPS25 |
| XM_016303810.1 | TCIRG1 |
| XM_016299107.1 | ACSS3 |
| XM_005039906.1 | MYF5 |
| XM_016304331.1 | ARAP2 |
| XM_005045389.2 | SPECC1L |
| XM_005045346.2 | MAPK1 |
| XM_005054795.1 | LOC101809314 |
| XM_005054910.2 | IMPG1 |
| XM_005043131.2 | TSR3 |
| XM_005049273.2 | **Osgepl1** |
| XM_016297079.1 | LOC101822112 |
| XM_005054684.2 | SLC22A2 |
| XM_005043198.2 | RAB26 |
| XM_005043614.2 | SH3BGR |
| XM_005054428.1 | **SDK1** |
| XM_005054008.1 | **RUNX1** |
| XM_005037142.2 | RIPK4 |
| XM_016298043.1 | LOC101812359 |
| XM_005037183.2 | NACA |
| XM_005045614.1 | CTSD |
| XM_005061387.2 | ERH |
| XM_005046816.1 | LOC101810161 |
| XM_005047384.1 | PAPSS2 |
| XM_005040064.1 | ATP10B |
| XM_005048086.1 | SPATA5L1 |
| XM_016301575.1 | AKAP13 |
| XM_016300850.1 | **CBLL1** |
| XM_005051920.2 | **FGF7** |
| XM_016300780.1 | RELN |
| XM_005039138.2 | GNG10 |
| XM_016303057.1 | **GNB1** |
| XM_016304620.1 | LOC101817904 |
| XM_005057597.2 | TRH |
| XM_005038109.2 | LOC101809883 |
| XM_005048122.2 | **BMPR1A_1** |
| XM_005053334.1 | ATIC |
| XM_005037520.1 | KCND3 |
| XM_005048986.2 | LOC101810386 |
| XM_005059186.1 | LOC107603674 |
| XM_005059571.1 | LOC101813292 |
| XM_016298945.1 | TAF4 |
| XM_005037873.2 | **HTR2A** |
| XM_002194111.2 | PRUNE1 |
| XM_016298921.1 | PKP3 |
| XM_005057359.1 | ALDH3A2 |
| XM_016300832.1 | **TRPM7** |
| XM_005046501.2 | BAIAP2L2 |
| XM_002190982.3 | **SENP1** |
| XM_005056669.2 | WDR12 |
| XM_005046073.2 | **GJB1** |
| XM_005039746.2 | RFC1 |
| XM_005049157.1 | MTBP |
| XM_016298059.1 | SPCS3 |
| XM_016296127.1 | RPRML |
| XM_005045169.1 | RASGEF1A |
| XM_005059547.1 | NTRK2 |
| XM_005048151.2 | APOBEC2 |
| XM_016305207.1 | ZCCHC7 |
| XM_005059489.1 | CENPK |
| XM_016304871.1 | DNAH9 |
| XM_016304884.1 | DCST2 |
| XM_010405265.2 | Cox7a2 |
| XM_016302782.1 | DNAH9 |
| XM_005059010.2 | DCST2 |
| XM_002192079.3 | **PI4KB** |

**Supplementary Table S8: Summary of gene gain and loss events inferred after correcting for annotation and assembly error across all 13 species.** The number of rapidly evolving families is shown in parentheses for each type of change.

|  | **Expansions** |  |  | **Contractions** |  |  | **No Change** | **Avg. Expansion** |
| --- | --- | --- | --- | --- | --- | --- | --- | --- |
|  | **Families** | **Genes gained** | **Genes/**  **expansion** | **Families** | **Genes lost** | **Genes/**  **contraction** |  |  |
| **Paradisaea** | 248 (40) | 297 | 1.2 | 209 (3) | 215 | 1.03 | 8555 | 0.009323 |
| **Astrapia** | 314 (40) | 398 | 1.27 | 455 (31) | 543 | 1.19 | 8243 | -0.016537 |
| **Ficedula** | 329 (23) | 480 | 1.46 | 560 (7) | 671 | 1.2 | 8123 | -0.020977 |
| **Lycocorax** | 513 (16) | 612 | 1.19 | 338 (2) | 358 | 1.06 | 8161 | 0.027747 |
| **Taeniopygia** | 1463 (17) | 2009 | 1.37 | 977 (7) | 1040 | 1.06 | 6572 | 0.091565 |
| **Ptiloris** | 334 (49) | 401 | 1.2 | 203 (5) | 219 | 1.08 | 8475 | 0.020200 |
| **Pteridophora** | 241 (13) | 274 | 1.14 | 297 (6) | 309 | 1.04 | 8474 | -0.002997 |
| **Corvus** | 362 (6) | 480 | 1.33 | 1708 (45) | 2050 | 1.2 | 6942 | -0.172475 |

**Supplementary Table S9: Assembly/Annotation error estimation and gene gain/loss rates in a single *λ* model in the 13 mammals included in this study compared to previous studies using fewer species**.

|  | ***λ* (No Error Model)** | ***ε* (Estimated error)** | ***λ* (Error Model = *ε*)** |
| --- | --- | --- | --- |
| **8 bird species in this study** | 0.00221 | 0.01025 | 0.00205 |
| **12 Drosophila species*** | 0.00121 | 0.04102 | 0.00059 |
| **10 mammal species*** | 0.00238 | 0.07324 | 0.00186 |
| **16 fungi species*** | 0.0008 | 0.02771 | 0.00061 |

* Dataset from Han et al. 2013 [1].

**Supplementary Table S10**: **Enriched GO terms in rapidly evolving birds-of-paradise families.** The number in parentheses for rapidly evolving lineages indicates the extent of change along that lineage (e.g. *Astrapia* (+6) means that the *Astrapia* lineage gained 6 genes). Lineages within the BOP clade are indicated by bold text. See Figure S1 for internal node labels.

| **Family ID** | **GO accession: GO name** | **Rapidly evolving lineages** | **Enriched after FDR correction?** |
| --- | --- | --- | --- |
| 1 | GO:0001077: transcriptional activator activity, RNA polymerase II core promoter proximal region sequence-specific binding | **Astrapia (+6)**  BOP13 (-17)  BOP1 (+9)  Taeniopygia (+36)  **Pteridophora (-4)**  Crow (-12) | * |
| 1 | GO:0001078: transcriptional repressor activity, RNA polymerase II core promoter proximal region sequence-specific binding | **Astrapia (+6)**  BOP13 (-17)  BOP1 (+9)  Taeniopygia (+36)  **Pteridophora (-4)**  Crow (-12) |  |
| 1 | GO:0000978: RNA polymerase II core promoter proximal region sequence-specific DNA binding | **Astrapia (+6)**  BOP13 (-17)  BOP1 (+9)  Taeniopygia (+36)  **Pteridophora (-4)**  Crow (-12) | * |
| 1 | GO:0000977: RNA polymerase II regulatory region sequence-specific DNA binding | **Astrapia (+6)**  BOP13 (-17)  BOP1 (+9)  Taeniopygia (+36)  **Pteridophora (-4)**  Crow (-12) | * |
| 1 | GO:0001223: transcription coactivator binding | **Astrapia (+6)**  BOP13 (-17)  BOP1 (+9)  Taeniopygia (+36)  **Pteridophora (-4)**  Crow (-12) |  |
| 13 | GO:0004984: olfactory receptor activity | **Astrapia (+6)**  **Lycocorax (-6)**  Taeniopygia (+17)  **BOP9 (+5)**  Crow (-9) | * |
| 26 | GO:0005200: structural constituent of cytoskeleton | **BOP11 (-26)**  Ficedula (+16)  BOP13 (-21)  BOP1 (+12)  Taeniopygia (+20)  **BOP9 (-3)** | * |
| 30 | GO:0001948: glycoprotein binding | **Paradisaea (+6)**  **Astrapia (-3)** |  |
| 30 | GO:0005041: low-density lipoprotein receptor activity | **Paradisaea (+6)**  **Astrapia (-3)** |  |
| 31 | GO:0001964: startle response | **Paradisaea (+3)**  **BOP9 (+5)** |  |
| 31 | GO:0008344: adult locomotory behavior | **Paradisaea (+3)**  **BOP9 (+5)** | * |
| 36 | GO:0005112: Notch binding | **Ptiloris (+3)** | * |
| 39 | GO:0005198: structural molecule activity | **BOP9 (-3)** | * |
| 49 | GO:0003777: microtubule motor activity | **Astrapia (+3)**  Crow (+15) | * |
| 65 | GO:0004222: metalloendopeptidase activity | **Astrapia (+4)**  Ptiloris (-3) | * |
| 67 | GO:0005216: ion channel activity | **BOP11 (+4)**  Crow (-4) | * |
| 76 | GO:0001657: ureteric bud development | **BOP11 (+3)**  Crow (-4) | * |
| 97 | GO:0003958: NADPH-hemoprotein reductase activity | **Astrapia (+3)** | * |
| 97 | GO:0004517: nitric-oxide synthase activity | **Astrapia (+3)** | * |
| 97 | GO:0005272: sodium channel activity | **Astrapia (+3)** | * |
| 102 | GO:0007155: cell adhesion | **Ptiloris (-2)**  Crow (+6) | * |
| 121 | GO:0060348: bone development | **Astrapia (-4)** |  |
| 121 | GO:0002091: negative regulation of receptor internalization | **Astrapia (-4)** | * |
| 130 | GO:0005201: extracellular matrix structural constituent | **Paradisaea (+3)** |  |
| 138 | GO:0014719: skeletal muscle satellite cell activation | **BOP5 (+1)** | * |
| 170 | GO:0006955: immune response | **Astrapia (-2)**  Taeniopygia (+8)  **Ptiloris (+2)**  **BOP5 (-3)**  Crow (-4) | * |
| 252 | GO:0019992: diacylglycerol binding | **BOP11 (+2)**  **Pteridophora (+2)** | * |
| 290 | GO:0030234: enzyme regulator activity | **Paradisaea (-3)** |  |
| 312 | GO:0003956: NAD(P)+-protein-arginine ADP-ribosyltransferase activity | **Paradisaea (+2)**  Ficedula (+5)  Taeniopygia (-3)  **Ptiloris (+2)**  **BOP7 (+1)**  Crow (-3) |  |
| 321 | GO:0017112: Rab guanyl-nucleotide exchange factor activity | **Ptiloris (-2)**  **Pteridophora (+2)** | * |
| 477 | GO:0050699: WW domain binding | **BOP11 (+2)** |  |
| 584 | GO:0004415: hyalurononglucosaminidase activity | **Lycocorax (+4)** | * |
| 637 | GO:0005149: interleukin-1 receptor binding | **Astrapia (+2)**  **BOP9 (-2)** | * |
| 836 | GO:0017080: sodium channel regulator activity | **Paradisaea (+3)**  **BOP9 (-2)** |  |
| 867 | GO:0002060: purine nucleobase binding | **Paradisaea (+2)**  **Ptiloris (+2)** | * |
| 867 | GO:0004645: phosphorylase activity | **Paradisaea (+2)**  **Ptiloris (+2)** | * |

**References**

1. Han, M.V., Thomas, G.W., Lugo-Martinez, J., and Hahn, M.W. (2013). Estimating gene gain and loss rates in the presence of error in genome assembly and annotation using CAFE 3. Molecular Biology and Evolution *30*, 1987-1997.
