# Supplementary material for: Comparative analyses identify genomic features potentially involved in the evolution of birds-of-paradise
Source: Gigascience. 2019 Jan 24;8(5):giz003. doi: 10.1093/gigascience/giz003 (PMC6497032; doi:10.1093/gigascience/giz003)
Supplement: Reviewer_1_Report_Original_Submission -- Scott Taylor — 5/31/2018 Reviewed [file giz003_reviewer_1_report_original_submission.pdf]

Reviewer Report

Title: Comparative analyses identify genomic features potentially involved in the evolution of birds-of-paradise.

Version: Original Submission **Date:** 5/31/2018

Reviewer name: Scott Taylor

Reviewer Comments to Author:

Prost et al. use whole genome sequence data from five bird-of-paradise species to explore patterns of evolution across the birds-of-paradise compared to other passerines, and identify a list of candidate genes that may have contributed to the diverse phenotypes seen within the radiation.

This paper is well-written and clear, but quite speculative and would perhaps be nicer as a shorter report with a reduced discussion. Realistically, an exploration of this nature will always feel speculative and like a bit of a gene list, and the authors acknowledge that the resources they are providing in this paper could set up some very interesting future studies. I agree with this. I think that the methods that the authors have used are sound, and that the genes they have identified are interesting new candidate genes to explore with respect to the incredible radiation that is the birds-of-paradise.

Regarding the discussion of TEs, I agree with the authors that the inclusion of a female in their study likely resulted in this higher repeat density in Lycocorax pyrrhopterus than the other species and I would caution the authors on inferring too much based on this result until they have comparable whole genome data from a male of this species.

Other comments:

Page 2, Line 52 - As written "…more or less strong sexual dimorphism" is a little unclear. You're saying that, in general, these clades generally exhibit strong sexual dimorphism, right? I would reword for clarity.

Page 7 Line 21 -Is there really no way to correct for multiple tests here? Seems to me these could all just be random given the sample size.

Page 9 Line 19 - I would say pigment-based rather than pigmentary

Page 10 Line 48 - 52 - how could positive selection not have played a role in morphological evolution? This seems like a throw away section.

Page 11 Line 20 - 31 - the papers that have not found co-evolution between coloration and vision have examined opsin genes almost exclusively, not the genes found to be under positive selection in this paper. I agree that you're speculating here, but I think the connection between selection on vision genes and the displays and coloration of the birds-of-paradise is probably real.

Page 12 Line 4 - I don't know that the authors should be so literal in their interpretation of the startle response results. Leks are more obvious, yes, and so predator evasion is important (What eats an adult bird of paradise?). Startle response is probably also important for interactions with other males in a lek and with visiting females. I would rather this part of the discussion was not just focused on the higher predation likelihood and more holistically discussed startle response in the context of bird-of-paradise lek behavior.

Page 12 Line 22 - I think the "Other positively selected genes and enriched GO categories" section could be much reduced. Include the function of genes in the list of genes under positive selection rather than including that in the main text here.
Page 17 Line 39 - excess, not access?
Supplementary Table 3 - do intron size and number really not vary between the genomes?

Methods

Are the methods appropriate to the aims of the study, are they well described, and are necessary controls included? Choose an item.

Conclusions

Are the conclusions adequately supported by the data shown? Choose an item.

Reporting Standards

Does the manuscript adhere to the journal's guidelines on minimum standards of reporting? Choose an item.

Choose an item.

Statistics

Are you able to assess all statistics in the manuscript, including the appropriateness of statistical tests used? Choose an item.

Quality of Written English

Please indicate the quality of language in the manuscript: Choose an item.

Declaration of Competing Interests

Please complete a declaration of competing interests, considering the following questions:

- Have you in the past five years received reimbursements, fees, funding, or salary from an organisation that may in any way gain or lose financially from the publication of this manuscript, either now or in the future?
- Do you hold any stocks or shares in an organisation that may in any way gain or lose financially from the publication of this manuscript, either now or in the future?
- Do you hold or are you currently applying for any patents relating to the content of the manuscript?
- Have you received reimbursements, fees, funding, or salary from an organization that holds or has applied for patents relating to the content of the manuscript?
- Do you have any other financial competing interests?

- Do you have any non-financial competing interests in relation to this paper?

If you can answer no to all of the above, write 'I declare that I have no competing interests' below. If your reply is yes to any, please give details below.

No

I agree to the open peer review policy of the journal. I understand that my name will be included on my report to the authors and, if the manuscript is accepted for publication, my named report including any attachments I upload will be posted on the website along with the authors' responses. I agree for my report to be made available under an Open Access Creative Commons CC-BY license (http://creativecommons.org/licenses/by/4.0/). I understand that any comments which I do not wish to be included in my named report can be included as confidential comments to the editors, which will not be published.

I agree to the open peer review policy of the journal

To further support our reviewers, we have joined with Publons, where you can gain additional credit to further highlight your hard work (see: https://publons.com/journal/530/gigascience). On publication of this paper, your review will be automatically added to Publons, you can then choose whether or not to claim your Publons credit. I understand this statement.

Yes Choose an item.
