# Supplementary material for: Comparative analyses identify genomic features potentially involved in the evolution of birds-of-paradise
Source: Gigascience. 2019 Jan 24;8(5):giz003. doi: 10.1093/gigascience/giz003 (PMC6497032; doi:10.1093/gigascience/giz003)
Supplement: Reviewer_2_Report_Original_Submission -- Dominic Wright — 6/7/2018 Reviewed [file giz003_reviewer_2_report_original_submission.pdf]

Reviewer Report

Title: Comparative analyses identify genomic features potentially involved in the evolution of birds-of-paradise.

Version: Original Submission **Date:** 6/7/2018

Reviewer name: Dominic Wright

Reviewer Comments to Author:

This is an interesting study that looks at the genomics of five different species of bird of paradise. These are extremely interesting, and under-studied, set of species (as the authors note), and the initial sequencing of these birds can certainly help to resolve the phylogeny surrounding them.

The study itself has two main aspects - firstly to resolve the phylogeny of these birds by sequencing three (and resequencing two) species, and secondly to attempt to identify the genes that underlie the range of different phenotypes that are exhibited by this set of species. The phylogeny resolution results and the conclusions drawn from them are sound and not over-interpreted. The major issues with this manuscript concern the search for positive selection and the use of GO terms to extrapolate the genes underlying the various phenotypes.

Major Issues

1) In the search for positively selected genes the authors identify 178 genes using a nominal p-value significance if I understand their methods correctly, as they state the distribution of p-values gave an excess of non-significant p-values so therefore felt it was not appropriate to use a bonferonni or other multiple testing correction. Given that they test 8133 genes, some form of multiple testing correction is definitely required. Permutation testing seems the most obvious approach as that will be robust against non-normal distributions (see Nielsen, RCD et al, 2005, A scan for positively selected genes in the genomes of humans and chimpanzees. PloS Biology 3:723-733 for one example, but I am sure may others exist).

2) In terms of using this technique (dN/dS ratios) to identify single genes that are being positively selected, this technique is more robust when all the genes in a genome are considered more to identify overall patterns (see Hartl and Clark 'Principles of Population Genetics'). By only having one representative per species as in this case, it should be mentioned that some of the variation present for that species may be being overlooked, which can bias the estimate accordingly.

3) The use of genes with some signature of positive selection as a means of determining genes underlying phenotypic traits is almost certainly over-extrapolated here. By taking a laundry list of genes and then picking potentially interesting putative functions and ascribing them to different traits without further proof is over-speculation (e.g. collagen and extracellular matrix genes are identified, but these genes can potentially affect a gamut of different functions, the authors then say that they are possibly affecting feather formation, craniofacial development, etc). As a further example, the authors state structural colours as being typified by these birds, and that some of these genes are potentially related to structural (and other) colouration. However, no actual genes for structural colours have been identified that I am aware of, so it is unlikely that any genes identified by previous means are the exact

ones that control structural colour variation. In general the traits the authors state are of interest are complex quantitative traits, so the QTL controlling the inter-specific variation are most likely in non-coding regions, and depending on the linkage disequilibrium in the genomes of these birds, easily missed in any case when only considering coding regions. Therefore without additional analysis this speculation over gene function should be drastically reduced.

4) Similarly, looking at GO enriched terms can also easily be extrapolated. For example, in a study by Pavlidis, P. et al. (A critical assessment of storytelling: GO categories and the importance of validating genomic scans. Mol Biol Evol 29(10):3237-3248, 2012), a set of selective sweeps were generated at random in a genome. In each instance, the genes were then analysed for GO enrichment and plausible functions ascribed in each case. This means that GO terms should be used as a complement to, rather than as the basis for the whole study.

In summary, the novel aspects of the sequencing of these genomes is very interesting, their use to resolve the phylogeny is strong, and they are an important step in being able to bring the full power of modern genomics to bear on these species. However, the use of just single representatives of each species to then attempt to prescribe putative phenotypic functions and relationships between the genes that are identified purely based on dN/dS ratios and then speculations regarding the genes identified is highly speculative and undermines the study as it currently is.

Methods

Are the methods appropriate to the aims of the study, are they well described, and are necessary controls included? Choose an item.

Conclusions

Are the conclusions adequately supported by the data shown? Choose an item.

Reporting Standards

Does the manuscript adhere to the journal's guidelines on [minimum standards of reporting?]() Choose an item.

Choose an item.

Statistics

Are you able to assess all statistics in the manuscript, including the appropriateness of statistical tests used? Choose an item.

Quality of Written English

Please indicate the quality of language in the manuscript: Choose an item.

Declaration of Competing Interests

Please complete a declaration of competing interests, considering the following questions:

- Have you in the past five years received reimbursements, fees, funding, or salary from an organisation that may in any way gain or lose financially from the publication of this manuscript, either now or in the future?
- Do you hold any stocks or shares in an organisation that may in any way gain or lose financially from the publication of this manuscript, either now or in the future?
- Do you hold or are you currently applying for any patents relating to the content of the manuscript?
- Have you received reimbursements, fees, funding, or salary from an organization that holds or has applied for patents relating to the content of the manuscript?
- Do you have any other financial competing interests?
- Do you have any non-financial competing interests in relation to this paper?

If you can answer no to all of the above, write 'I declare that I have no competing interests' below. If your reply is yes to any, please give details below.

I declare I have no competing interests

I agree to the open peer review policy of the journal. I understand that my name will be included on my report to the authors and, if the manuscript is accepted for publication, my named report including any attachments I upload will be posted on the website along with the authors' responses. I agree for my report to be made available under an Open Access Creative Commons CC-BY license (http://creativecommons.org/licenses/by/4.0/). I understand that any comments which I do not wish to be included in my named report can be included as confidential comments to the editors, which will not be published.

I agree to the open peer review policy of the journal

To further support our reviewers, we have joined with Publons, where you can gain additional credit to further highlight your hard work (see: https://publons.com/journal/530/gigascience). On publication of this paper, your review will be automatically added to Publons, you can then choose whether or not to claim your Publons credit. I understand this statement.

Yes Choose an item.
