# Supplementary material for: Comparative analyses identify genomic features potentially involved in the evolution of birds-of-paradise
Source: Gigascience. 2019 Jan 24;8(5):giz003. doi: 10.1093/gigascience/giz003 (PMC6497032; doi:10.1093/gigascience/giz003)
Supplement: Reviewer_3_Report_Original_Submission -- Jelmer Poelstra — 6/11/2018 Reviewed [file giz003_reviewer_3_report_original_submission.pdf]

Reviewer Report

Title: Comparative analyses identify genomic features potentially involved in the evolution of birds-of-paradise.

Version: Original Submission **Date:** 6/11/2018

Reviewer name: Jelmer Poelstra

Reviewer Comments to Author:

In this manuscript, Prost et al. perform whole-genome sequencing of five species of birds-of-paradise. Three of these are assembled de-novo, while the other two are mapped to one of the de-novo assembles genomes; all five are subsequently annotated. Special attention is paid to the evolution of repeats, while other analyses include phylogenetic relationships among the birds-of-paradise, the detection of positive selection and gene family size evolution. Genes inferred to have evolved under positive selection, as well as those being among rapidly evolving gene families, were next subjected to Gene Ontology tests, and a substantial part of the Discussion relates to specific genes and gene families whose evolution may have been relevant to that of birds-of-paradise.

I think the paper contains appropriate and solid analyses, and only have relatively minor comments. I do not find the results of the GO and related analyses particularly arresting and thought the Discussion was at parts quite dull while discussing gene after gene, but I realize the limitations of what one can do in genome papers like this one - I think that the authors overall strike a reasonable balance, and do not make unwarranted statements (except regarding TE evolution - see below). I would consider the overall writing somewhat subpar, as the paper contains relatively many errors and some sloppy, uncareful writing - some instances are pointed out in the specific comments below. Generally, I would recommend a careful rereading of the manuscript by the authors.

Specific comments:

* I would suggest to make Table S1 into Table 1, i.e. to put it in the main part of the paper, and potentially to even combine it with Table S3 (and put the resulting table in the main part of the paper). The genome assembly and annotation is the backbone of this paper.

* p. 3, l. 30-34: "Fortunately, the current revolution in sequencing technologies and laboratory methods does not only enable us to sequence whole-genome data from non-model organisms, but it also allows us to harvest genome information from specimens in museum collections [10]." - This point, made in the Introduction, is not being returned to elsewhere in the paper, as far as I could see. I would appreciate it if the authors could make clear in Methods, Results and/or Discussion what it is, specifically, in recent technology/methods that facilitated the use of museum specimens in this manuscript, and perhaps also discuss the promise/limitations/future developments in this area.

* Discussion section "Repeats and their possible role in the evolution of birds-of-paradise" (page 6-7). I find this section quite speculative and insufficiently carefully worded.

- First, "A growing body of literature is emerging" - this sentence only cites a 9 year-old and a 10 year-old paper, which is not very convincing support of that statement.

- Second, to see if the paper cited in the next sentence (ref. 30, which is from 2011) would provide an

additional and more recent reference supporting the statement in the previous sentence, I had a look at it. It is cited as "Bursts of TE activity are often lineage and species-specific, which highlights their potential role in speciation", but the words "TE", "transposable", or even "repeat" or not to be found in this paper. Please clarify/change accordingly.
- "The timing [of a burst of TE activity] fits the emergence and radiation of birds-of-paradise". The fact that the emergence (ca. 35 ma) and radiation (I guess somewhere between 10 and 20-25 ma) are mentioned here already indicates that this statement is quite general, and I think a similar statement could have been made if the burst of TE activity fell anywhere between 10 and about ma 40 ago. The question thus is how meaningful this coincidence in timing really is, especially considering that, presumably, the likely statistical inference of such TE bursts may restricted to a certain time window (?).
- "It is thus likely that the diversification of birds-of-paradise was influenced by lineage specificity of their TE repertoires through retroviral germline invasions and smaller activity bursts". An in my view completely unwarranted statement, mainly due to the qualifier "likely". Instead, I would suggest a qualifier along the lines of "An intriguing hypothesis that warrants further investigation".
* In the discussion of genes and GO categories, please state more clearly in which GO test the genes in question were flagged: positive selection or gene family size evolution.
* p. 6, l. 34-36: "Dale and colleagues recently showed that sexual selection on male ornamentation in birds has antagonistic effects, where male coloration is increasing, while females show a strong reduction in coloration [37]. This is very apparent in polygynous core birds-of-paradise, where females between species and sometimes even between genera look highly similar." Increasing and decreasing "coloration"? "Ornamentation" would be a better choice of word. Also, a reduction in ornamentation/coloration is not the same thing as a reduced tempo of evolution, which is clearly referred to in the second sentence. Thus, expand/clarify.
* Methods, Gene Annotation section: "We did not train the de novo gene predictor Augustus [109] because no training data set for birds was available." Please expand - none of the gene sets from previously published bird genomes can be used?
* Methods, Phylogenetic Analysis section: "We used the individual exon phylip files for gene tree reconstruction" - Why were only single exons used for each gene? I worry whether these were sufficiently informative, especially considering the high discordance among gene trees.
Minor comments:
* Italicize genus names throughout the paper.
* p.2, l. 19: "Ernst Mayer" - Remove the "e" in Mayer.
* p. 3, l. 4-6: Change discussion of Irestedt et al. 2009 findings to past tense.
* p. 3, l. 14: "specialized on" - reformulate.
* p. 3, l. 34: "Only recently, have these" - Remove comma.
* p. 3, l. 59-60: "All assemblies showed a genome size around 1 Gb": Assembly size, not genome size. Also, give the range of assembly lengths, and refer to Table S1.
* p. 5, l. 8: "taxa" - change to "taxon".
* p. 8, l. 43-46: Sentence starting with "Congruently" - rephrase. In the next sentence, delete "the".
* p. 10, l. 57: "On the contrary to other vertebrates" - Change to "Contrary to ...".
* p. 11, l. 41: "straight forward" - Change to "straightforward".
* Methods, Ortholog Gene Calling section: "For a gene to be included in our analyses, it had to be

present in at least 75% of all species (7 out of 8 species)" - 75% would be 6 (not 7) out of 8 species…
* Methods, Intron Calling section: "To do so we used the extract_intron_gff3_from_gff3.py script (https://github.com/irusri/Extract-intron-from-gff3) to include intron coordinates into the gff file. We then parsed out all intron coordinates and extracted the intron sequences from the genomes using the exttract_seq_from_gff3.pl script (https://github.com/irusri/Extract-intron-from-gff3)." - The same script is referenced twice in the actual link, but with a different name. Furthermore, I assume "exttract_seq_from_gff3.pl" has a typo ("exttract").
* Methods, Inference of Positive Selection section: "due to the fact that branch-site tests result in an access of non-significant p-values" - Change "access" to "excess".

Methods

Are the methods appropriate to the aims of the study, are they well described, and are necessary controls included? Choose an item.

Conclusions

Are the conclusions adequately supported by the data shown? Choose an item.

Reporting Standards

Does the manuscript adhere to the journal's guidelines on [minimum standards of reporting?](#) Choose an item.

Choose an item.

Statistics

Are you able to assess all statistics in the manuscript, including the appropriateness of statistical tests used? Choose an item.

Quality of Written English

Please indicate the quality of language in the manuscript: Choose an item.

Declaration of Competing Interests

Please complete a declaration of competing interests, considering the following questions:

- Have you in the past five years received reimbursements, fees, funding, or salary from an organisation that may in any way gain or lose financially from the publication of this manuscript, either now or in the future?
- Do you hold any stocks or shares in an organisation that may in any way gain or lose financially from the publication of this manuscript, either now or in the future?

- Do you hold or are you currently applying for any patents relating to the content of the manuscript?
- Have you received reimbursements, fees, funding, or salary from an organization that holds or has applied for patents relating to the content of the manuscript?
- Do you have any other financial competing interests?
- Do you have any non-financial competing interests in relation to this paper?

If you can answer no to all of the above, write 'I declare that I have no competing interests' below. If your reply is yes to any, please give details below.

I declare that I have no competing interests

I agree to the open peer review policy of the journal. I understand that my name will be included on my report to the authors and, if the manuscript is accepted for publication, my named report including any attachments I upload will be posted on the website along with the authors' responses. I agree for my report to be made available under an Open Access Creative Commons CC-BY license (http://creativecommons.org/licenses/by/4.0/). I understand that any comments which I do not wish to be included in my named report can be included as confidential comments to the editors, which will not be published.

I agree to the open peer review policy of the journal

To further support our reviewers, we have joined with Publons, where you can gain additional credit to further highlight your hard work (see: https://publons.com/journal/530/gigascience). On publication of this paper, your review will be automatically added to Publons, you can then choose whether or not to claim your Publons credit. I understand this statement.

Yes Choose an item.
